# Vicinal Bis(methylene) Heterocyclic Diene in Natural Product Synthesis: A Convergent Biomimetic Total Synthesis of Prunolactone A

**DOI:** 10.1021/acs.orglett.4c04378

**Published:** 2024-12-07

**Authors:** Michal Kadaník, Ekaterina Frantsuzova, Petr Matouš, Lucie Nováková, Jiří Kuneš, Manola Bonsignore, Erik Andris, Zdeňka Růžičková, Milan Pour

**Affiliations:** † Department of Organic and Bioorganic Chemistry, Faculty of Pharmacy, Charles University, Heyrovského 1203, 500 05 Hradec Králové, Czech Republic; ‡ Department of Analytical Chemistry, Faculty of Pharmacy, Charles University, Heyrovského 1203, 500 05 Hradec Králové, Czech Republic; § Institute of Organic Chemistry and Biochemistry, Academy of Sciences of the Czech Republic, Flemingovo náměstí 2, 166 10 Prague 6, Czech Republic; ∥ Department of General and Inorganic Chemistry, University of Pardubice, Faculty of Chemical Technology, Studentská 573, 532 10 Pardubice, Czech Republic

## Abstract

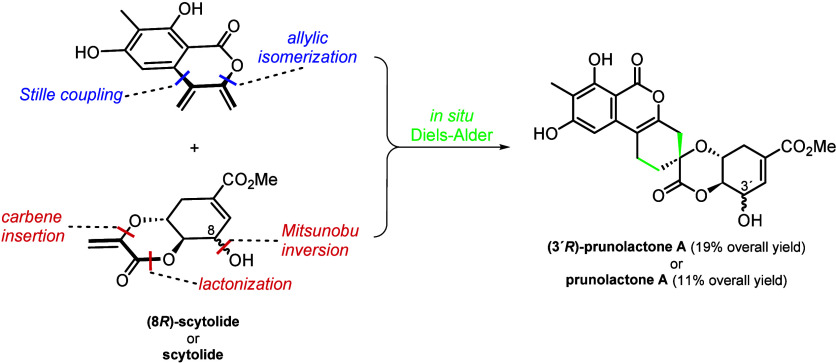

The first total syntheses of the natural isocoumarin prunolactone A with a 6/6/6/6/6 spiropentacyclic skeleton and its unnatural (3′*R*)-epimer in 10 and 8 steps, respectively, are reported. The syntheses feature *in situ* generation of a reactive 3,4-bis(methylene)isocoumarin intermediate, its biomimetic Diels–Alder reactions with the shikimic-acid-derived scytolide and (8*R*)-scytolide, and a Mitsunobu reaction allowing access to scytolide in a stereochemically pure form. Computational support for the selectivity of the Diels–Alder reaction is provided.

Multi-bond-forming, atom-economical reactions are the most efficient processes for the synthesis of complex natural products. Two vicinal exocyclic methylenes, incorporated in a heterocycle,^1^ thus represent an excellent tool for the construction of heterocyclic scaffolds, since their methylenes are naturally locked in the *s*-cis conformation, required for a Diels–Alder reaction. Such agents are, therefore, capable of editing or labeling dienophilic sites with various heterocyclic moieties under mild conditions.

Nature has been recently shown to utilize the same strategy, as exemplified by the recent isolations of cuautepestalorin^2^ and prunolactones^3^ (Figure 1), formed via 3,4-bis(methylene)isocoumarin intermediates (*vide infra*). In this Letter, we report *in situ* generation of a 3,4-bis(methylene)isocoumarin synthon (**A**, Scheme 1) and demonstrate its reactivity on the reaction with scytolide and (8*R*)-scytolide resulting in the total syntheses of prunolactone A and its (3′*R*)-epimer.

**Figure 1:**
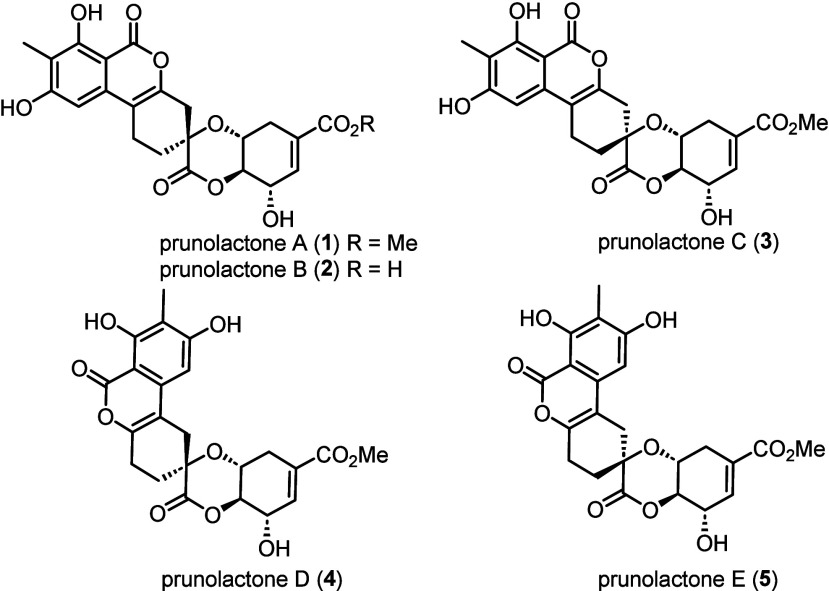
Structures of prunolactones A–E (**1**–**5**).

**Scheme 1:**
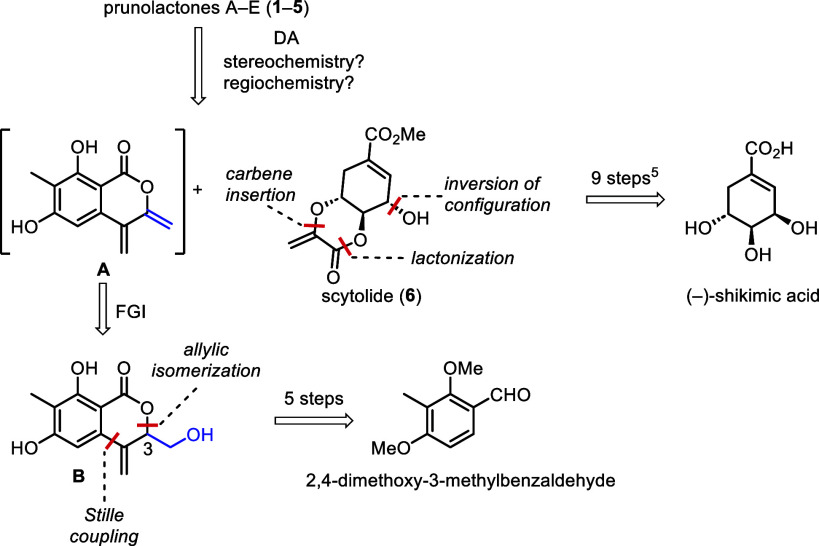
Retrosynthetic Analysis of Prunolactones A–E **(1–5)**

Prunolactones, a set of seven structurally unprecedented isocoumarins with a densely functionalized 6/6/6/6/6 spiropentacyclic skeleton (see Figure 1 for the structures of prunolactones A–E), were isolated^3^ from the endophytic fungus *Phomopsis prunorum* in 2023. Some of the compounds were found to possess proangiogenic activity in an animal model,^3^ and their production via cultivation of the fungus on rice was patented^4^ earlier this year.

As postulated by Zhang and Guo,^3^ prunolactones A (**1**), C (**3**), D (**4**), and E (**5**) can be derived from a single Diels–Alder reaction between scytolide (**6**) and 3,4-bis(methylene)isocoumarin **A** (Scheme 1), depending on its stereochemistry (e.g., prunolactones A and C) and regiochemistry (e.g., prunolactones A and D).

Since the preparation of scytolide (**6**) from shikimic acid in 9 steps has been reported,^5^ the success of the total synthesis depended on a facile access to synthon **A**. As the high reactivity (and hence limited stability) of the diene fragment in **A** was anticipated, further functional group interconversion led to synthon **B** with a C3 hydroxymethyl group. This (or an equivalent) group could be subjected to elimination, furnishing **A**. Finally, we envisaged constructing isocoumarin derivative **B** via Pd black-catalyzed tandem cross-coupling/allylic isomerization.^6,7^

As regard to suitable coupling partners, (*E*)-2-tributylstannylbut-2-ene-1,4-diol **7** was prepared by the hydrostannylation of but-2-yne-1,4-diol,^6^ while the aryl halide coupling partner **8** was obtained in three steps from the commercially available 2,4-dimethoxy-3-methylbenzaldehyde. This compound was subjected to C6 iodination furnishing the known^8^ 6-iodo-2,4-dimethoxy-3-methylbenzaldehyde, which was further converted to methyl 6-iodo-2,4-dimethoxy-3-methylbenzoate **8** via standard oxidation and esterification (see the SI) in 48% overall yield. The results of the coupling attempts are summarized in Table 1.

**Table 1:**
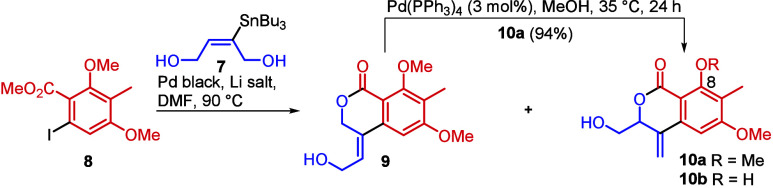
Cross-Coupling and Allylic Isomerization

Entry	**7** (eq)	Pd black (mol %)	Li salt (eq)	Reaction time (h)	Pyranones **9**/**10a**/**10b** (% yield)
1	1.3	2	-–	24	**9** (30)/**10a** (9)
2	2.5	4	LiCl (3)	72	**9** (5)/**10a** (48)/**10b** (20)
3^a^	2.5	4	LiCl (5)	72	**9** (6)/**10a** (52)/**10b** (26)
4	2.5	4	LiI (2.5)	72	**9** (32)/**10a** (34)

a Freshly dried LiCl under Ar.

While the protocol using just 2% Pd black (entry 1) led to a low yield of coupling product **9** (30%), we also observed some degree of rearrangement of **9** into **10a** under these conditions. Hence, in order to accelerate the process, we increased the amount of diol **7** to 2.5 equiv as well as the loading of Pd black to 4%, and we used Li salts as additives.^6,9−11^ The data in entries 2, 3, and 4 show apparent influence of the Li salts on the reaction outcome. While the addition of LiI (entry 4) gave rise to a mixture of **9** and **10a** in a good overall yield (32 and 34%, respectively), the use of LiCl accelerated the allylic isomerization but also effected partial demethylation of the C8 methoxy group (entry 3). The data in entry 3 show that using the combination of 4% of Pd black/5 eq of freshly dried LiCl enabled us to execute both cross-coupling and allylic isomerization in one step, together with the partial deprotection of the C8 methoxyl.

Since limited stability of the spirocyclic lactone ring in the prunolactones in a mere MeOH solution was reported,^3^ we decided to deprotect both methoxy groups in the next operation to avoid exposure of the compounds to harsh conditions in the final step. The formation of the mixtures of **9**, **10a**, and **10b** was therefore not an issue, especially because the remaining pyranone **9** could be smoothly rearranged^7^ into isocoumarin **10a** in high yield (94%) using Pd(PPh_3_)_4_ (see Table 1). Hence, the mixture of **10a** and **10b** was treated with BBr_3_ to yield completely deprotected pyranone **11** (Scheme 2), corresponding to projected synthon **B** (Scheme 1).

**Scheme 2:**
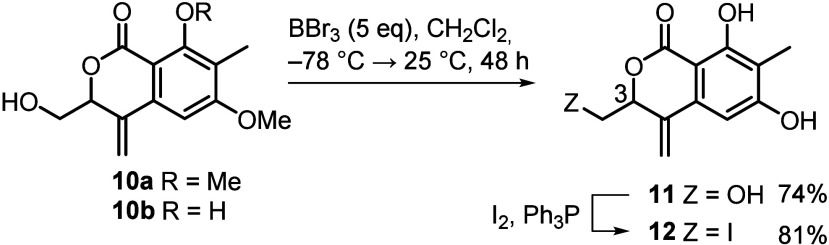
Preparation of Synthon B (**11**)

The primary OH group in **11** was smoothly replaced with iodine to furnish 3-iodomethyl derivative **12**, a precursor of synthon **A**. With compound **12** in hand, preparation of a scytolide (**6**) was the next task. Milzarek and Gulder^5^ reported a 9 step synthesis of the natural product from shikimic acid,^13^ but the last step involved inversion of configuration at C8 *via* oxidation to an unstable ketone, and its reduction to afford inseparable mixtures of scytolide (**6**) with the starting compound, its (8*R*)-epimer (**13**), in a 5:1 ratio in 90% overall yield (Scheme 3). Since in our hands this procedure gave a ratio of just 2:1 with a total yield of 52%, and the application of other reduction agents provided no improvement (see Table S1 in the SI), we sought access to stereochemically pure **6**.^12^

**Scheme 3:**
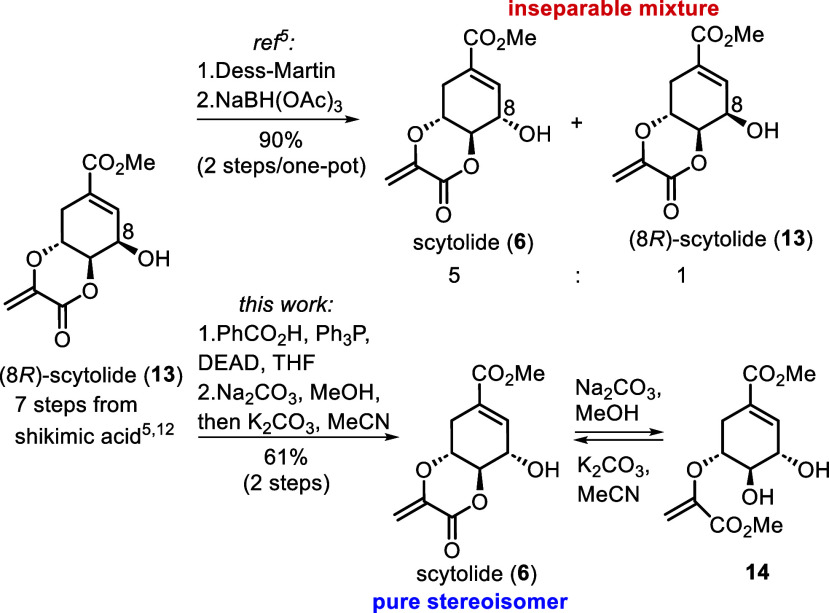
Access to Stereochemically Homogeneous Scytolide (**6**)

To our delight, a Mitsunobu reaction effected inversion of configuration at C8 and delivered scytolide (**6**) in equilibrium with the hydroxy ester **14**, formed via base-mediated lactone ring opening. The latter was easily recyclized to scytolide (**6**) by K_2_CO_3_/MeCN to give a stereochemically pure product in 61% yield over two steps (Scheme 3), the physical data of which were in complete agreement with the literature, with the exception of optical rotation. While the existing reports give negative [α]_D_ values for scytolide (**6**), namely −37.7° (c 0.26, MeOH, temperature not given),^14^ −63.2° (c 0.30, MeOH, 25 °C),^15^ and −30.6° (c 0.11, CHCl_3_, 20 °C),^5^ we repeatedly recorded the value of +38.0° (c 0.11, CHCl_3_, 25 °C), +40.0° (c 0.50, MeCN, 25 °C), and +38.5° (c 0.26, MeOH, 25 °C). To exclude any doubts, X-ray and ECD analyses (see the SI) unequivocally confirmed the identity of our product as a scytolide (**6**).

The stage was thus set for the crucial DA reaction. Screening of the reaction conditions (Table 2) identified aliphatic tertiary amines as the bases of choice to trigger the elimination of **12**. The putative intermediate **15** (synthon **A**, Scheme 1) was then trapped in a DA reaction with a surplus of **6** (1.5 or 3.0 equiv) to afford a single product. The compound was assigned the structure of prunolactone A (**1**), based on 2D spectra and comparison of its ^13^C spectrum with those of prunolactones A, C, D, and E. DABCO (entry 2), DIPEA (entry 4), and Et_3_N (entry 6) gave comparable yields (55–59%) together with recovered scytolide (66–72%), while a stronger base (DBU) afforded a mixture of products with just 15% **1** (entry 7). With 10 equiv of pyridine (entry 9), mere traces of the product were detected. The yield of 57% was obtained only when pyridine was employed as the solvent (entry 11). Similarly, using (8*R*)-scytolide (**13**) bearing the “natural” configuration of shikimic acid, the unnatural (3′*R*)-epimer of prunolactone A (**16**) was constructed under optimized conditions with 61% yield. Comparison of the ^13^C NMR spectrum of (3′*R*)-prunolactone A with that of prunolactone A revealed a significant difference of the chemical shifts of their C3′ carbons (63.40 and 68.81 ppm, respectively), while those of the spiro carbons were nearly the same (76.20 and 76.23 ppm, respectively).

**Table 2:**
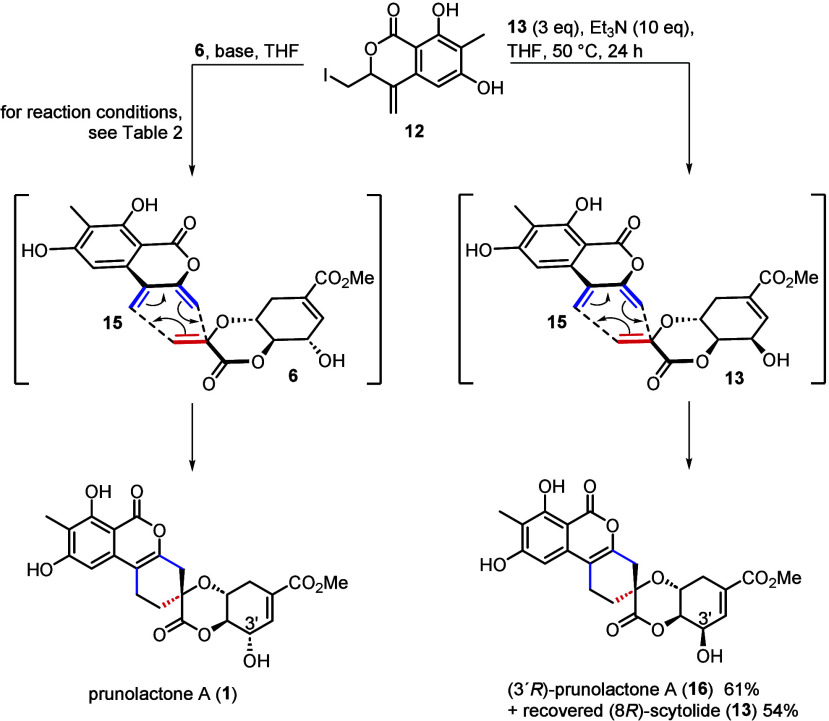
Assembly of Prunolactone A (**1**) and Its Unnatural (3′*R*)-Epimer (**16**) in a Biomimetic DA Reaction

Entry	**6** (eq)	Base (eq)	Reaction conditions	Yield of **1** (%)	Recovered **6** (yield %)
1	1.5	DABCO (3)	24 h, 25 °C	46	49
2	3.0	DABCO (3)	24 h, 25 °C	57	69
3	1.5	DIPEA (10)	48 h, 25 °C	32	63
4	3.0	DIPEA (10)	24 h, 50 °C	55	72
5	1.5	Et_3_N (10)	24 h, 50 °C	44	47
6	3.0	Et_3_N (10)	24 h, 50 °C	**59**	66
7	1.5	DBU (3)	15 min, 25 °C	15^a^	73
8	1.5	pyridine (10)	24 h, 25 °C	n/d^b^^,^^c^	–
9	1.5	pyridine (10)	24 h, 50 °C	traces^c^	–
10	1.5	pyridine^d^	48 h, 50 °C	53	58
11	3.0	pyridine^d^	48 h, 50 °C	57	72

a The rest was an intractable mixture.

b Product not detected.

c SM recovered.

d Pyridine used as a solvent.

In order to explain the outcome of the DA reaction, we performed exploratory density functional theory (DFT) calculations of this reaction step at ωB97X-D^16^/6-311+g(2d,p)//6-31g(d,p) level with the CPCM solvation model in Gaussian 16 program^17^ (details in the SI). All transition states (Figure 2) prefer a geometry where the ether oxygen of the scytolide is in *endo* position with respect to the diene **15**. Moreover, they are all quite asymmetric; the distance between the ring carbon of the dienophile and the corresponding carbon in the diene is ∼2.9 Å in **TS_1,3** and ∼2.7 Å in **TS_4,5**, whereas the distance between the peripheral dienophile carbon and the corresponding diene carbon is in the 2.02 to 2.07 Å range. Furthermore, we observed relatively short contacts between the hydrogen atom of the cyclohexene unit in the scytolide and the planar diene (2.5–2.7 Å range), including a possible weak CH···O hydrogen bond in **TS_1** and possible weak(er) CH···π interactions in **TS_3,4,5**. The degree of ring stacking seems to grow from **TS_1** to **TS_5**. Relative electronic energies of **TS_1,3,4,5** with respect to **TS_1** are 0.0, 1.1, −1.0, and −0.9 kcal mol^–1^, respectively. Therefore, the electronic energies themselves suggest either **4** and **5** as the dominant products. However, the predicted Gibbs free energies (298 K and 1 M) of transition states, which include entropic contributions, show a different order: 19.1 (leading to product **1**), 20.0 (**3**), 20.5 (**4**), and 21.4 (**5**) kcal mol^–1^. Therefore, it is mainly entropic contributions that steer the reaction to its preferred isomer **1**.

**Figure 2:**
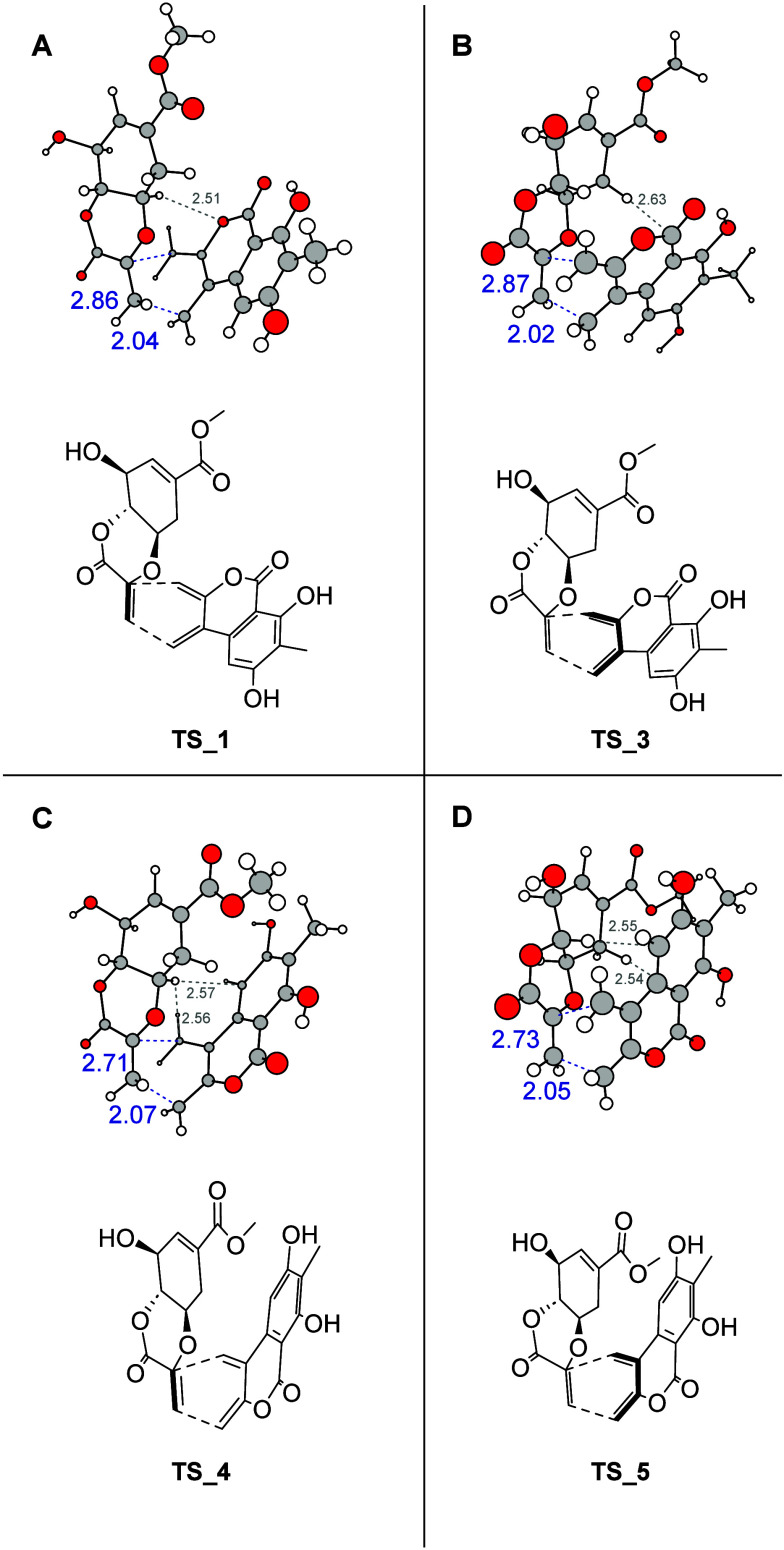
(A–D) Structures of most stable transition states leading to **1**, **3**, **4**, and **5** (prunolactones A, C, D, and E, respectively). The numbers indicate bond lengths (in Å) in the transition states. The bonds that are formed in the DA reaction are shown in blue. In each panel, the top part represents the 3D structure, while the lower part is a corresponding 2D structural formula.

In summary, we have successfully generated the reactive 3,4-bis(methylene)isocoumarin intermediate (**15**), which can be employed for labeling various dienophiles with the isocoumarin core, well-known for its biological^18^ and photophysical^19^ properties. The diene was utilized in a regio- and stereoselective total synthesis of the natural product prunolactone A via a biomimetic Diels–Alder reaction as the key step. Also of note, the diene possessed free phenolic groups in order to avoid exposure of the rather fragile prunolactone skeleton to harsh demethylation conditions in the last operation. Finally, the synthesis is convergent, with the longest linear sequence being composed of 10 steps (and 8 steps for the unnatural epimer). Prunolactone A and its (3′*R*)-epimer were thus prepared from inexpensive shikimic acid in 11 and 19% overall yields, respectively. DFT calculations suggest that preferential formation of prunolactone A is due to the greater entropy of its transition state, compared to other possible products.^20^ However, the energies are overall quite similar, and no strong effects are involved. Further application of the strategy to other members of the prunolactone family and refinement of the computational model are in progress.

## Data Availability

The data underlying this study are available in the published article and its Supporting Information.

## Supporting Information Available

Experimental procedures and spectroscopic data for all compounds, X-ray crystallographic data and ECD spectrum of scytolide (**6**), and DFT-computational details (PDF); structures of DFT-calculated compounds (XYZ); all optimized conformers for **TS_1** (Log.gz) (ZIP)
FAIR data, including the primary NMR FID files, for compounds **1**, **6**–**14**, **16** and **S1**–**S10** (ZIP)
