# Supplementary material for: Vicinal Bis(methylene) Heterocyclic Diene in Natural Product Synthesis: A Convergent Biomimetic Total Synthesis of Prunolactone A
Source: Org Lett. 2024 Dec 7;26(50):11080–4. doi: 10.1021/acs.orglett.4c04378 (PMC11667730; doi:10.1021/acs.orglett.4c04378)
Supplement: Supplementary file 2 — ol4c04378_si_002.zip [file ol4c04378_si_002.zip › ol4c04378_SI/Supporting_information.pdf]

Supporting information for

Vicinal Bis(methylene) Heterocyclic Diene in Natural Product Synthesis: A Convergent Biomimetic Total Synthesis of Prunolactone A

Michal Kadaník,[a] Ekaterina Frantsuzova,[a] Petr Matouš,[a] Lucie Nováková,[b] Jiří Kuneš,[a] Manola Bonsignore,[c] Erik Andris,[c] Zdeňka Růžičková,[d] and Milan Pour[a*]

[a] *Department of Organic and Bioorganic Chemistry, Faculty of Pharmacy, Charles University, Heyrovského 1203, 500 05 Hradec Králové, Czech Republic*

[b] *Department of Analytical Chemistry, Faculty of Pharmacy, Charles University, Heyrovského 1203, 500 05 Hradec Králové, Czech Republic*

[c] *Institute of Organic Chemistry and Biochemistry, Academy of Sciences of the Czech Republic, Flemingovo náměstí 2, 166 10 Prague 6, Czech Republic*

[d] *Department of General and Inorganic Chemistry, University of Pardubice, Faculty of Chemical Technology, Studentská 573, 532 10 Pardubice, Czech Republic*

Email: pour@faf.cuni.cz

Table of Contents

1 General Information S2
2 Experimental Procedures S2
2.1 Synthesis of Iodide **12** S2
2.2 Synthesis of (8*R*)-Scytolide (**13**) S7
2.3 Synthesis of Scytolide (**6**) S11
2.3.1 Mitsunobu Inversion of (8*R*)-Scytolide (**13**) S11
2.3.2 Oxidation-Reduction Method S13
2.4 Diels-Alder Reactions S14
3 ^{1}H and ^{13}C NMR Spectra of the Compounds S16
4 NMR Data of Prunolactone A (**1**) S40
4.1 Comparison of ^{1}H and ^{13}C NMR Spectra of Synthetic and Natural Prunolactone A (**1**) S40
4.2 2D NMR Spectra of Prunolactone A (**1**) S41
5 Additional Analysis of Scytolide (**6**) S43
5.1 X-Ray Analysis of Scytolide (**6**) S43
5.2 ECD Analysis of Scytolide (**6**) S45
6 DFT Calculations S45
6.1 Computational Details S45
6.2 Descriptions of File Formats S45
7 References S46

1 General Information

All reagents and solvents were purchased from Sigma-Aldrich (Merck, KGaA, Darmstadt, Germany) and used without further purification. Solvents (DCM, THF) were dried prior to use (PureSolv PS-Micro, Innovative Technologies, USA). The reactions were carried out in oven-dried glassware using Schlenk line techniques with magnetic stirring and dried solvents under Ar atmosphere. TLC analyses were performed using Merck TLC Silica gel F254 TLC plates and visualized by UV (254 nm) in combination with staining (using the solution of $Ce(SO_4)_2 \cdot 4H_2O$ (2 g), $H_3[P(Mo_3O_{10})_4]$ (4 g), conc. H_2SO_4 (10 mL) and H_2O (200 mL) with subsequent heating). Column chromatography was carried out on Merck Silica gel 60 (0.040–0.063 mm) and Merck LiChroPrep RP-18 (25–40 μm). ^{1}H and ^{13}C NMR spectra were recorded with a Varian VNMR S500 or Jeol JNM-ECZ600R instrument. The chemical shifts were recorded as δ values in parts per million (ppm), reported relative to TMS and referenced to the residual solvent peaks ($CDCl_3$: 7.24 ppm for ^{1}H NMR, 77.0 ppm for ^{13}C NMR; CD_3OD: 3.30 ppm for ^{1}H NMR, 49.0 ppm for ^{13}C NMR; acetonitrile-d_6: 1.93 ppm for ^{1}H NMR, 1.3 ppm for ^{13}C NMR; DMSO-d_6: 2.49 ppm for ^{1}H NMR, 39.7 ppm for ^{13}C NMR; acetone-d_6: 2.04 ppm for ^{1}H NMR, 29.8 ppm for ^{13}C NMR). Coupling constants (*J*) are given in Hz. The following abbreviations were used to designate the multiplicities: s = singlet; d = doublet; t = triplet; m = multiplet; br = broad; dd = doublet of doublets; ddd = doublet of doublet of doublets; ddt = doublet of doublet of triplets; td = triplet of doublets. Structural assignments were made with additional information from COSY, HSQC, HMBC and NOESY experiments. IR spectra were recorded on a NICOLET 6700 FT-IR equipped with an ATR device. HRMS data were recorded on an QTOF mass spectrometer using the electrospray ionization. Optical rotation was measured on automatic polarimeter A. Krüss Optronic P3000. ECD spectra were measured on a JASCO J-815 CD spectrometer. Crystallographic data were obtained from a Bruker D8 Venture diffractometer. Melting point was measured on STUART SMP30 Digital Melting Point Apparatus.

2 Experimental Procedures

2.1 Synthesis of Iodide 12

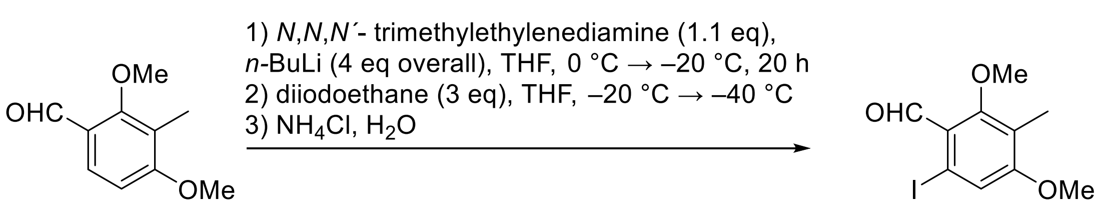

S1 (57%)

6-Iodo-2,4-dimethoxy-3-methylbenzaldehyde (S1): *N,N,N´*-Trimethylethylenediamine (4.65 mL; 34.67 mmol) was dissolved in THF (200 mL) under Ar atmosphere. The solution was cooled to 0 °C and *n*-BuLi (2.5M in hexane; 16.71 mL; 41.76 mmol) was added dropwise. After stirring for 15 min, the reaction mixture was cooled to –20 °C and a solution of 2,4-dimethoxy-3-methylbenzaldehyde (5.68 g; 31.52 mmol) in THF (50 mL) was added. After stirring for

30 min, *n*-BuLi (2.5M in hexane; 33.36 mL; 83.40 mmol) was added dropwise and the mixture was stirred overnight. Afterwards, the solution was cooled to –40 °C and a solution of diiodoethane (26.65 g; 94.56 mmol) in THF (50 mL) was added dropwise. After 5 min, the mixture was warmed to room temperature, and the reaction was quenched by the addition of saturated NH_4Cl solution (100 mL), followed by saturated $Na_2S_2O_3$ solution (40 mL). The organic phase was separated, and the aqueous phase extracted with Et_2O (3 × 120 mL). The combined organic phases were dried over anhydrous Na_2SO_4, and concentrated under reduced pressure. The crude product was purified on silica gel (hexanes/EtOAc 98 : 2) to afford aldehyde **S1** (5.5 g; 57%) as a yellow amorphous solid. All spectral data were in agreement with those reported in the literature[1]. **^{1}H NMR** (500 MHz, $CDCl_3$) δ 10.04 (s, 1H), 7.22 (s, 1H), 3.87 (s, 3H), 3.78 (s, 3H), 2.08 (s, 3H); **^{13}C NMR** (126 MHz, $CDCl_3$) δ 191.5, 162.8, 162.0, 122.3, 121.7, 119.7, 95.0, 62.8, 56.1, 8.5; **IR** (ATR) ν 2970, 2941, 2857, 2771, 1680, 1568, 1470, 1370, 1276, 1230, 1127 cm^{-1}; **HRMS** (TOF-ESI) *m*/*z*: $[M+H]^+$ Calcd. for $C_{10}H_{12}IO_3^+$ 306.9826, Found 306.9835; **R*f*** 0.63 (hexanes/EtOAc 7 : 3).

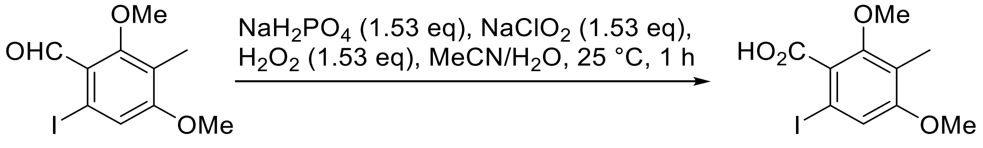

S1 **S2** (91%)

6-Iodo-2,4-dimethoxy-3-methylbenzoic acid (S2): Aldehyde **S1** (5.34 g; 17.45 mmol) was dissolved in a mixture of MeCN and H_2O (5 : 1, v : v; 96 mL). Hydrogen peroxide (30% in H_2O; 2.09 mL; 26.7 mmol) was added dropwise, followed by the addition of $NaH_2PO_4 \cdot 2H_2O$ (4.17 g; 26.7 mmol). The mixture was cooled to 0 °C and a solution of $NaClO_2$ (80 %; 3.02 g; 26.7 mmol) in water (16 mL) was added. The solution was vigorously stirred for 1 h at room temperature. The reaction was quenched by the addition of saturated $Na_2S_2O_3$ solution (5 mL). The organic phase was separated, and the aqueous phase was extracted with Et_2O (3 × 100 mL). The combined organic phases were dried over anhydrous Na_2SO_4 and concentrated under reduced pressure to afford crude acid **S2** (5.11 g; 91%) as a yellow amorphous solid. **^{1}H NMR** (500 MHz, $CDCl_3$) δ 7.08 (s, 1H), 3.82 (s, 3H), 3.81 (s, 3H), 2.09 (s, 3H); **^{13}C NMR** (126 MHz, $CDCl_3$) δ 172.1, 160.2, 156.7, 126.2, 120.8, 117.4, 88.4, 62.4, 56.0, 9.0; **IR** (ATR) ν 2949, 2645, 2559, 2363, 1695, 1583, 1376, 1301, 1286, 1270, 1174, 1119, 1006 cm^{-1}; **HRMS** (TOF-ESI) *m*/*z*: $[M–H_2O+H]^+$ Calcd. for $C_{10}H_{10}IO_3^+$ 304.9669, Found 304.9677; **R*f*** 0.30 (hexanes/EtOAc/AcOH 1 : 1 : 0.01).

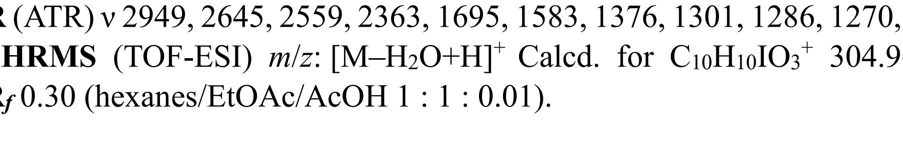

S2 **8** (92%)

Methyl 6-iodo-2,4-dimethoxy-3-methylbenzoate (8): The crude acid **S2** (5.4 g; 16.79 mmol) was dissolved in DMF (40 mL), followed by the addition of K_2CO_3 (3.48 g; 25.18 mmol) and iodomethane (1.57 mL; 25.18 mmol). The mixture was heated to 90 °C in oil bath and stirred for 45 min. The mixture was diluted with EtOAc (60 mL) and washed with 5% HCl

solution (120 mL). The organic phase was dried over anhydrous Na_2SO_4 and concentrated under reduced pressure. The crude product was purified on silica gel (hexanes → hexanes/EtOAc 95 : 5) to afford ester **8** (5.17 g; 92%) as a yellowish oil. **^{1}H NMR** (500 MHz, $CDCl_3$) δ 7.00 (s, 1H), 3.91 (s, 3H), 3.79 (s, 3H), 3.73 (s, 3H), 2.06 (s, 3H); **^{13}C NMR** (126 MHz, $CDCl_3$) δ 168.2, 159.8, 156.4, 128.0, 120.6, 116.6, 87.9, 62.1, 56.0, 52.6, 8.9; **IR** (ATR) ν 3010, 2951, 1723, 1586, 1564, 1432, 1387, 1295, 1277, 1155, 1119, 1006 cm^{-1}; **HRMS** (TOF-ESI) *m/z*: $[M–MeOH+H]^+$ Calcd. for $C_{10}H_{10}IO_3^+$ 304.9669, Found 304.9679; **R*f*** 0.73 (hexanes/EtOAc 7 : 3).

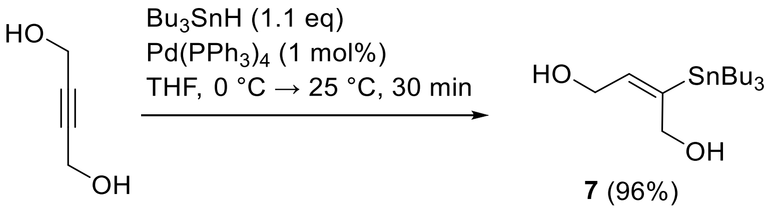

7 (96%)

(*E*)-2-tributylstannylbut-2-ene-1,4-diol (7): But-2-yne-1,4-diol (4.34 g; 50.37 mmol) and $Pd(PPh_3)_4$ (582 mg; 0.50 mmol) were dissolved in THF (50 mL) under Ar atmosphere. The solution was cooled to 0 °C and Bu_3SnH (14.9 mL; 55.4 mmol) was added dropwise. The reaction was stirred for 10 min at 0 °C and the flask was removed from the ice bath. The mixture was then stirred for another 20 min at room temperature. The solvent was evaporated under reduced pressure, and the crude product was purified on silica gel (hexanes → hexanes/EtOAc 7 : 3) to afford diol 7 (18.24 g; 96%) as a yellow oil. All spectral data were in agreement with those reported in the literature[2]. **^{1}H NMR** (600 MHz, $CDCl_3$) δ 5.79–5.75 (m, 1H), 4.40–4.31 (m, 2H), 4.20–4.14 (m, 2H), 1.82 (t, J = 5.2 Hz, 1H), 1.75 (t, J = 5.5 Hz, 1H), 1.55–1.39 (m, 6H), 1.35–1.24 (m, 6H), 0.98–0.83 (m, 15H); **^{13}C NMR** (151 MHz, $CDCl_3$) δ 149.4, 138.1, 63.6, 59.8, 29.1 (J_{Sn} = 19.6 Hz), 27.4 (J_{Sn} = 58.0 Hz), 13.7, 10.0 ($J_{Sn\text{-}119}$ = 340.6 Hz, $J_{Sn\text{-}117}$ = 325.2 Hz); **IR** (ATR) ν 3288, 2955, 2923, 2871, 2852, 1463, 1376, 1028 cm^{-1}; **HRMS** (TOF-ESI) *m/z*: $[M+Na]^+$ Calcd. for $C_{16}H_{34}O_2{}^{120}SnNa^+$ 401.1473, Found 401.1476; **R*f*** 0.68 (hexanes/EtOAc 1 : 1).

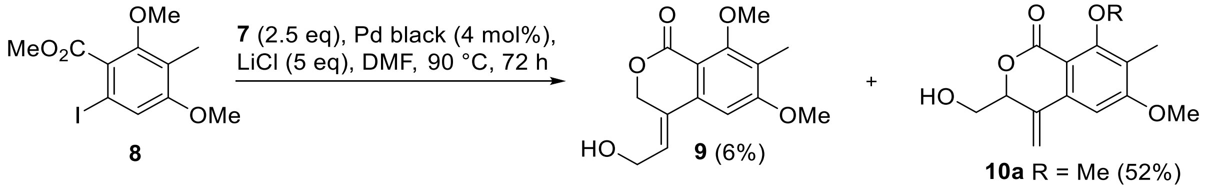

8

9 (6%)

10a R = Me (52%)
10b R = H (26%)

Pyranones 9, 10a and 10b: Lithium chloride (1.72 g; 40.45 mmol) was dried under reduced pressure (0.5–1 mbar) at 140 °C for 6 hours. Ester **8** (2.72 g; 8.09 mmol) and Pd black (35 mg; 0.324 mmol) were subsequently added under Ar atmosphere, followed by the solution of diol **7** (7.64 g; 20.23 mmol) in DMF (32 mL). The mixture was stirred at 90 °C in oil bath for 72 hours. The Pd black was then filtered off, washed by EtOAc and recycled. Afterwards, the mixture was diluted with EtOAc (50 mL) and washed with saturated NH_4Cl solution (80 mL), followed by 4% NaF solution (40 mL). The resultant suspension was filtered off, and the organic phase separated. The aqueous phase was extracted with EtOAc (3 × 30 mL). The combined organic

phases were dried over anhydrous Na_2SO_4, and concentrated under reduced pressure. The crude mixture was purified on silica gel (hexanes/EtOAc 7 : 3 → 1 : 1) to afford pyranones **9** (128 mg; 6%), **10a** (1.112 g; 52%) and **10b** (520 mg; 26%) as white amorphous solids.

(*Z*)-4-(2-Hydroxyethylidene)-6,8-dimethoxy-7-methylisochroman-1-one (**9**):

^{1}H NMR (600 MHz, $CDCl_3$) δ 6.69 (s, 1H), 6.30 (t, *J* = 6.6 Hz, 1H), 4.89 (s, 2H), 4.39 (d, *J* = 6.6 Hz, 2H), 3.88 (s, 3H), 3.81 (s, 3H), 2.58 (brs, 1H), 2.12 (s, 3H); **^{13}C NMR** (151 MHz, $CDCl_3$) δ 162.7, 161.9, 161.2, 138.9, 129.5, 128.5, 122.3, 109.4, 100.1, 65.1, 61.5, 58.6, 55.8, 8.7; **IR** (ATR) ν 3389, 2935, 1694, 1591, 1562, 1458, 1337, 1243, 1167, 1123, 1031, 1001 cm^{-1}; **HRMS** (TOF-ESI) *m*/*z*: $[M+H]^+$ Calcd. for $C_{14}H_{17}O_5^+$ 265.1071, Found 265.1084; **R*f*** 0.33 (hexanes/EtOAc 1 : 9).

3-(Hydroxymethyl)-6,8-dimethoxy-7-methyl-4-methyleneisochroman-1-one (**10a**):

^{1}H NMR (600 MHz, $CDCl_3$) δ 6.76 (s, 1H), 5.75 (s, 1H), 5.38 (s, 1H), 4.98–4.96 (m, 1H), 3.96–3.89 (m, 1H), 3.91 (s, 3H, overlap), 3.86–3.78 (m, 1H), 3.82 (s, 3H, overlap), 2.35 (dd, *J* = 7.7 Hz, *J* = 5.7 Hz, 1H), 2.14 (s, 3H); **^{13}C NMR** (151 MHz, $CDCl_3$) δ 162.8, 161.2, 160.8, 137.2, 136.8, 123.0, 113.8, 109.4, 100.7, 80.4, 63.7, 61.5, 55.8, 8.8; **IR** (ATR) ν 3359, 2937, 1684, 1587, 1333, 1245, 1144, 1123 cm^{-1}; **HRMS** (TOF-ESI) *m*/*z*: $[M+H]^+$ Calcd. for $C_{14}H_{17}O_5^+$ 265.1071, Found 265.1083; **R*f*** 0.51 (hexanes/EtOAc 1 : 9).

8-Hydroxy-3-(hydroxymethyl)-6-methoxy-7-methyl-4-methyleneisochroman-1-one (**10b**):

^{1}H NMR (500 MHz, $CDCl_3$) δ 11.36 (s, 1H), 6.59 (s, 1H), 5.78 (s, 1H), 5.36 (s, 1H), 5.09 (dd, *J* = 6.4 Hz, *J* = 4.7 Hz, 1H), 3.95–3.89 (m, 1H), 3.90 (s, 3H, overlap), 3.79 (dd, *J* = 12.2 Hz, *J* = 4.7 Hz, 1H), 2.26 (brs, 1H), 2.08 (s, 3H); **^{13}C NMR** (126 MHz, $CDCl_3$) δ 168.4, 163.6, 160.8, 135.4, 134.8, 114.7, 114.3, 100.2, 96.9, 82.2, 64.6, 55.8, 7.9; **IR** (ATR) ν 3469, 2924, 1647, 1364, 1285, 1231, 1169, 1133, 1062 cm^{-1}; **HRMS** (TOF-ESI) *m*/*z*: $[M+H]^+$ Calcd. for $C_{13}H_{15}O_5^+$ 251.0914, Found 251.0925; **R*f*** 0.66 (hexanes/EtOAc 1 : 9).

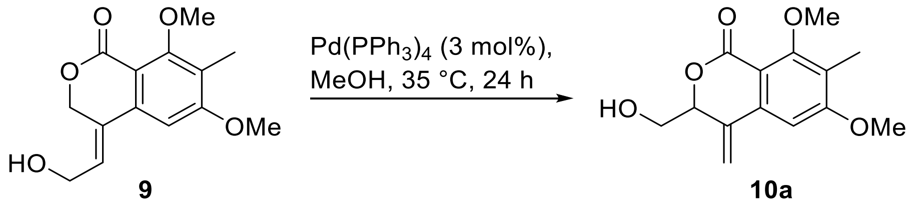

9 **10a**

3-(Hydroxymethyl)-6,8-dimethoxy-7-methyl-4-methyleneisochroman-1-one (10a): Pyranone **9** (80 mg; 0.30 mmol) and $Pd(PPh_3)_4$ (10.5 mg; 0.009 mmol) were dissolved in MeOH (1.5 mL) under Ar. The reaction was stirred at 35 °C in oil bath for 24 hours. The mixture was filtered through a pad of Celite®, and the pad was washed with EtOAc. The solvent was evaporated under reduced pressure, and the crude product purified on silica gel (hexanes/EtOAc 7 : 3) to afford isomer **10a** (75 mg; 94%) as a white amorphous solid.

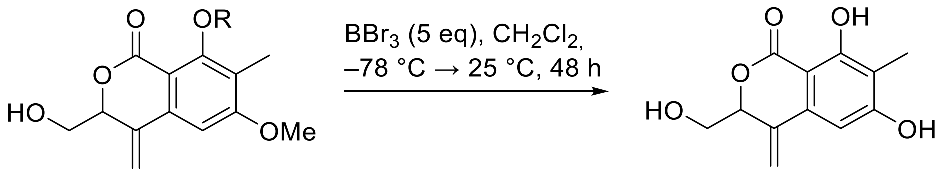

6,8-Dihydroxy-3-(hydroxymethyl)-7-methyl-4-methyleneisochroman-1-one (11): The mixture of pyranone **10a** (600 mg; 2.27 mmol) and 8-*O*-demethylated pyranone **10b** (300 mg; 1.2 mmol) was dissolved in CH_2Cl_2 (100 mL) under Ar atmosphere. The solution was cooled to –78 °C and BBr_3 (1M in CH_2Cl_2; 17.35 mL) was added dropwise. The cooling was turned off and the reaction mixture allowed to slowly warm to room temperature in the bath. After 48 hours, the mixture was poured into ice-cooled water (200 mL) and extracted with EtOAc (3 × 60 mL). The combined organic phases were dried over anhydrous Na_2SO_4, and concentrated under reduced pressure. The crude product was purified on silica gel (hexanes/EtOAc 7 : 3) to afford phenol **11** (607 mg; 74%) as a white amorphous solid. **^{1}H NMR** (500 MHz, CD_3OD) δ 6.62 (s, 1H), 5.76 (s, 1H), 5.37 (s, 1H), 5.05 (dd, *J* = 6.1 Hz, *J* = 5.1 Hz, 1H), 3.78 (dd, *J* = 12.0 Hz, *J* = 6.1 Hz, 1H), 3.71 (dd, *J* = 12.0 Hz, *J* = 5.0 Hz, 1H), 2.05 (s, 3H); **^{13}C NMR** (126 MHz, CD_3OD) δ 170.5, 163.9, 162.8, 137.3, 136.3, 114.6, 113.4, 102.6, 99.9, 84.0, 65.3, 7.9; **IR** (ATR) ν 3357, 3150, 2928, 1661, 1645, 1630, 1429, 1340, 1258, 1149, 1110, 1084, 1047 cm^{-1}; **HRMS** (TOF-ESI) *m*/*z*: $[M+H]^+$ Calcd. for $C_{12}H_{13}O_5^+$ 237.0757, Found 237.0768; **R*f*** 0.59 (hexanes/EtOAc 1 : 9).

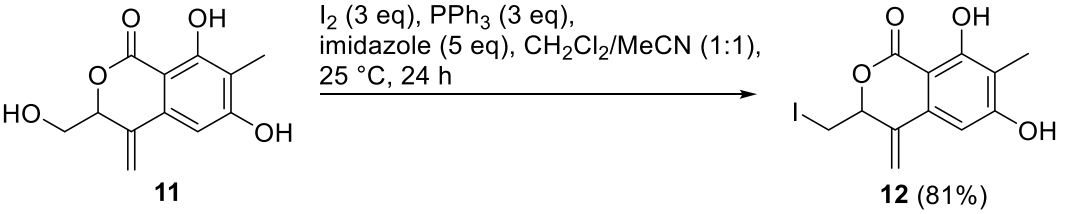

6,8-Dihydroxy-3-(iodomethyl)-7-methyl-4-methyleneisochroman-1-one (12): The mixture of PPh_3 (999 mg; 3.81 mmol) and imidazole (432 mg; 6.35 mmol) was dissolved in DCM (9 mL) under Ar atmosphere. The resulting solution was cooled to 0 °C and iodine (967 mg; 3.81 mmol) was added. The mixture was stirred at 0 °C for 30 minutes and the solution of alcohol **11** (300 mg; 1.27 mmol) in MeCN (9 mL) was added dropwise. The mixture was stirred at 25 °C for 24 hours. The reaction was diluted with EtOAc (20 mL) and washed with saturated $Na_2S_2O_3$ solution (50 mL). The aqueous phase was extracted with EtOAc (2 × 25 mL) and combined organic phases were dried over anhydrous Na_2SO_4. The solvent was evaporated and the crude product was purified on silica gel (hexanes/EtOAc 9 : 1) to afford iodide **12** (356 mg; 81%) as a yellowish amorphous solid. **^{1}H NMR** (600 MHz, acetonitrile-*D*$_3$) δ 11.50 (s, 1H), 7.82 (brs, 1H), 6.64 (s, 1H), 5.83 (s, 1H), 5.44 (d, *J* = 1.1 Hz, 1H), 5.20–5.17 (m, 1H), 3.53 (dd, *J* = 10.9 Hz, *J* = 7.3 Hz, 1H), 3.45 (dd, *J* = 10.9 Hz, *J* = 6.0 Hz, 1H), 2.04 (s, 3H); **^{13}C NMR** (151 MHz, acetonitrile-*D*$_3$) δ 169.0, 162.9, 162.6, 136.9, 135.2, 116.7, 113.2, 103.1, 100.1, 81.7, 8.1, 7.1; **IR** (ATR) ν 3194, 2922, 2852, 1632, 1612, 1378, 1314, 1274, 1257, 1159, 1150, 1107 cm^{-1}; **HRMS** (TOF-ESI) *m*/*z*: $[M+H]^+$ Calcd. for $C_{12}H_{12}IO_4^+$ 346.9775, Found 346.9777; **R*f*** 0.66 (hexanes/EtOAc 6 : 4).

2.2 Synthesis of (8*R*)-Scytolide (13)

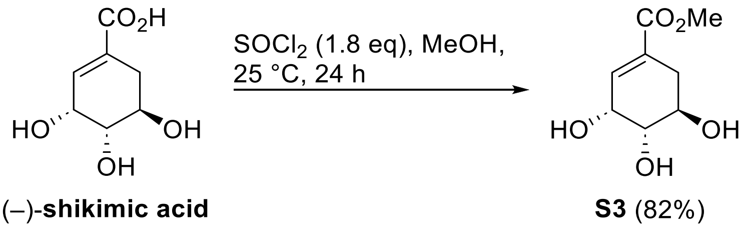

Methyl (–)-shikimate (S3): (–)-Shikimic acid (8.95 g; 51.39 mmol) was dissolved in MeOH (155 mL) and thionyl chloride (6.71 mL; 92.5 mmol) was then added dropwise. The mixture was stirred at 25 °C for 24 hours. The solvent was evaporated under reduced pressure and the crude product was recrystallized from boiling EtOAc to afford ester **S3** (7.94 g; 82%) as a white crystalline solid. All spectral data were in agreement with those reported in the literature[3]. **^{1}H NMR** (600 MHz, DMSO-d_6) δ 6.61 (d, *J* = 2.7 Hz, 1H), 4.86–4.80 (m, 2H), 4.63 (d, *J* = 4.3 Hz, 1H), 4.25–4.18 (m, 1H), 3.89–3.81 (m, 1H), 3.66 (s, 3H), 3.59–3.56 (m, 1H), 2.46–2.39 (m, 1H), 2.09–2.02 (m, 1H); **^{13}C NMR** (151 MHz, DMSO-d_6) δ 167.0, 140.0, 127.6, 70.2, 67.0, 65.6, 51.8, 29.8; **IR** (ATR) ν 3305, 2900, 1716, 1542, 1456, 1434, 1339, 1242, 1094, 1068 cm^{-1}; **HRMS** (TOF-ESI) *m/z*: $[M–H_2O+H]^+$ Calcd. for $C_8H_{11}O_4^+$ 171.0652, Found 171.0657; **m.p.** 114.5–115.0 °C (crystallization from boiling EtOAc); $[\alpha]_D^{25}$ –125.5 (*c* 0.73, EtOH); **R*f*** 0.13 (hexanes/EtOAc 1 : 9).

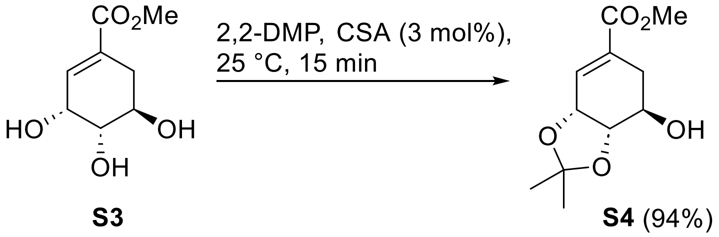

Methyl 5-*O*-acetyl-3,4-*O*-isopropylidene-(–)-shikimate (S4): Methyl (–)-shikimate **S3** (7.6 g; 40.39 mmol) and (1*S*)-(+)-10-camphorsulfonic acid (283 mg; 1.22 mmol) were dissolved in 2,2-dimethoxypropane (106 mL). The mixture was stirred at 25 °C for 15 minutes, followed by dilution with saturated solution of $NaHCO_3$ (70 mL). The mixture was extracted with Et_2O (5 × 50 mL). The combined organic phases were washed with saturated NaCl solution, dried over anhydrous Na_2SO_4, and concentrated under reduced pressure. The crude product was purified on silica gel (hexanes/EtOAc 6 : 4) to afford acetonide **S4** (8.67 g; 94%) as a colorless oil. All spectral data were in agreement with those reported in the literature[3]. **^{1}H NMR** (600 MHz, $CDCl_3$) δ 6.92–6.86 (m, 1H), 4.74–4.70 (m, 1H), 4.06 (dd, *J* = 7.6 Hz, *J* = 6.4 Hz, 1H), 3.91–3.85 (m, 1H), 3.74 (s, 3H), 2.80–2.74 (m, 1H), 2.34 (brs, 1H), 2.25–2.19 (m, 1H), 1.42 (s, 3H), 1.37 (s, 3H); **^{13}C NMR** (151 MHz, $CDCl_3$) δ 166.5, 133.9, 130.6, 109.7, 77.9, 72.2, 68.9, 52.1, 29.4, 28.0, 25.7; **IR** (ATR) ν 3447, 2987, 2917, 1716, 1437, 1381, 1372, 1241, 1215, 1163, 1104, 1051 cm^{-1}; **HRMS** (TOF-ESI) *m/z*: $[M+Na]^+$ Calcd. for $C_{11}H_{16}O_5Na^+$ 251.0890, Found 251.0889; $[\alpha]_D^{25}$ –76.1 (*c* 1.03, $CHCl_3$); **R*f*** 0.34 (hexanes/EtOAc 1 : 1).

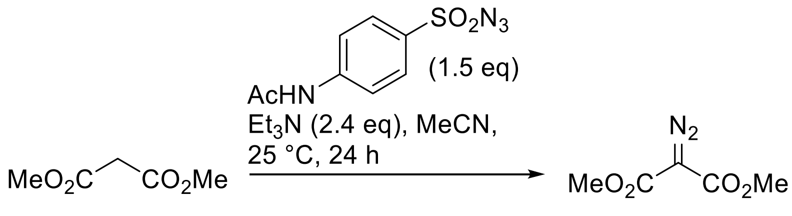

Dimethyl diazomalonate (S5): 4-Acetamidobenzenesulfonyl azide (25 g; 104.06 mmol) was dissolved in MeCN (300 mL) under Ar atmosphere. Then, Et_3N (23.3 mL; 167.28 mmol) and dimethyl malonate (8 mL; 69.7 mmol) were added and the mixture was stirred at 25 °C for 24 hours. The mixture obtained was filtered and the solvent was evaporated. The mixture was diluted with CH_2Cl_2 (300 mL) and filtered. The solvent was evaporated and the crude product was purified on silica gel (hexanes/EtOAc 8 : 2) to afford diazomalonate **S5** (10.25 g; 93%) as a yellow oil. All spectral data were in agreement with those reported in the literature[4]. **^{1}H NMR** (600 MHz, $CDCl_3$) δ 3.81 (s, 6H); **^{13}C NMR** (151 MHz, $CDCl_3$) δ 161.4, 52.5; **IR** (ATR) ν 2958, 2923, 2851, 2136, 1760, 1738, 1693, 1437, 1354, 1331, 1274, 1190, 1090 cm^{-1}; **HRMS** (TOF-ESI) *m*/*z*: $[M+H]^+$ Calcd. for $C_5H_7N_2O_4^+$ 159.0400, Found 159.0406; **R*f*** 0.25 (hexanes/EtOAc 8 : 2).

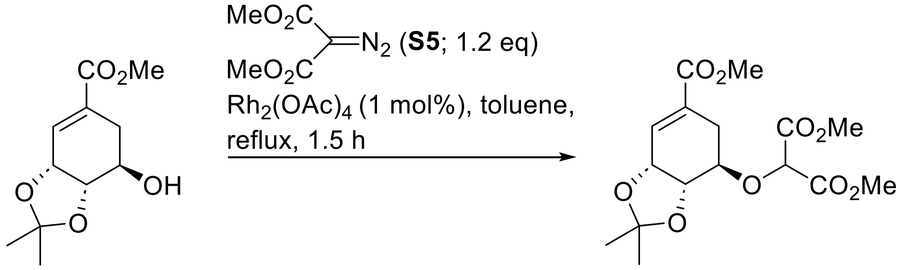

Dimethyl 2-(((3a*S*,4*R*,7a*R*)-6-(methoxycarbonyl)-2,2-dimethyl-3a,4,5,7a-tetrahydrobenzo[*d*][1,3]dioxol-4-yl)oxy)malonate (S6): The mixture of acetonide **S4** (8.5 g; 37.24 mmol) and freshly prepared dimethyl diazomalonate **S5** (7.07 g; 44.69 mmol) was dissolved in anhydrous toluene (223 mL). Rhodium(II) acetate (165 mg; 0.372 mmol) was added and the mixture was stirred under reflux in oil bath for 1.5 hours. The solvent was evaporated under reduced pressure, and the crude product was purified on silica gel (hexanes/EtOAc 8 : 2 → 6 : 4) to afford malonate **S6** (11.3 g; 85%) as a colorless oil. All spectral data were in agreement with those reported in the literature[3,5]. **^{1}H NMR** (500 MHz, $CDCl_3$) δ 6.89–6.86 (m, 1H), 4.91 (s, 1H), 4.74–4.71 (m, 1H), 4.25 (dd, *J* = 7.4 Hz, *J* = 6.5 Hz, 1H), 3.78 (s, 3H), 3.76 (s, 3H), 3.74 (s, 3H), 3.72–3.67 (m, 1H), 2.87–2.80 (m, 1H), 2.39–2.31 (m, 1H), 1.40 (s, 3H), 1.34 (s, 3H); **^{13}C NMR** (126 MHz, $CDCl_3$) δ 167.0, 166.9, 166.2, 133.9, 130.2, 109.7, 78.4, 77.5, 76.8, 72.5, 52.9, 52.8, 52.1, 27.7, 27.4, 25.6; **IR** (ATR) ν 2989, 2955, 2928, 2854, 1767, 1747, 1717, 1437, 1287, 1245, 1215, 1163, 1132, 1057, 1031 cm^{-1}; **HRMS** (TOF-ESI) *m*/*z*: $[M+Na]^+$ Calcd. for $C_{16}H_{22}O_9Na^+$ 381.1156, Found 381.1157; $[\alpha]_D^{25}$ –45.6 (*c* 1.15, $CHCl_3$); **R*f*** 0.35 (hexanes/EtOAc 1 : 1).

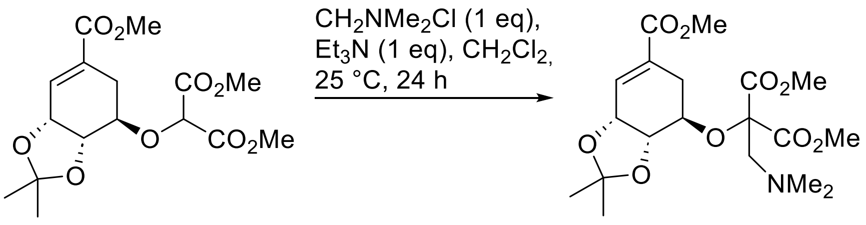

S6 **S7** (99%)

Dimethyl 2-((dimethylamino)methyl)-2-(((3a*S*,4*R*,7a*R*)-6-(methoxycarbonyl)-2,2-dimethyl-3a,4,5,7a-tetrahydrobenzo[*d*][1,3]dioxol-4-yl)oxy)malonate (S7): The malonate **S6** (11.25 g; 31.4 mmol) was dissolved in dry CH_2Cl_2 (205 mL), followed by addition of Et_3N (4.377 mL; 31.4 mmol) and Eschenmoser´s salt (3.525 g; 37.68 mmol). The mixture was stirred at 25 °C for 24 hours. Then, distilled water (100 mL) was added and the mixture was extracted with CH_2Cl_2 (3 × 50 mL). The combined organic phases were washed with saturated Na_2CO_3 solution, saturated NaCl solution, dried over anhydrous Na_2SO_4, and concentrated under reduced pressure to afford Eschenmoser adduct **S7** (12.9 g; 99%) as a colorless oil. All spectral data were in agreement with those reported in the literature[3,5]. **^{1}H NMR** (600 MHz, $CDCl_3$) δ 6.78–6.76 (m, 1H), 4.69–4.66 (m, 1H), 4.48–4.44 (m, 2H), 3.77 (s, 3H), 3.76 (s, 3H), 3.73 (s, 3H), 2.87 (d, *J* = 13.9 Hz, 1H), 2.80 (d, *J* = 13.9 Hz, 1H), 2.57–2.51 (m, 1H), 2.49–2.43 (m, 1H), 2.20 (s, 6H), 1.35 (s, 3H), 1.29 (s, 3H); **^{13}C NMR** (151 MHz, $CDCl_3$) δ 168.7, 168.4, 167.1, 135.0, 128.1, 109.4, 86.7, 74.0, 72.7, 71.9, 63.4, 52.5, 52.4, 51.9, 47.3, 27.9, 26.3, 26.2; **IR** (ATR) ν 2986, 2952, 2824, 2774, 1764, 1742, 1717, 1435, 1247, 1194, 1101, 1055, 1041 cm^{-1}; **HRMS** (TOF-ESI) *m*/*z*: $[M+H]^+$ Calcd. for $C_{19}H_{30}NO_9^+$ 416.1915, Found 416.1917; $[\alpha]_D^{25}$ +7.5 (*c* 0.8, $CHCl_3$); **R*f*** 0.48 (hexanes/EtOAc 1 : 1).

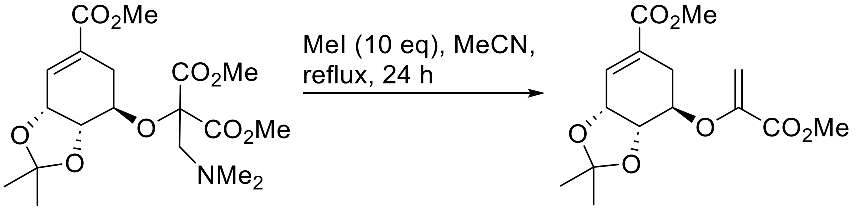

S7 **S8** (99%)

Methyl (3a*R*,7*R*,7a*S*)-7-((3-methoxy-3-oxoprop-1-en-2-yl)oxy)-2,2-dimethyl-3a,6,7,7a-tetrahydrobenzo[*d*][1,3]dioxole-5-carboxylate (S8): Eschenmoser adduct **S7** (12.14 g; 29.22 mmol) was dissolved in MeCN (104 mL) and MeI (18.2 mL; 292.2 mmol) was added. The mixture was stirred under reflux in oil bath for 24 hours. Et_2O (100 mL) was then added and the solid precipitate was filtered off. The solution was dried over anhydrous Na_2SO_4 and the solvent was evaporated to afford crude elimination product **S8** (9 g; 99%) as a colorless oil. All spectral data were in agreement with those reported in the literature[3,5]. **^{1}H NMR** (600 MHz, $CDCl_3$) δ 6.89–6.88 (m, 1H), 5.49 (d, *J* = 2.7 Hz, 1H), 4.80–4.77 (m, 2H), 4.39–4.34 (m, 2H), 3.75 (s, 3H), 3.74 (s, 3H), 2.78–2.72 (m, 1H), 2.49–2.42 (m, 1H), 1.38 (s, 3H), 1.37 (s, 3H); **^{13}C NMR** (151 MHz, $CDCl_3$) δ 166.5, 163.5, 149.7, 134.8, 128.7, 109.8, 97.7, 74.3, 74.2, 72.0, 52.4, 52.1, 27.8, 25.8, 24.8; **IR** (ATR) ν 2987, 2950, 1734, 1720, 1621, 1340, 1324, 1259, 1221, 1205, 1167, 1069, 1034 cm^{-1}; **HRMS** (TOF-ESI) *m*/*z*: $[M+Na]^+$ Calcd. for $C_{15}H_{20}O_7Na^+$ 335.1101, Found 335.1103; $[\alpha]_D^{25}$ –78.6 (*c* 0.83, $CHCl_3$); **R*f*** 0.53 (hexanes/EtOAc 1 : 1).

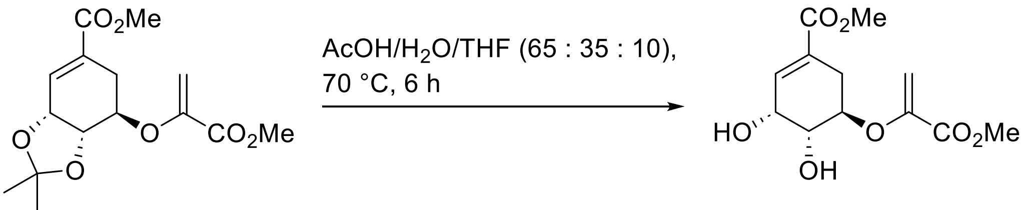

S8 **S9** (54%)

Methyl (3*R*,4*R*,5*R*)-3,4-dihydroxy-5-((3-methoxy-3-oxoprop-1-en-2-yl)oxy)cyclohex-1-ene-1-carboxylate (S9): The elimination product **S8** (10.94 g; 35.03 mmol) was dissolved in a mixture of AcOH/H_2O/THF (65 : 35 : 10; 75 mL). The solution was stirred at 70 °C in oil bath for 6 hours and then diluted with CH_2Cl_2 (60 mL). The mixture was washed with saturated Na_2CO_3 solution (50 mL) and saturated NaCl solution (50 mL). The organic phase was dried over anhydrous Na_2SO_4, the solvent was evaporated under reduced pressure, and the crude product was purified on silica gel (hexanes/EtOAc 8 : 2) to afford diol **S9** (5.14 g; 54%) as a colorless oil. All spectral data were in agreement with those reported in the literature[3,5]. **^{1}H NMR** (600 MHz, $CDCl_3$) δ 6.90–6.87 (m, 1H), 5.55 (d, *J* = 2.7 Hz, 1H), 4.93 (d, *J* = 2.7 Hz, 1H), 4.53 (t, *J* = 4.3 Hz, 1H), 4.29 (td, *J* = 8.2 Hz, *J* = 5.4 Hz, 1H), 3.93 (dd, *J* = 8.8 Hz, *J* = 4.3 Hz, 1H), 3.79 (s, 3H), 3.74 (s, 3H), 3.65 (brs, 1H), 3.00 (ddt, *J* = 18.0 Hz, *J* = 5.4 Hz, *J* = 1.2 Hz, 1H), 2.89 (brs, 1H), 2.37–2.28 (m, 1H); **^{13}C NMR** (151 MHz, $CDCl_3$) δ 166.5, 164.1, 149.7, 135.9, 129.9, 99.7, 75.1, 70.4, 65.9, 52.6, 52.1, 28.2; **IR** (ATR) ν 3421, 2954, 2926, 2854, 1716, 1622, 1439, 1250, 1203, 1171, 1103, 1048 cm^{-1}; **HRMS** (TOF-ESI) *m*/*z*: $[M+Na]^+$ Calcd. for $C_{12}H_{16}O_7Na^+$ 295.0788, Found 295.0788; $[\alpha]_D^{25}$ –161.1 (*c* 0.71, $CHCl_3$); **R*f*** 0.31 (hexanes/EtOAc 1:1).

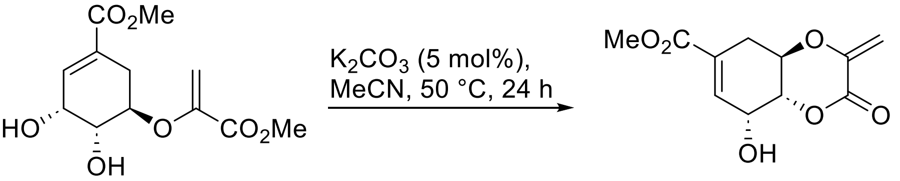

S9 (8*R*)-scytolide (**13**; 90%)

(8*R*)-scytolide (13): Diol **S9** (700 mg; 2.57 mmol) and K_2CO_3 (18 mg; 0,129 mmol) were dissolved in MeCN (39 mL) and the mixture was stirred at 50 °C in oil bath for 24 hours. The reaction was quenched by addition of saturated solution of NH_4Cl (20 mL) and the mixture was extracted with CH_2Cl_2 (3 × 20 mL). The combined organic phases were dried over anhydrous Na_2SO_4 and the solvent was evaporated to afford crude (8*R*)-scytolide (**13**; 555 mg; 90%) as a white amorphous solid. All spectral data were in agreement with those reported in the literature[3,5]. **^{1}H NMR** (500 MHz, DMSO-d_6) δ 6.79 (dd, *J* = 5.6 Hz, *J* = 2.6 Hz, 1H), 5.78 (brs, 1H), 5.48 (d, *J* = 1.4 Hz, 1H), 5.08 (d, *J* = 1.4 Hz, 1H), 4.62 (dd, *J* = 10.6 Hz, *J* = 4.6 Hz, 1H), 4.44 (t, *J* = 4.6 Hz, 1H), 4.36–4.29 (m, 1H), 3.71 (s, 3H), 2.97 (dd, *J* = 17.3 Hz, *J* = 6.4 Hz, 1H), 2.30 (ddd, *J* = 17.3 Hz, *J* = 9.5 Hz, *J* = 2.9 Hz, 1H); **^{13}C NMR** (126 MHz, DMSO-d_6) δ 166.0, 159.5, 146.8, 136.3, 128.5, 103.2, 78.7, 68.0, 62.8, 52.3, 30.0; **IR** (ATR) ν 3412, 2953, 2924, 1713, 1705, 1626, 1314, 1252, 1168, 1102, 1077, 1065 cm^{-1}; **HRMS** (TOF-ESI) *m*/*z*: $[M+H]^+$ Calcd. for $C_{11}H_{13}O_6^+$ 241.0707, Found 241.0709; $[\alpha]_D^{25}$ –99.1 (*c* 0.21, $CHCl_3$); **R*f*** 0.49 (hexanes/EtOAc 1:1). The compound is not stable due to spontaneous polymerization. However, in the form of powder, no polymerization was observed.

2.3 Synthesis of Scytolide (6)

2.3.1 Mitsunobu Inversion of (8*R*)-Scytolide (13)

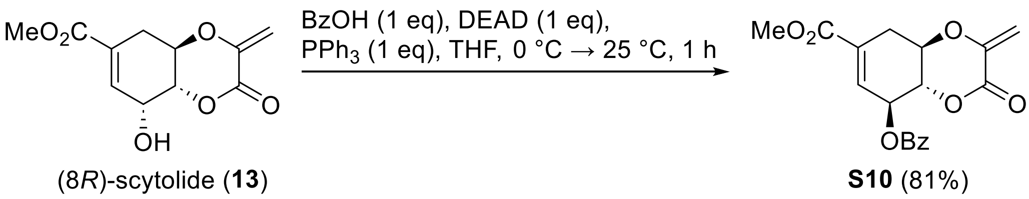

Methyl (4a*R*,8*S*,8a*S*)-8-(benzoyloxy)-3-methylene-2-oxo-2,3,4a,5,8,8a-hexahydrobenzo[*b*][1,4]dioxine-6-carboxylate (S10): The mixture of (8*R*)-scytolide (**13**; 1 g; 4.16 mmol), benzoic acid (508 mg; 4.16 mmol) and PPh_3 (1.091 g; 4.16 mmol) was dissolved in dry THF (29 mL). The resulting solution was cooled to 0 °C and DEAD (40% solution in toluene; 1.632 mL; 4.16 mmol) was added dropwise. The mixture was stirred at 25 °C for 1 hour, then diluted with EtOAc (20 mL) and washed with saturated NaCl solution (30 mL). The aqueous phase was extracted with EtOAc (2 × 20 mL). The combined organic phases were dried over anhydrous Na_2SO_4. The solvent was evaporated and the crude product was purified on silica gel (hexanes/EtOAc 95 : 5 → 9 : 1) to afford benzoate **S10** (1.16 g; 81%) as a white amorphous solid. **^{1}H NMR** (500 MHz, $CDCl_3$) δ 8.09–8.05 (m, 2H), 7.64–7.59 (m, 1H), 7.49–7.45 (m, 2H), 6.82 (t, *J* = 2.7 Hz, 1H), 5.97–5.92 (m, 1H), 5.73 (d, *J* = 1.2 Hz, 1H), 5.16 (d, *J* = 1.2 Hz, 1H), 4.81 (dd, *J* = 10.3 Hz, *J* = 8.3 Hz, 1H), 4.30–4.23 (m, 1H), 3.80 (s, 3H), 3.19 (dd, *J* = 17.8 Hz, *J* = 6.4 Hz, 1H), 2.53 (ddt, *J* = 17.3 Hz, *J* = 10.0 Hz, *J* = 3.4 Hz, 1H); **^{13}C NMR** (126 MHz, $CDCl_3$) δ 165.6, 165.2, 158.6, 145.9, 133.8, 133.6, 129.9, 129.3, 129.0, 128.5, 105.2, 78.8, 71.7, 70.3, 52.5, 29.3; **IR** (ATR) ν 2954, 1721, 1452, 1436, 1313, 1246, 1177, 1137, 1094, 1070, 1026 cm^{-1}; **HRMS** (TOF-ESI) *m*/*z*: $[M+H]^+$ Calcd. for $C_{18}H_{17}O_7^+$ 345.0969, Found 345.0972; $[\alpha]_D^{25}$ +86.4 (*c* 0.25, DMSO); **R_f** 0.53 (hexanes/EtOAc 7 : 3). The compound is not stable due to spontaneous polymerization. However, in the form of powder, no polymerization was observed.

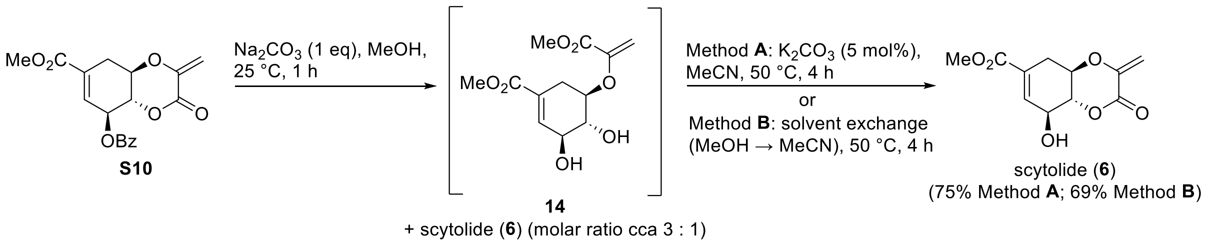

Scytolide (6):

Method A: Benzoate **S10** (500 mg; 1.45 mmol) and Na_2CO_3 (154 mg; 1.45 mmol) were suspended in MeOH (26 mL) and the mixture was stirred at 25 °C for 1 hour. The mixture was filtered through a pad of silica gel, washed with MeOH (50 mL), and the solvent was evaporated. The resulting crude mixture of methyl ester **14** and scytolide (**6**) (molar ratio cca 3 : 1, determined by ^{1}H NMR) was dried under reduced pressure (0.5–1 mbar) for 30 minutes, and then dissolved in MeCN (22 mL). After addition of K_2CO_3 (10 mg; 0.073 mmol), the mixture was stirred at 50 °C in oil bath for 4 hours until complete conversion of methyl ester **14** to scytolide (**6**). The mixture was diluted with EtOAc (20 mL) and washed with saturated NH_4Cl solution (30 mL). The aqueous phase was extracted with EtOAc (2 × 20 mL) and

combined organic phases were dried over anhydrous Na_2SO_4. The solvent was evaporated and the crude product was purified on silica gel (hexanes/EtOAc 75 : 25) to afford scytolide (**6**; 261 mg; 75%) as a white amorphous solid. All spectral data were in agreement with those reported in the literature[3].

Method **B**: Benzoate **S10** (450 mg; 1.31 mmol) and Na_2CO_3 (139 mg; 1.31 mmol) were suspended in MeOH (23 mL) and the mixture was stirred at 25 °C for 1 hour. The mixture was diluted with MeCN (100 mL) and the solvent was evaporated. The resulting crude mixture of methyl ester **14** and scytolide (**6**) was dissolved in MeCN (20 mL) and stirred at 50 °C in oil bath for 4 hours until complete conversion of methyl ester **14** to scytolide (**6**). The mixture was diluted with EtOAc (20 mL) and washed with saturated NH_4Cl solution (30 mL). The aqueous phase was extracted with EtOAc (2 × 20 mL) and combined organic phases were dried over anhydrous Na_2SO_4. The solvent was evaporated and the crude product was purified on silica gel (hexanes/EtOAc 75 : 25) to afford scytolide (**6**; 218 mg; 69%) as a white amorphous solid. All spectral data were in agreement with those reported in the literature[3]. **^{1}H NMR** (500 MHz, DMSO-d_6) δ 6.57 (t, *J* = 2.3 Hz, 1H), 5.97 (d, *J* = 5.4 Hz, 1H), 5.45 (d, *J* = 1.5 Hz, 1H), 5.06 (d, *J* = 1.5 Hz, 1H), 4.48–4.42 (m, 2H), 4.39–4.32 (m, 1H), 3.71 (s, 3H), 2.87 (dd, *J* = 17.0 Hz, *J* = 6.3 Hz, 1H), 2.33 (ddt, *J* = 16.4 Hz, *J* = 9.7 Hz, *J* = 3.1 Hz, 1H); **^{1}H NMR** (600 MHz, $CDCl_3$) δ 6.77–6.75 (m, 1H), 5.68 (d, *J* = 1.6 Hz, 1H), 5.10 (d, *J* = 1.6 Hz, 1H), 4.61–4.56 (m, 1H), 4.39 (dd, *J* = 10.2 Hz, *J* = 8.0 Hz, 1H), 4.09 (td, *J* = 10.2 Hz, *J* = 6.5 Hz, 1H), 3.78 (s, 3H), 3.09–3.03 (m, 1H), 2.75–2.70 (m, 1H), 2.48–2.39 (m, 1H); **^{13}C NMR** (151 MHz, DMSO-d_6) δ 165.6, 159.2, 146.8, 139.6, 126.3, 102.9, 82.0, 69.9, 68.6, 52.3, 29.1; **^{13}C NMR** (151 MHz, $CDCl_3$) δ 165.6, 159.0, 146.1, 136.9, 127.9, 105.1, 82.3, 70.1 (2 × C), 52.4, 29.6; **IR** (ATR) ν 3427, 2953, 2920, 2865, 1723, 1708, 1654, 1627, 1325, 1306, 1251, 1085, 1062, 1037 cm^{-1}; **HRMS** (TOF-ESI) *m*/*z*: $[M+H]^+$ Calcd. for $C_{11}H_{13}O_6^+$ 241.0707, Found 241.0710; **m.p.** 195.6 °C (decomposition to brown solid; crystallization from EtOAc/hexanes at 25 °C for 4 days); $[\alpha]_D^{25}$ +40.0 (*c* 0.50, MeCN), +38.0 (*c* 0.11, $CHCl_3$), +38.5 (*c* 0.26, MeOH); **ECD** (*c* 0.017, MeOH) λ_{max} (Δε) 220 (+4.63), 242 (−8.74); **R*f*** 0.49 (hexanes/EtOAc 1 : 1). The compound is not stable due to spontaneous polymerization. However, in the form of powder, no polymerization was observed.

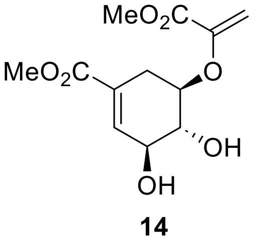

14

Methyl (3*S*,4*R*,5*R*)-3,4-dihydroxy-5-((3-methoxy-3-oxoprop-1-en-2-yl)oxy)cyclohex-1-ene-1-carboxylate (14): White amorphous solid; **^{1}H NMR** (500 MHz, $CDCl_3$) δ 6.82–6.79 (m, 1H), 5.54 (d, *J* = 2.8 Hz, 1H), 4.88 (d, *J* = 2.8 Hz, 1H), 4.37–4.31 (m, 1H), 4.12 (td, *J* = 9.0 Hz, *J* = 5.7 Hz, 1H), 3.90 (dd, *J* = 9.0 Hz, *J* = 7.2 Hz, 1H), 3.79 (s, 3H), 3.74 (s, 3H), 2.98 (ddt, *J* = 17.8 Hz, *J* = 5.7 Hz, *J* = 1.4 Hz, 1H), 2.69 (brs, 2H), 2.42–2.34 (m, 1H); **^{13}C NMR** (126 MHz, $CDCl_3$) δ 166.3, 164.0, 149.5, 138.0, 127.3, 99.1, 76.9, 74.3, 71.3, 52.7, 52.1, 28.4; **IR** (ATR) ν 3396, 3344, 2945, 2891, 2364, 1716, 1618, 1439, 1246, 1229, 1209, 1174, 1102, 1029 cm^{-1}; **HRMS** (TOF-ESI) *m*/*z*: $[M+Na]^+$ Calcd. for $C_{12}H_{16}O_7Na^+$ 295.0788,

Found 295.0792; $[\alpha]_D^{25}$ −112.0 (*c* 0.125, $CHCl_3$); **R*f*** 0.40 (hexanes/EtOAc 1 : 9). The compound in not stable in solution due to spontaneous lactonization to scytolide (**6**).

2.3.2 Oxidation-Reduction Method

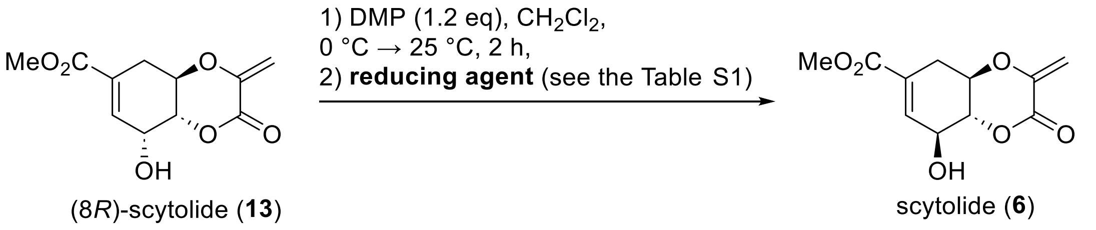

Table S1. Optimization of reaction conditions.

	Reducing Agent	Reduction Conditions	Yield (%)	Scytolide (**6**)/ (8*R*)-Scytolide (**13**) Ratio[a)]
1	$NaBH_4$ (1.1 eq)/ $CeCl_3 \cdot 7H_2O$ (1.5 eq)	0 °C, 4 h	70	3 : 2
2[b)]	$NaBH(OAc)_3$ (4 eq)	0 °C → 25 °C, 4 h	52	2 : 1
3	$BH_3 \cdot THF$ (1.1 eq)	0 °C, 6 h 15 min	70	only (8*R*)-scytolide (**13**)
4	K-Selectride (4 eq)	0 °C, 6 h 15 min	–	m/p[c)]

[a)] Determined by ^1H NMR. [b)] See the ref. *3.* [c)] m/p = mixture of products.

General procedure: (8*R*)-Scytolide (**13**; 70 mg; 0.29 mmol; 1 eq) was dissolved in CH_2Cl_2 (4.3 mL; 14.8 mL/mmol) under Ar atmosphere and the solution was cooled to 0 °C. Dess-Martin periodinane (148 mg; 0.35 mmol; 1.2 eq) was added and the mixture was stirred at 25 °C for 2 hours. The resulting suspension was cooled to 0 °C and reducing agent (1.1–4 eq; see the Table S1) was added. The mixture was stirred at 0 °C (or 25 °C) for the period given in the Table S1 before addition of saturated $NaHCO_3$ solution (5 mL). The mixture was extracted with EtOAc (10 mL), the organic phase was washed with saturated NaCl solution (10 mL) and dried over anhydrous Na_2SO_4. The solvent was evaporated and the crude product was purified on silica gel (hexanes/EtOAc 75 : 25) to afford inseparable mixture of scytolide (**6**) and (8*R*)-scytolide (**13**) as a white amorphous solid. All spectral data were in agreement with those reported in the literature[3].

2.4 Diels-Alder Reactions

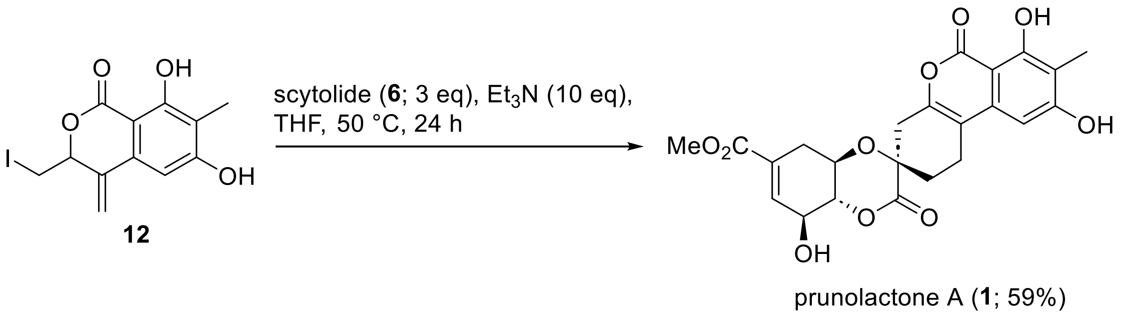

Prunolactone A (1): The mixture of iodide **12** (100 mg; 0.29 mmol) and scytolide (**6**; 208 mg; 0.87 mmol) was dissolved in THF (11 mL) under Ar atmosphere, followed by addition of Et_3N (403 μL; 2.9 mmol). The resulting solution was stirred at 50 °C in oil bath for 24 hours. The mixture was diluted with EtOAc (10 mL) and washed with saturated NH_4Cl solution (15 mL). The aqueous phase was extracted with EtOAc (2 × 15 mL) and combined organic phases were dried over anhydrous Na_2SO_4. The solvent was evaporated and the crude product was purified on silica gel (hexanes/EtOAc 75 : 25 → 1 : 1) to afford prunolactone A (**1**; 78 mg; 59%) and recovered scytolide (**6**; 138 mg; 66%) as white amorphous solids. All spectral data were in agreement with those reported in the literature[6]. **^{1}H NMR** (600 MHz, acetone-d_6) δ 11.63 (s, 1H), 9.54 (brs, 1H), 6.66 (t, J = 2.3 Hz, 1H), 6.51 (s, 1H), 5.03 (brs, 1H), 4.59–4.54 (m, 1H), 4.37 (dd, J = 9.9 Hz, J = 8.0 Hz, 1H), 4.27 (td, J = 9.9 Hz, J = 6.2 Hz, 1H), 3.69 (s, 3H), 3.05 (s, 2H), 2.81 (dd, J = 17.5 Hz, J = 6.2 Hz, 1H), 2.67–2.63 (m, 2H), 2.32–2.28 (m, 1H), 2.27–2.22 (m, 1H), 2.22–2.17 (m, 1H), 2.11 (s, 3H); **^{1}H NMR** (600 MHz, DMSO-d_6) δ 11.47 (s, 1H), 10.92 (brs, 1H), 6.55 (t, J = 2.5 Hz, 1H), 6.45 (s, 1H), 5.88 (brs, 1H), 4.45–4.40 (m, 1H), 4.32 (dd, J = 9.9 Hz, J = 8.0 Hz, 1H), 4.22 (td, J = 9.9 Hz, J = 6.2 Hz, 1H), 3.66 (s, 3H), 3.08 (d, J = 18.1 Hz, 1H), 2.98 (d, J = 18.1 Hz, 1H), 2.68 (dd, J = 16.4 Hz, J = 6.6 Hz, 1H), 2.61–2.54 (m, 1H), 2.53–2.46 (m, 1H), 2.27–2.21 (m, 1H), 2.18–2.05 (m, 2H), 2.02 (s, 3H); **^{13}C NMR** (151 MHz, acetone-d_6) δ 171.1, 166.9, 166.4, 164.2, 162.3, 148.4, 139.7, 137.3, 128.3, 111.1, 109.9, 99.6 (2 × C), 85.3, 77.5, 70.4, 67.1, 52.2, 34.9, 32.3, 30.5, 19.6, 8.0; **^{13}C NMR** (151 MHz, DMSO-d_6) δ 170.7, 165.8, 165.7, 164.3, 160.7, 147.5, 139.8, 136.1, 126.7, 109.9, 108.9, 99.1, 97.8, 84.1, 76.2, 68.8, 65.8, 52.2, 34.0, 31.1, 29.6, 18.7, 8.1; **IR** (ATR) ν 3390, 1720, 1682, 1625, 1436, 1377, 1255, 1109, 1070, 1053 cm^{-1}; **HRMS** (TOF-ESI) *m/z*: $[M+H]^+$ Calcd. for $C_{23}H_{23}O_{10}^+$ 459.1286, Found 459.1286; $[\alpha]_D^{25}$ +53.6 (*c* 0.5, MeCN), +60.0 (*c* 0.1, MeCN); **R*f*** 0.30 (hexanes/EtOAc 3 : 7).

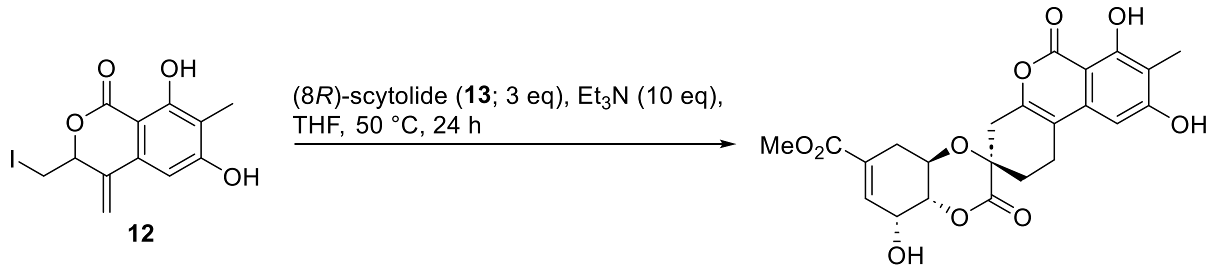

(3´*R*)-Prunolactone A (16): The mixture of iodide **12** (100 mg; 0.29 mmol) and (8*R*)-scytolide (**13**; 208 mg; 0.87 mmol) was dissolved in THF (11 mL) under Ar atmosphere, followed by addition of Et_3N (403 µL; 2.9 mmol). The resulting solution was stirred at 50 °C in oil bath for 24 hours. The mixture was diluted with EtOAc (10 mL) and washed with saturated NH_4Cl solution (15 mL). The aqueous phase was extracted with EtOAc (2 × 15 mL) and combined organic phases were dried over anhydrous Na_2SO_4. The solvent was evaporated and the crude product was purified on silica gel (hexanes/EtOAc 75 : 25 → 1 : 1) to afford (3´*R*)-prunolactone A (**16**; 81 mg; 61%) and recovered (8*R*)-scytolide (**13**; 112 mg; 54%) as white amorphous solids. **^{1}H NMR** (500 MHz, DMSO-*d*$_6$) δ 11.46 (s, 1H), 10.91 (brs, 1H), 6.75 (dd, *J* = 5.6 Hz, *J* = 2.6 Hz, 1H), 6.45 (s, 1H), 5.69 (d, *J* = 4.3 Hz, 1H), 4.48 (dd, *J* = 10.4 Hz, *J* = 4.1 Hz, 1H), 4.41–4.34 (m, 1H), 4.23 (td, *J* = 9.8 Hz, *J* = 6.2 Hz, 1H), 3.66 (s, 3H), 2.99 (s, 2H), 2.77 (dd, *J* = 17.4 Hz, *J* = 6.3 Hz, 1H), 2.62–2.55 (m, 1H), 2.55–2.50 (m, 1H), 2.26–2.19 (m, 1H), 2.14–2.05 (m, 2H), 2.02 (s, 3H); **^{13}C NMR** (126 MHz, DMSO-*d*$_6$) δ 171.0, 166.0, 165.8, 164.0, 160.7, 147.5, 136.4, 136.1, 128.9, 110.0, 109.0, 99.0, 97.9, 80.8, 76.2, 63.4, 63.0, 52.2, 33.7, 31.3, 30.3, 18.7, 8.1; **IR** (ATR) ν 3402, 2955, 1716, 1682, 1624, 1436, 1304, 1255, 1169, 1103, 1082 cm^{-1}; **HRMS** (TOF-ESI) *m*/*z*: $[M+H]^+$ Calcd. for $C_{23}H_{23}O_{10}^+$ 459.1286, Found 459.1287; $[\alpha]_D^{25}$ –22.4 (c 0.25, DMSO); **R*f*** 0.27 (hexanes/EtOAc 3 : 7).

3 ^{1}H and ^{13}C NMR Spectra of the Compounds

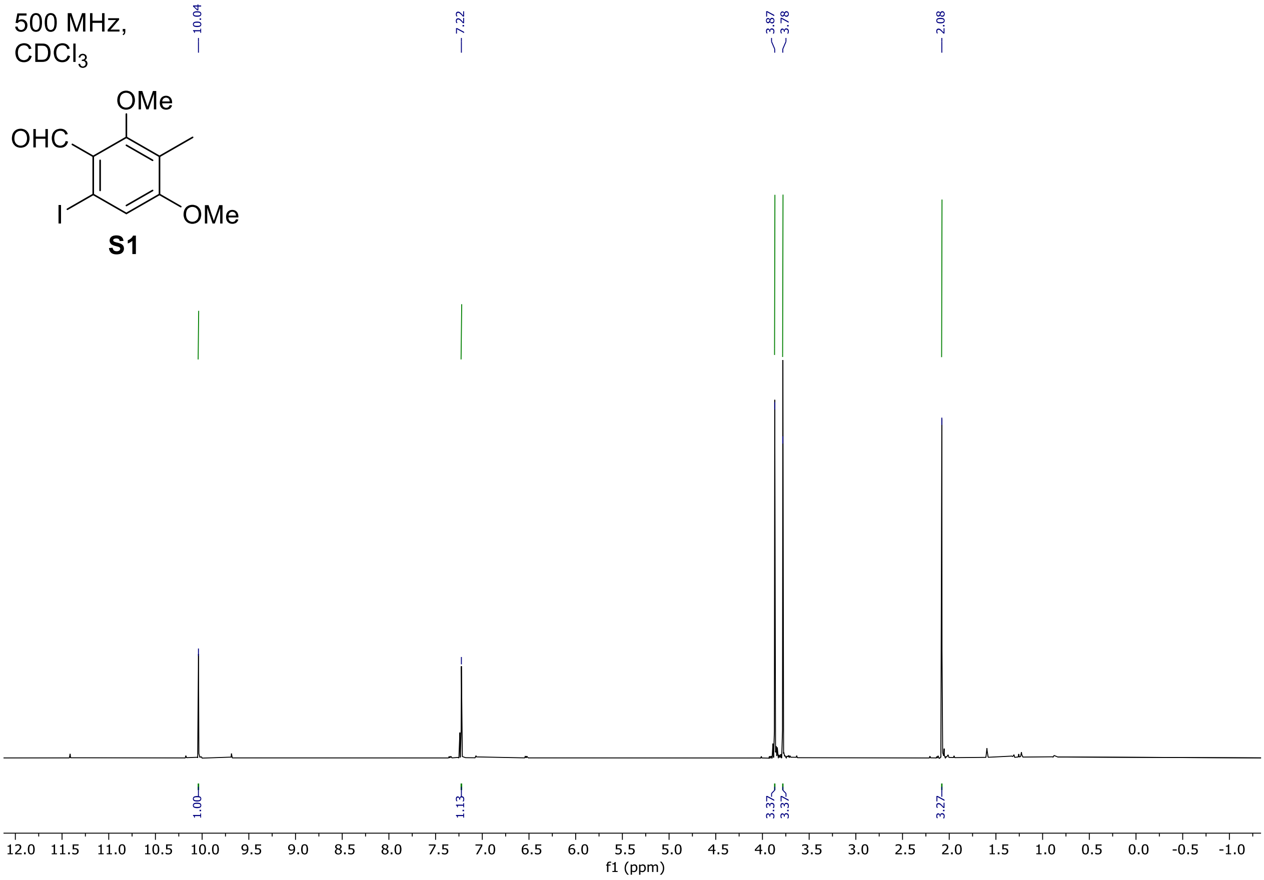

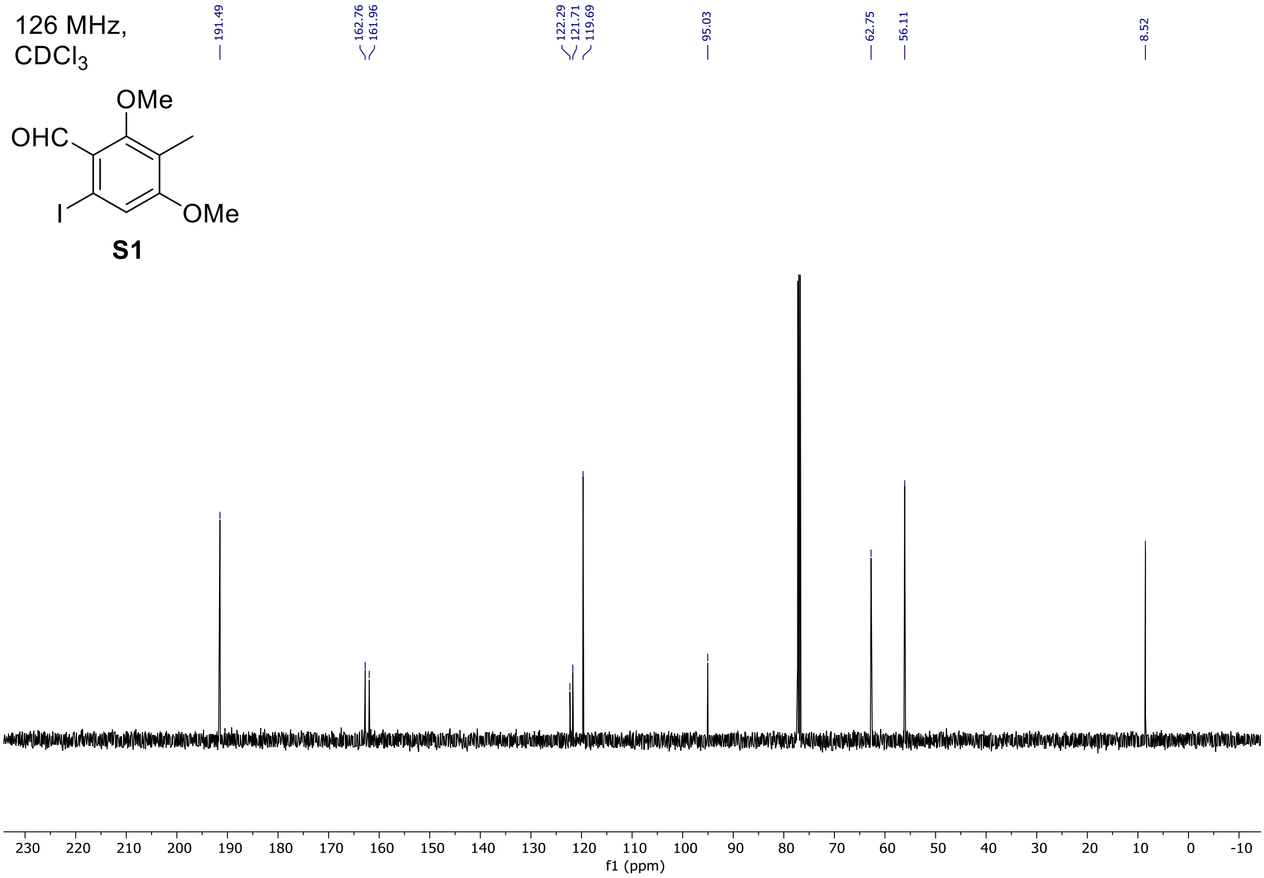

500 MHz,
$CDCl_3$

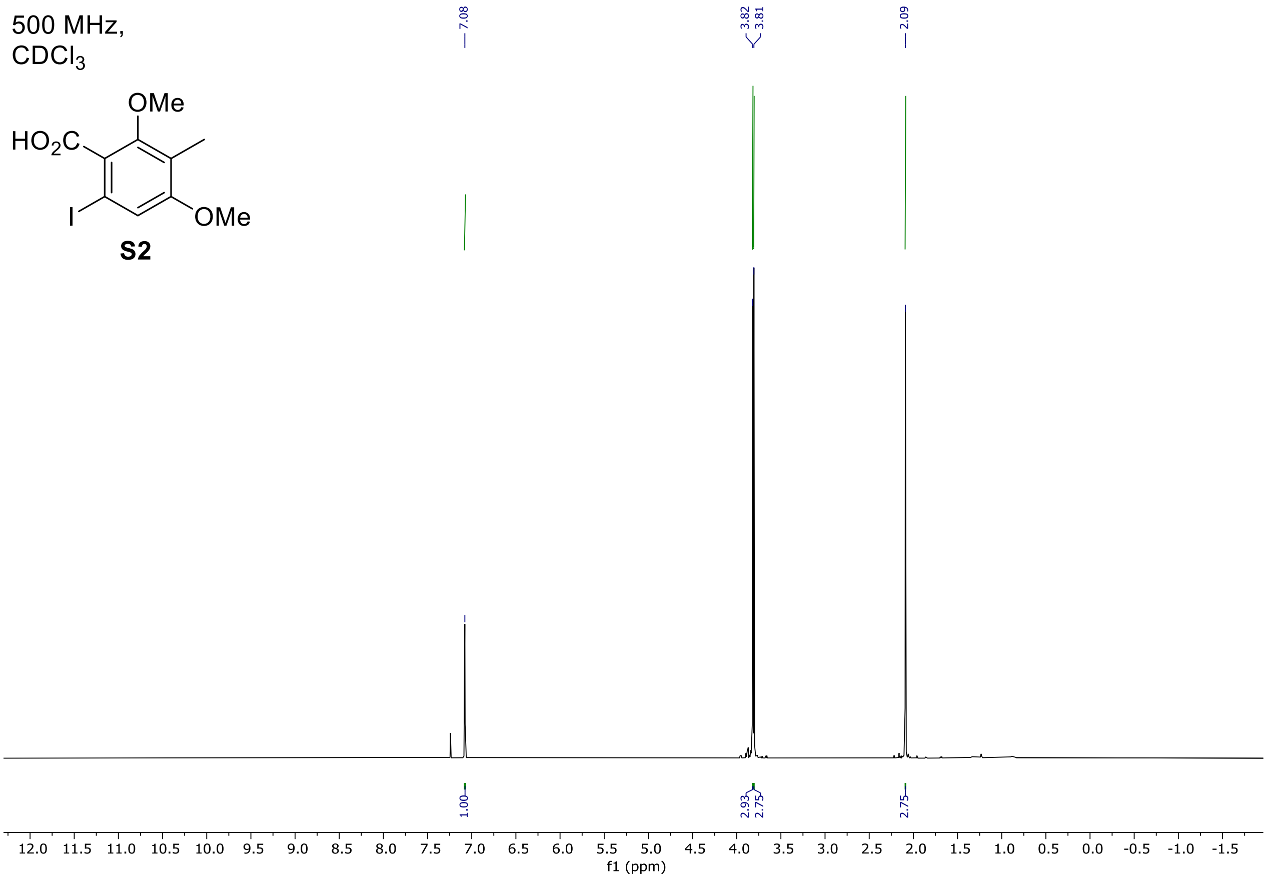

126 MHz,
$CDCl_3$

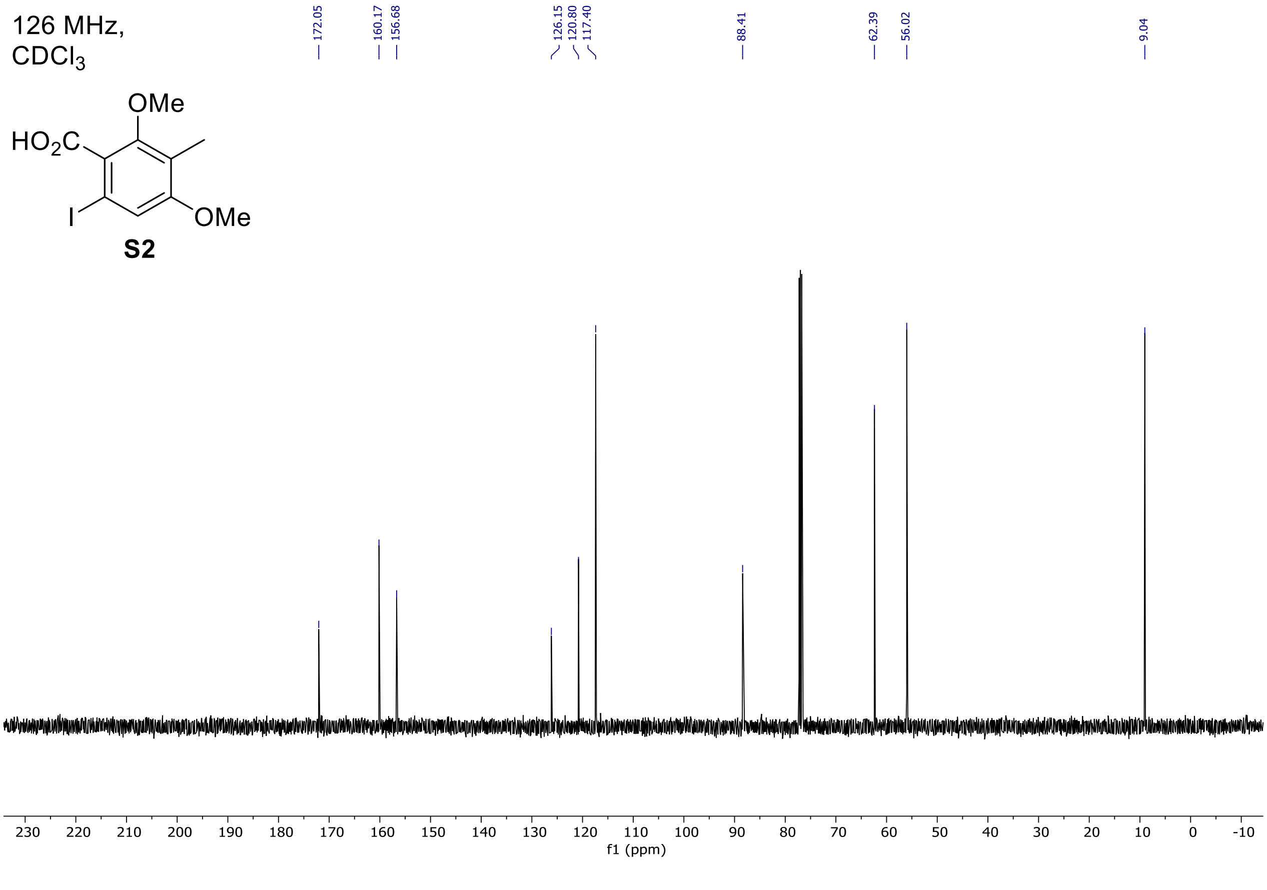

500 MHz,
$CDCl_3$

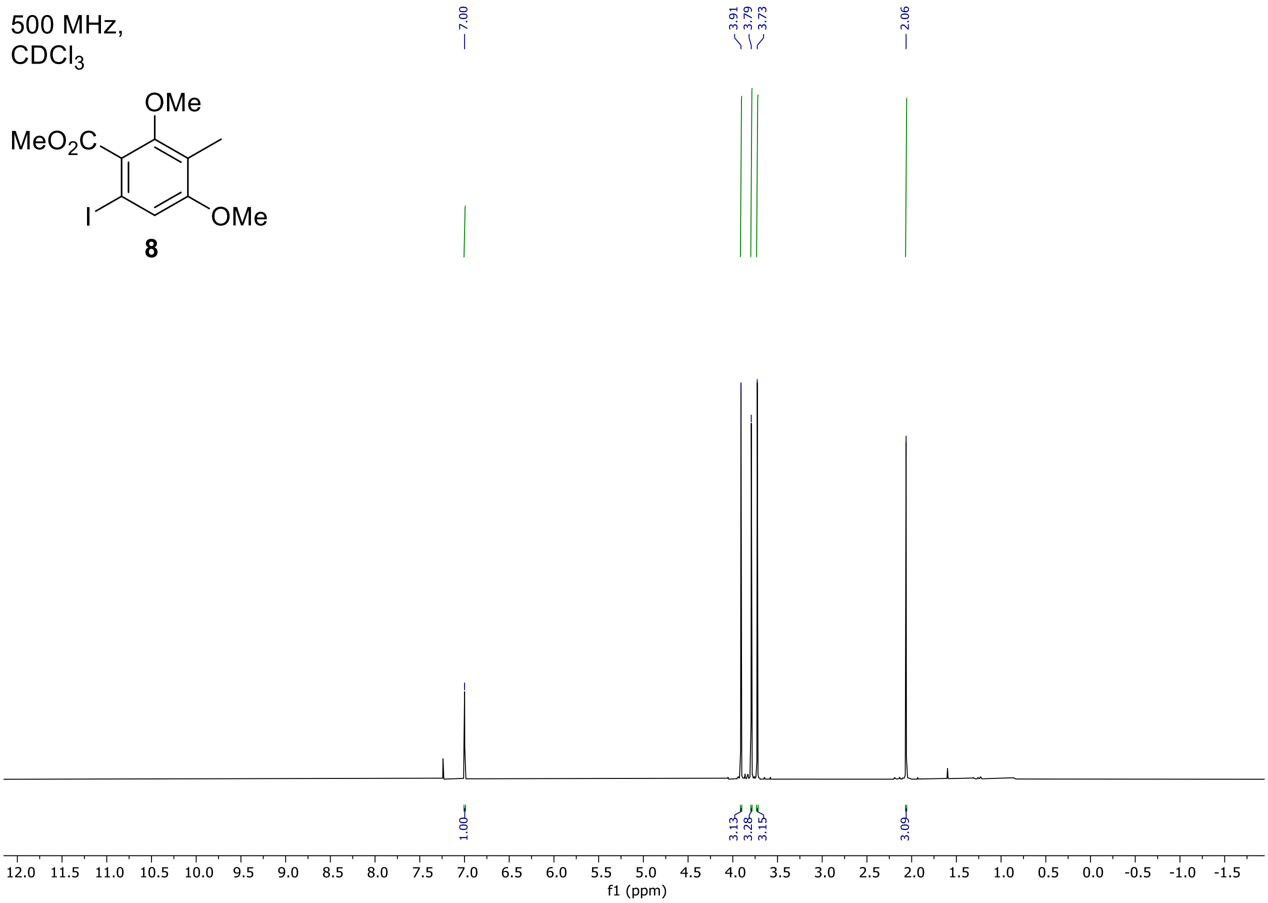

8

126 MHz,
$CDCl_3$

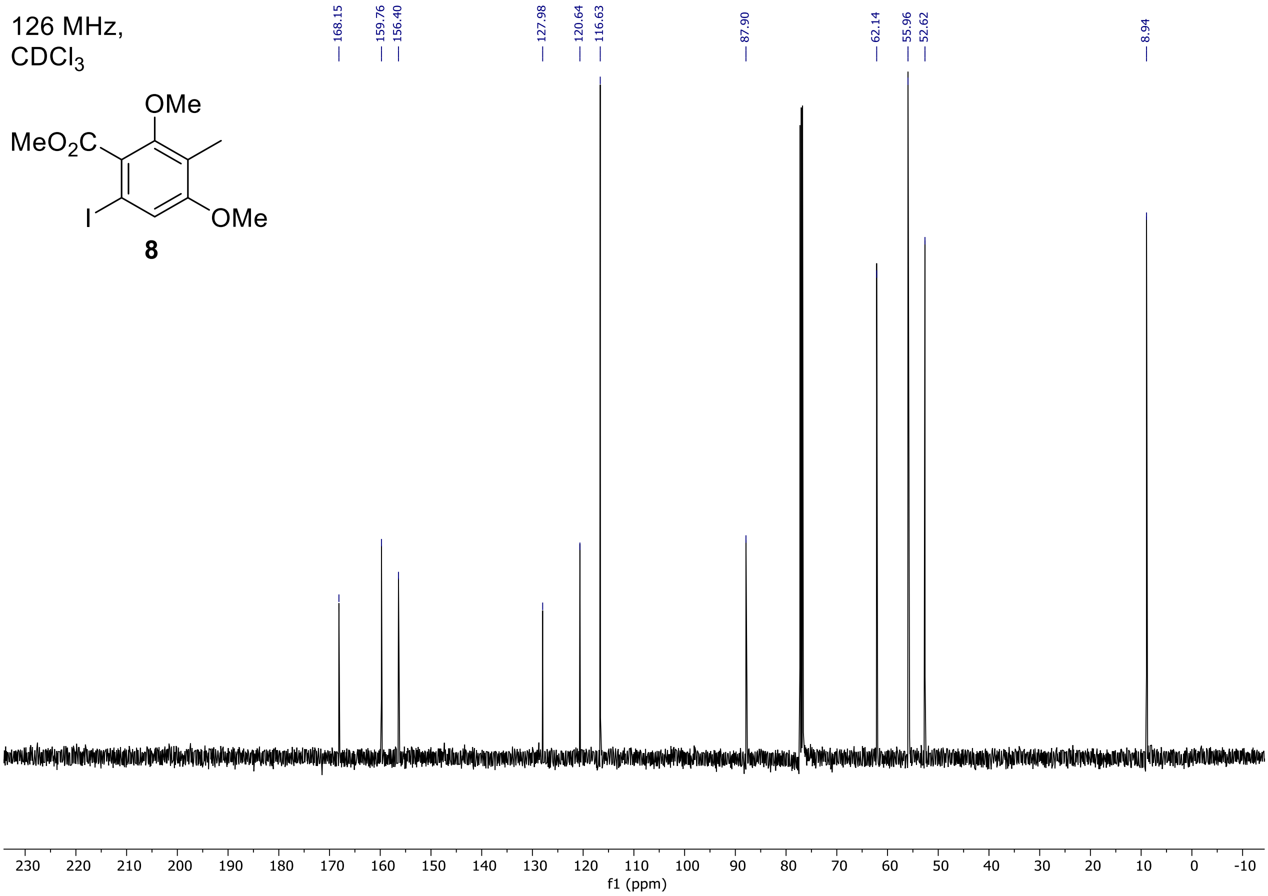

8

600 MHz,
$CDCl_3$

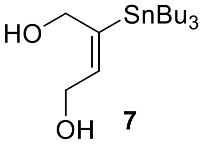

7

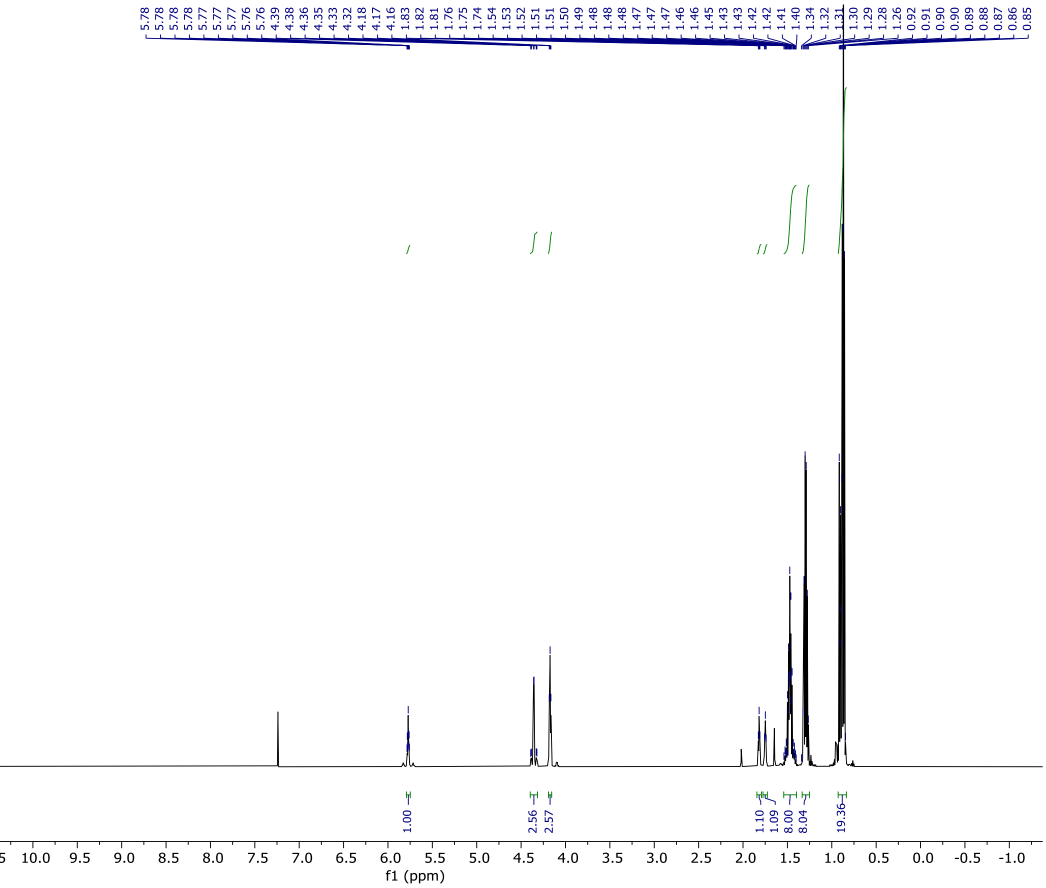

151 MHz,
$CDCl_3$

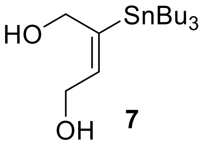

7

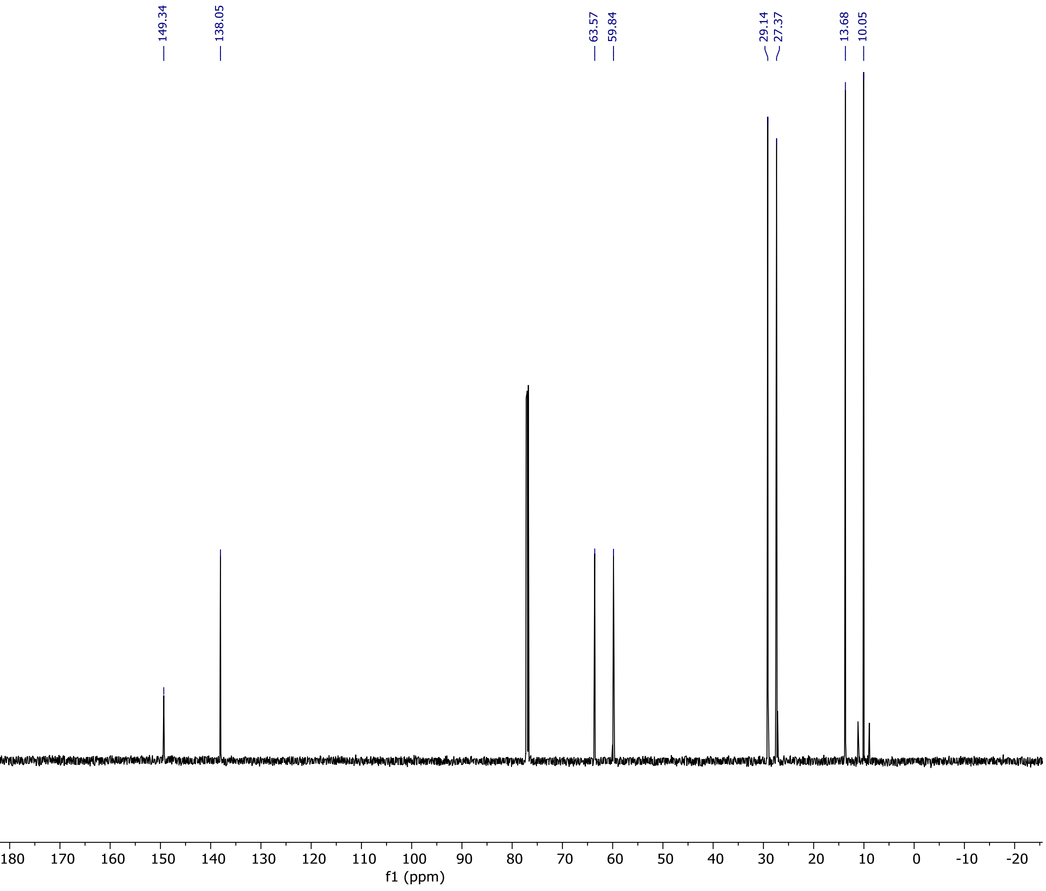

600 MHz,
$CDCl_3$

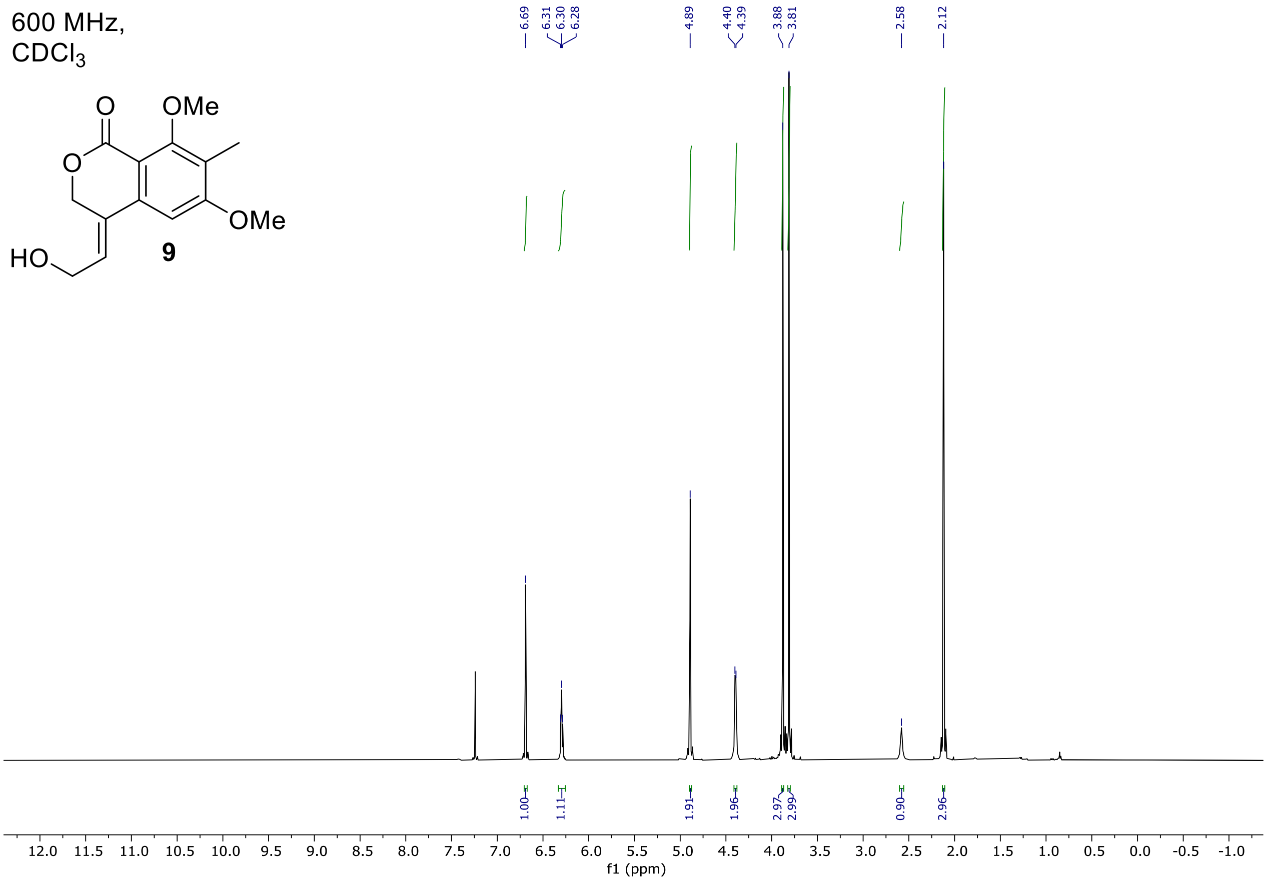

151 MHz,
$CDCl_3$

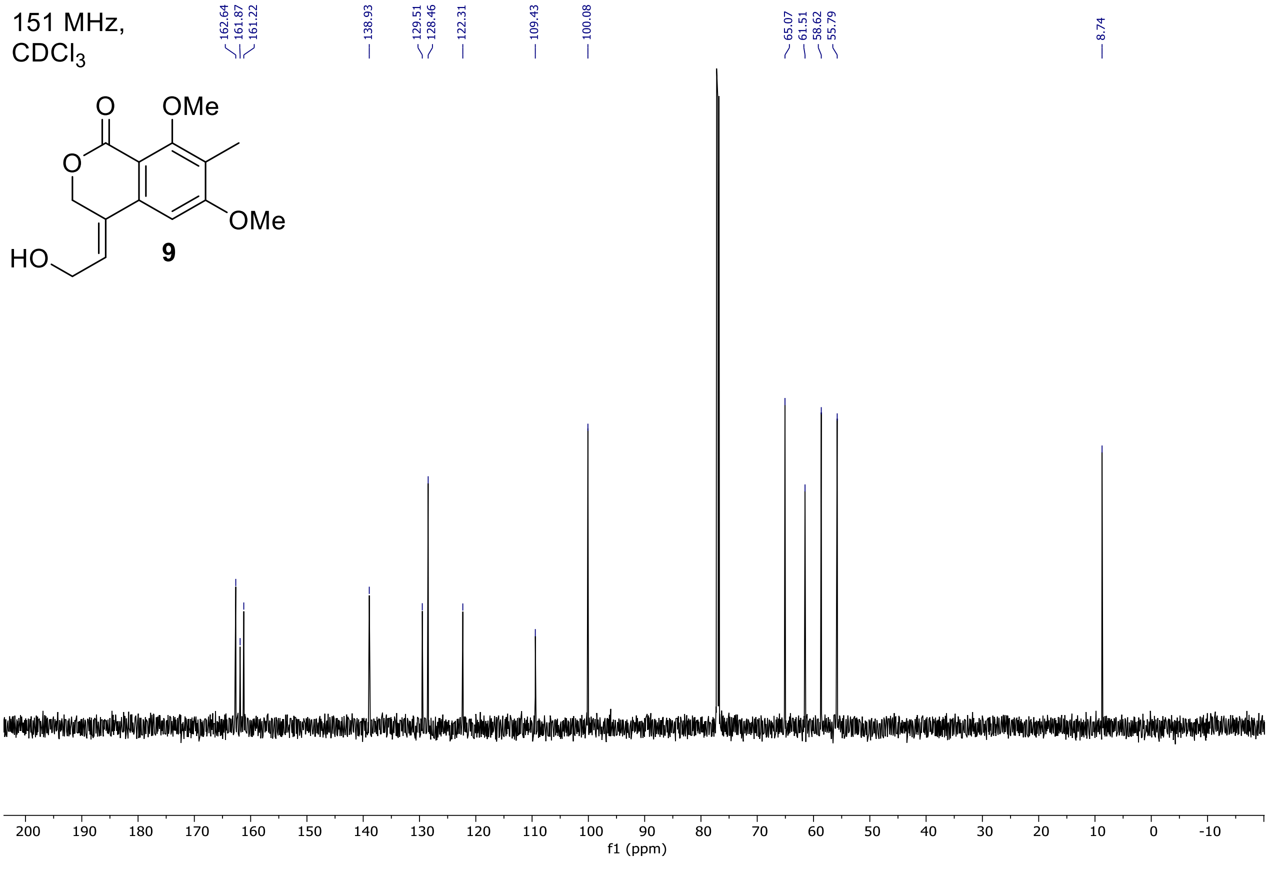

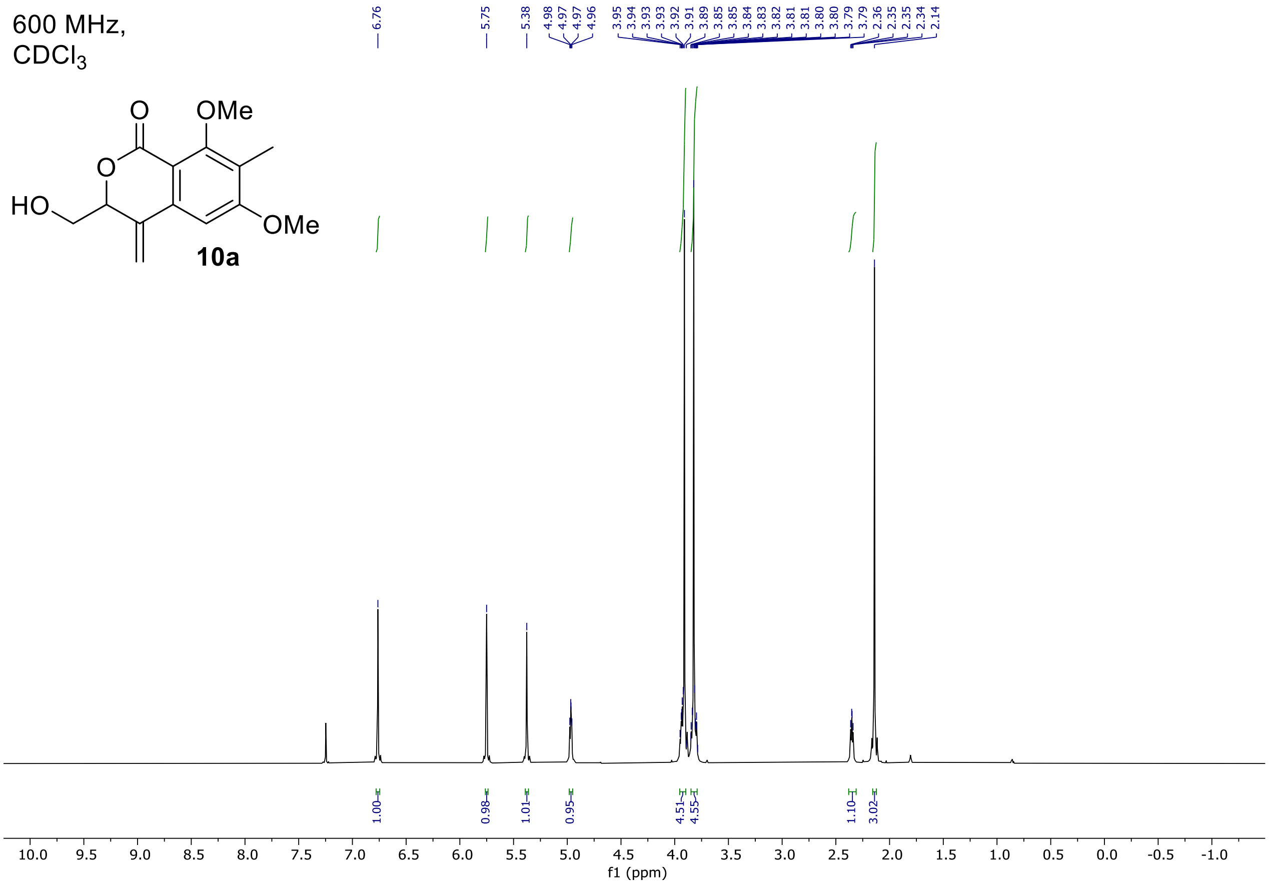
600 MHz,
CDCl3
OMe
OMe
HO
10a
f1 (ppm)

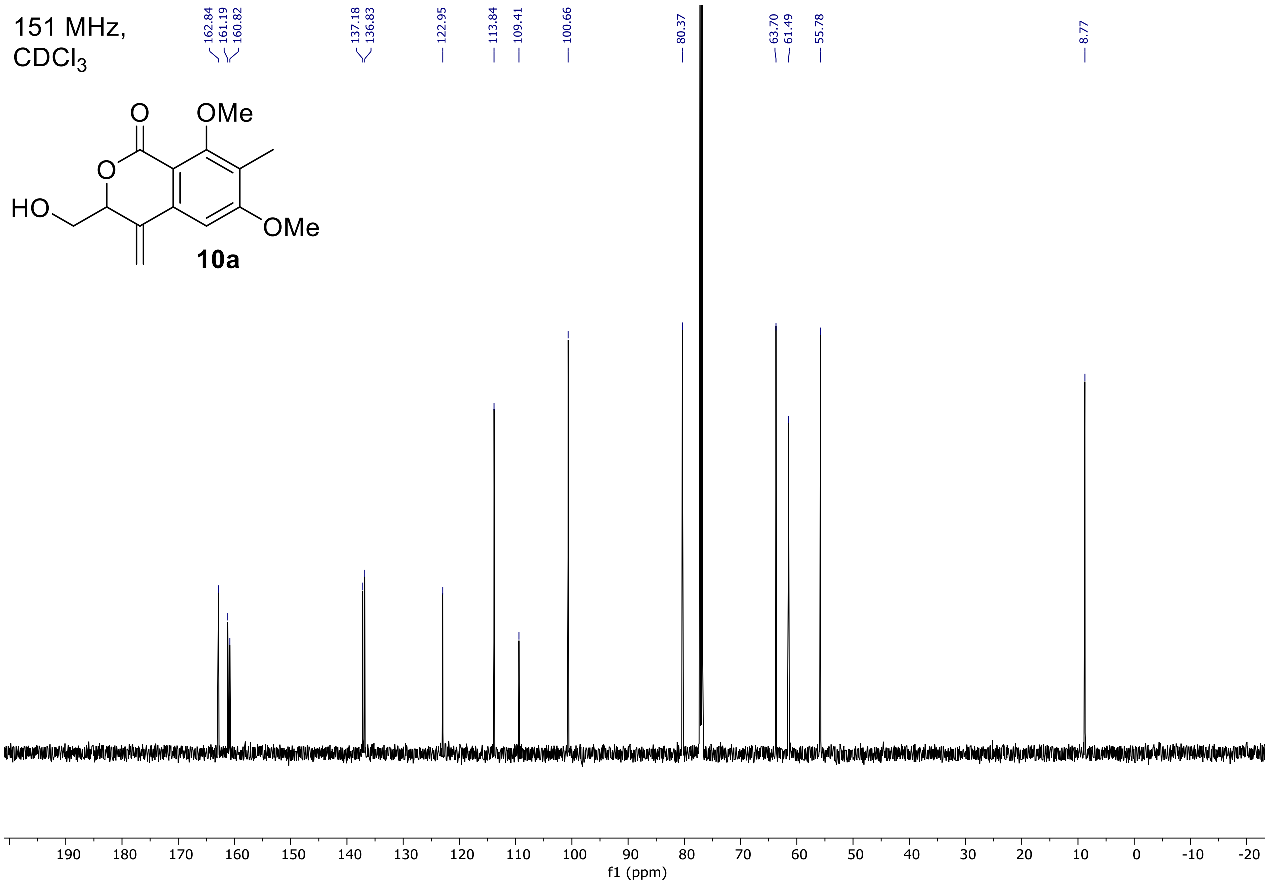
151 MHz,
CDCl3
OMe
OMe
HO
10a
f1 (ppm)

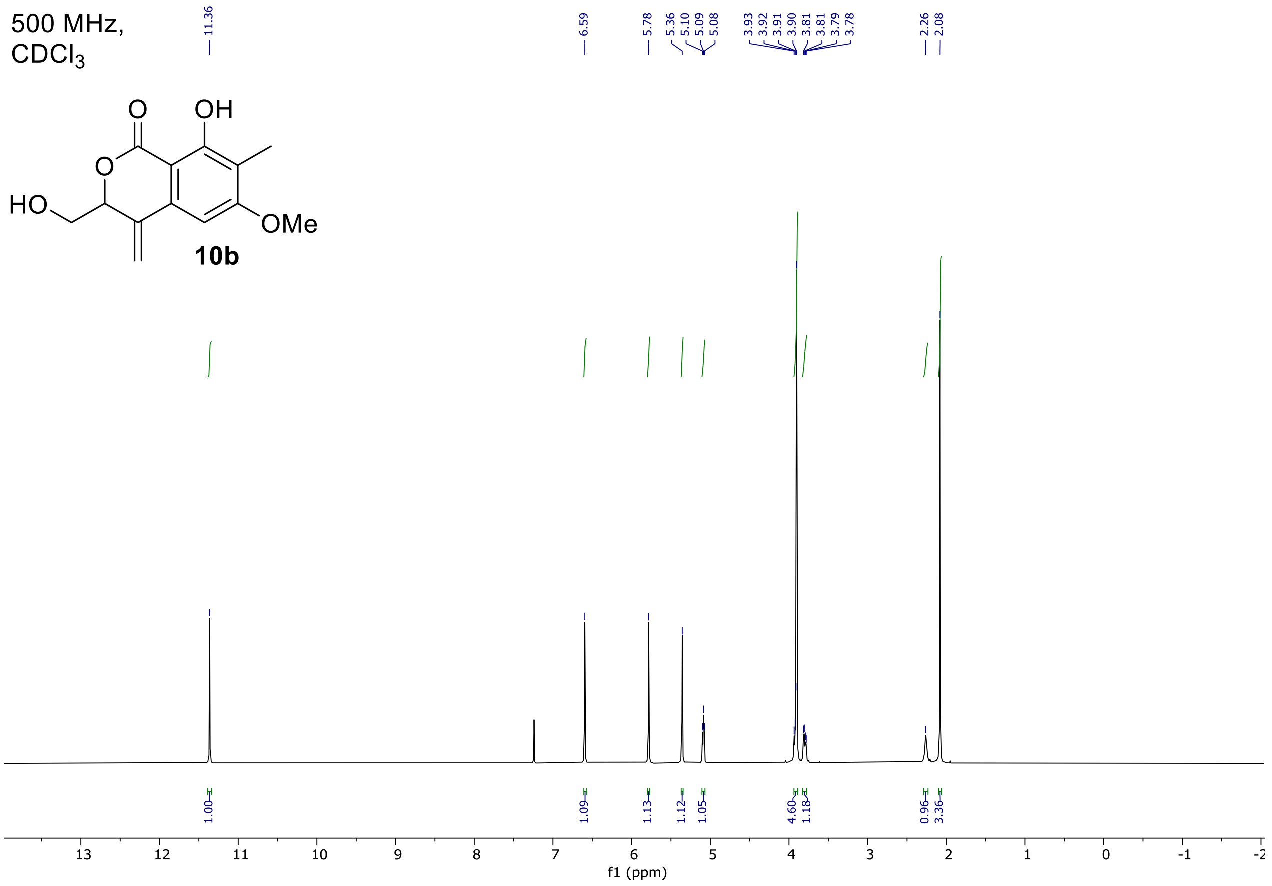
500 MHz,
$CDCl_3$
O
OH
O
HO
OMe
10b
11.36
6.59
5.78
5.36
5.10
5.09
5.08
3.93
3.92
3.91
3.90
3.81
3.81
3.79
3.78
2.26
2.08
1.00
1.09
1.13
1.12
1.05
4.60
1.18
0.96
3.36
13 12 11 10 9 8 7 6 5 4 3 2 1 0 -1 -2
f1 (ppm)

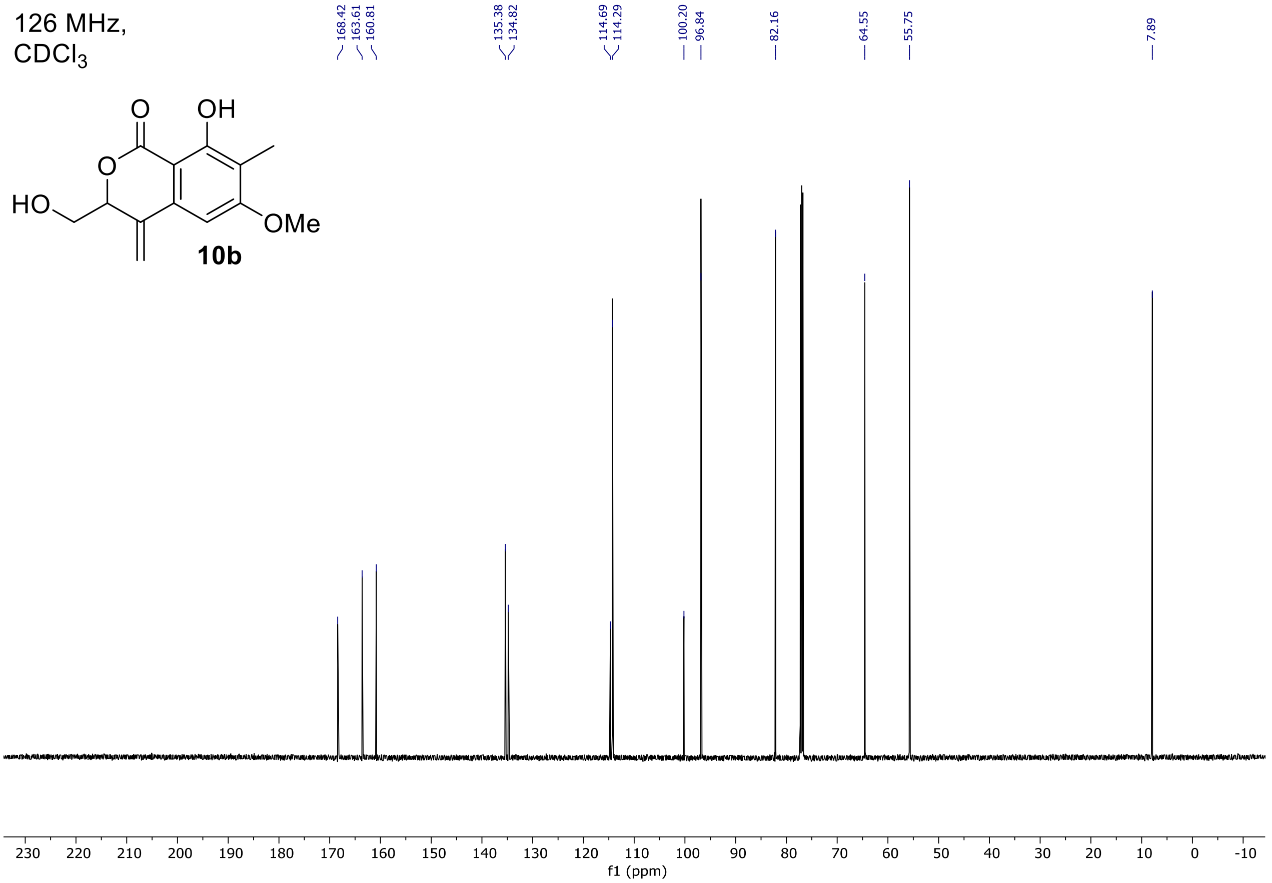
126 MHz,
$CDCl_3$
O
OH
O
HO
OMe
10b
168.42
163.61
160.81
135.38
134.82
114.69
114.29
100.20
96.84
82.16
64.55
55.75
7.89
230 220 210 200 190 180 170 160 150 140 130 120 110 100 90 80 70 60 50 40 30 20 10 0 -10
f1 (ppm)

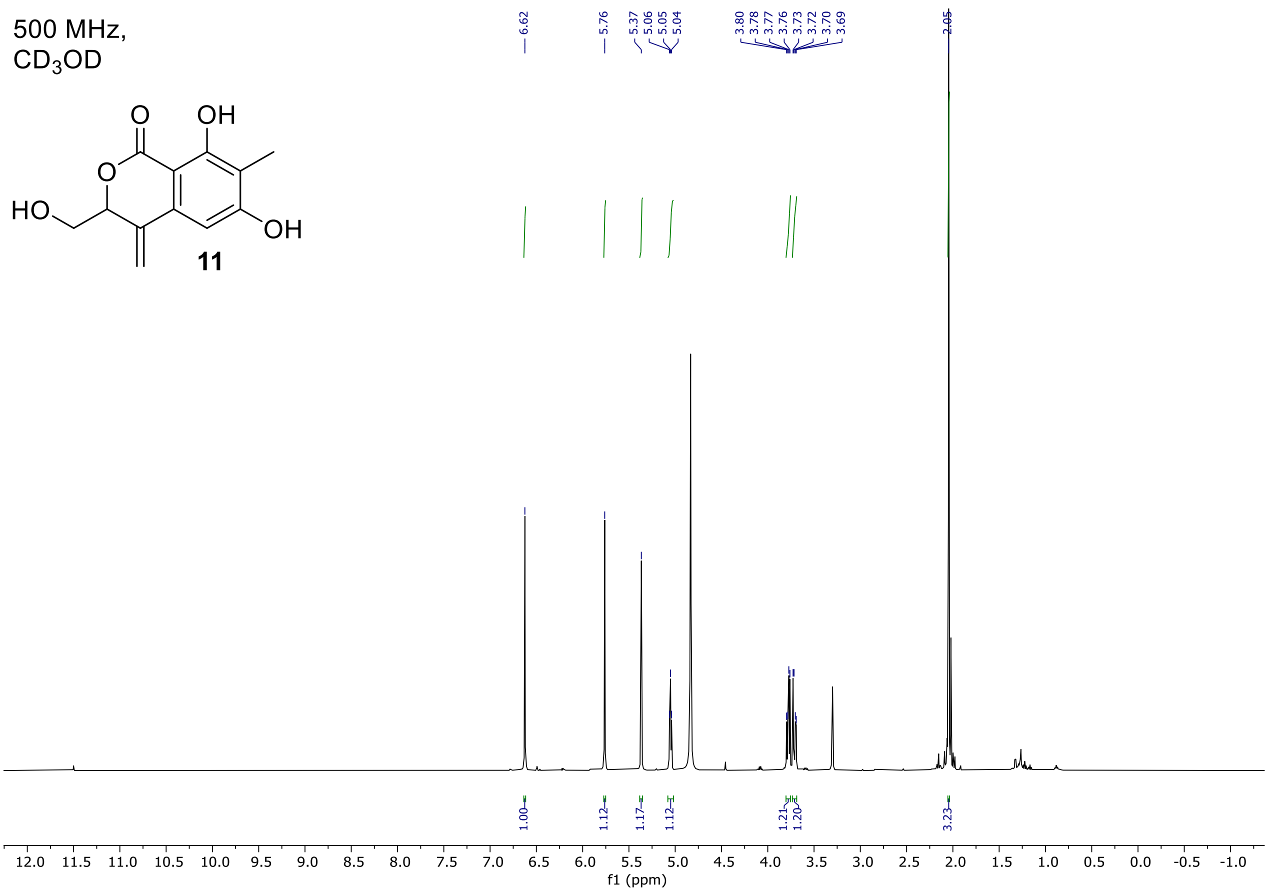
500 MHz,
CD_3OD
O OH
O
HO
OH
11
6.62
5.76
5.37
5.06
5.05
5.04
3.80
3.78
3.77
3.76
3.73
3.72
3.70
3.69
2.05
1.00
1.12
1.17
1.12
1.21
1.20
3.23
12.0 11.5 11.0 10.5 10.0 9.5 9.0 8.5 8.0 7.5 7.0 6.5 6.0 5.5 5.0 4.5 4.0 3.5 3.0 2.5 2.0 1.5 1.0 0.5 0.0 -0.5 -1.0
f1 (ppm)

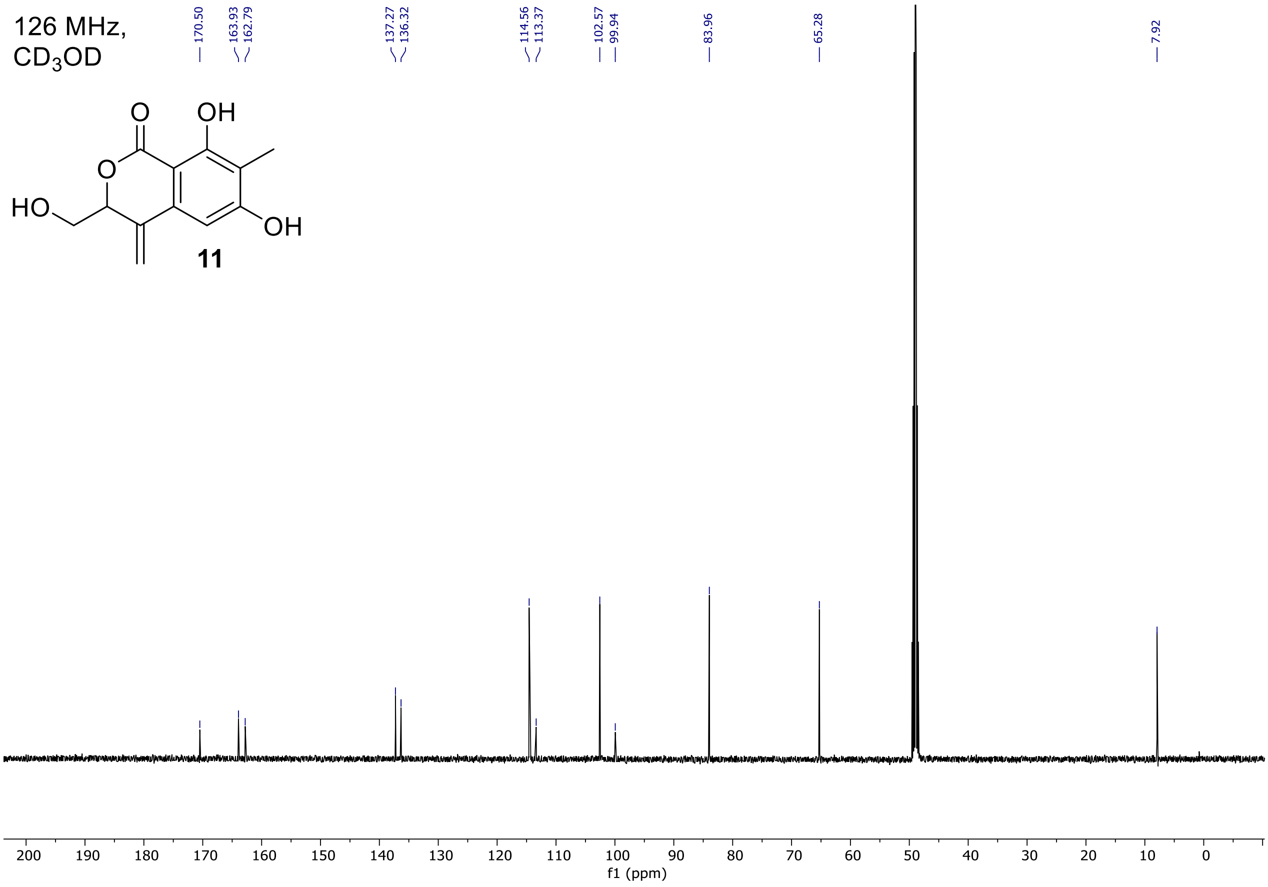
126 MHz,
CD_3OD
O OH
O
HO
OH
11
170.50
163.93
162.79
137.27
136.32
114.56
113.37
102.57
99.94
83.96
65.28
7.92
200 190 180 170 160 150 140 130 120 110 100 90 80 70 60 50 40 30 20 10 0
f1 (ppm)

600 MHz,
acetonitrile-d_6

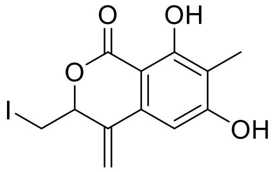

12

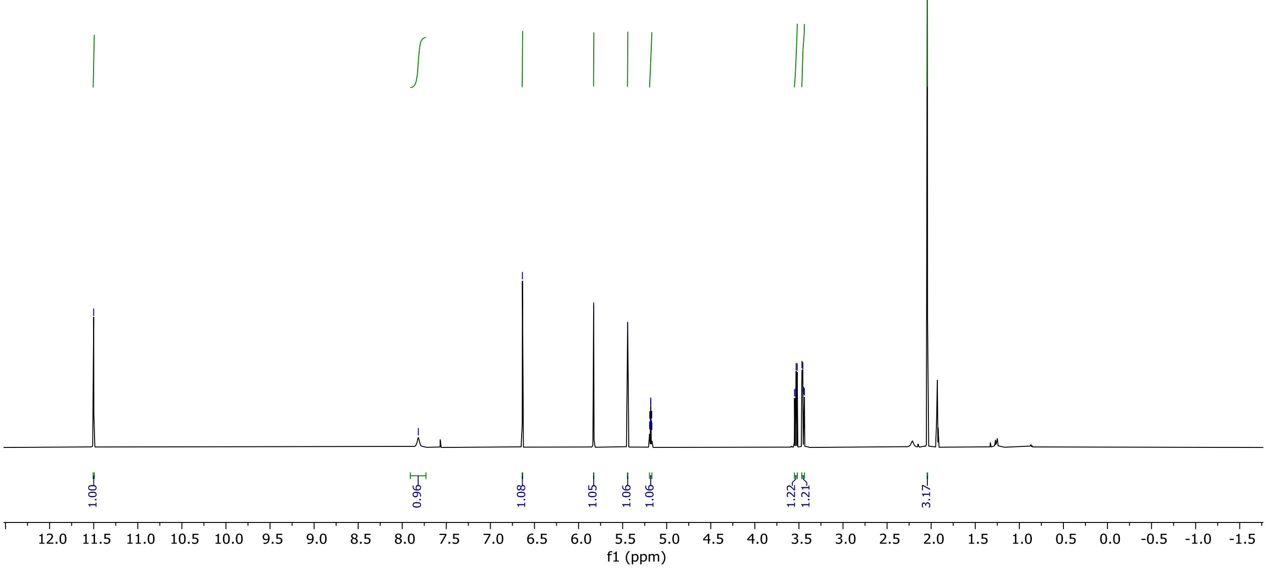

151 MHz,
acetonitrile-d_6

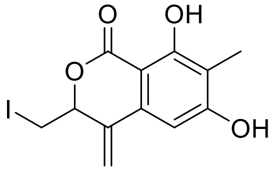

12

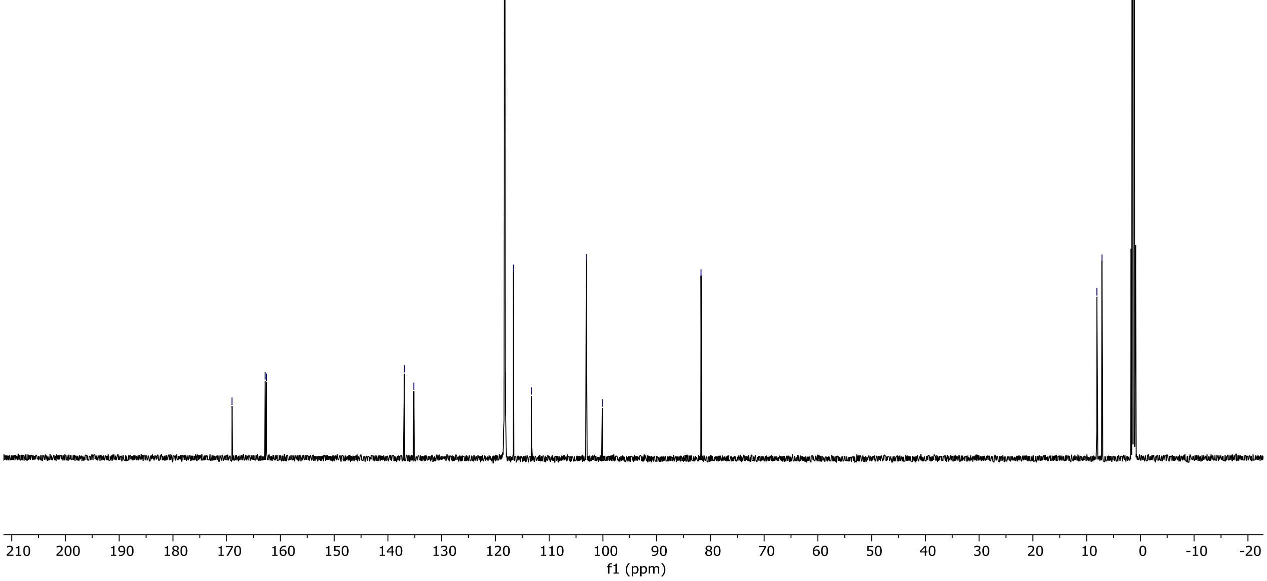

600 MHz,
DMSO-d_6

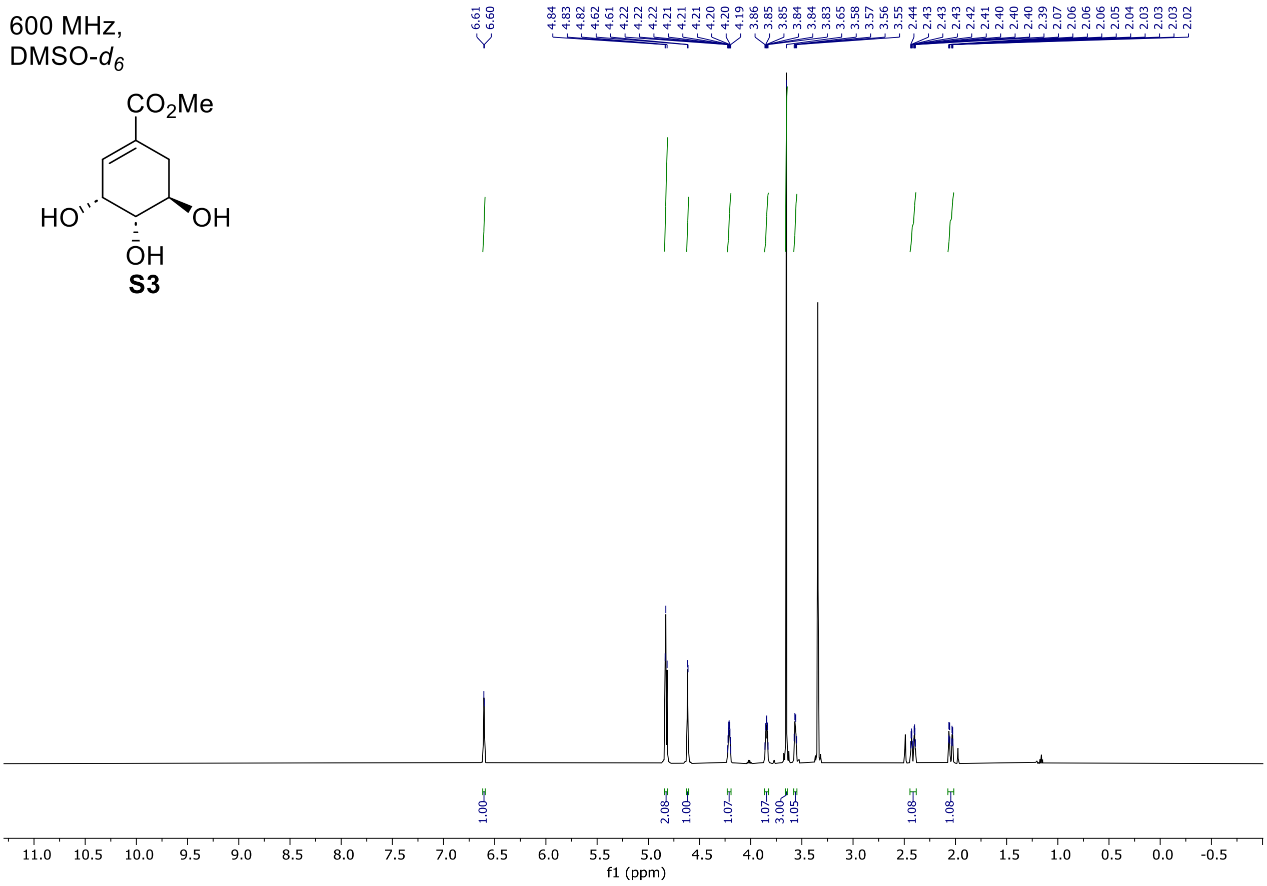

151 MHz,
DMSO-d_6

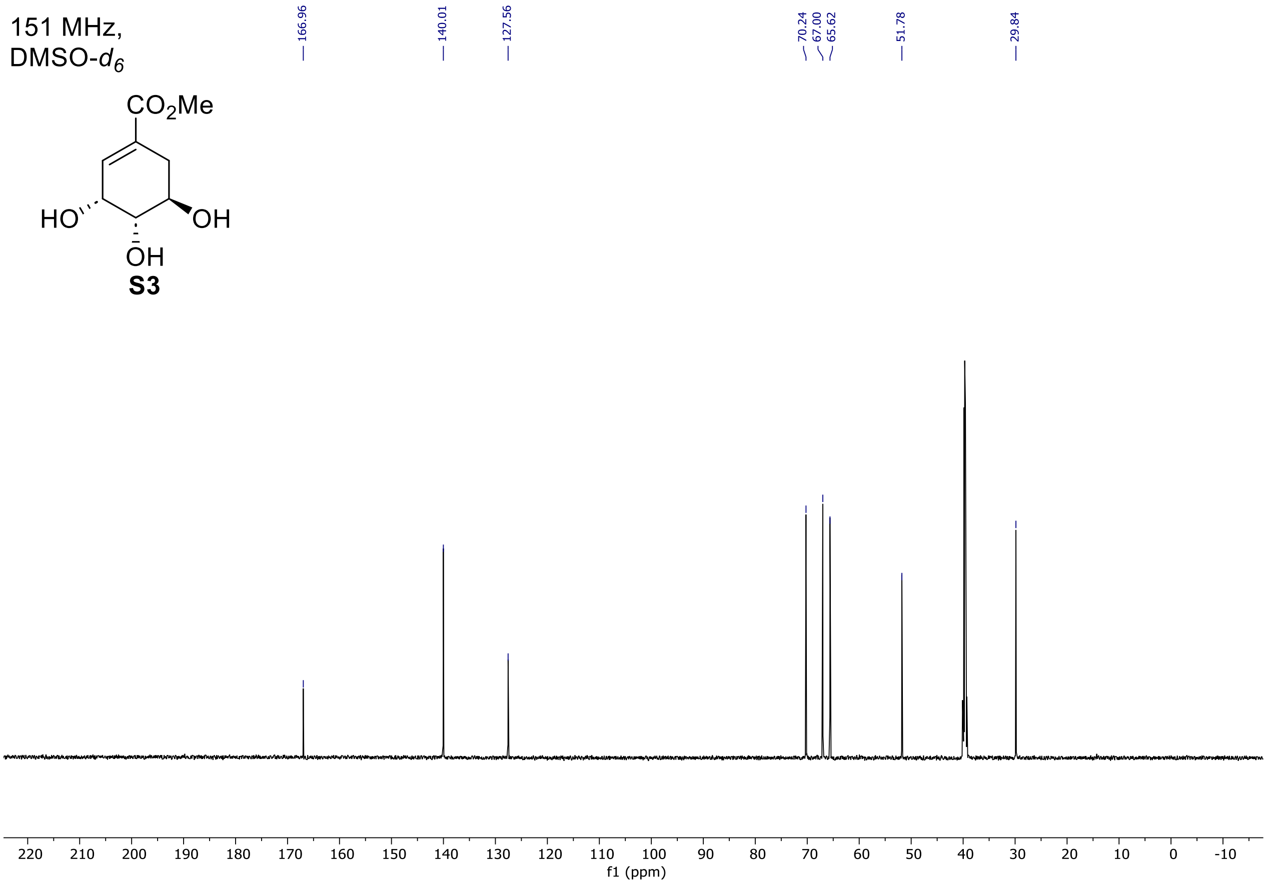

600 MHz, $CDCl_3$

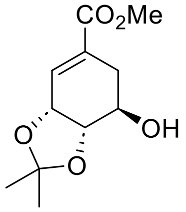

S4

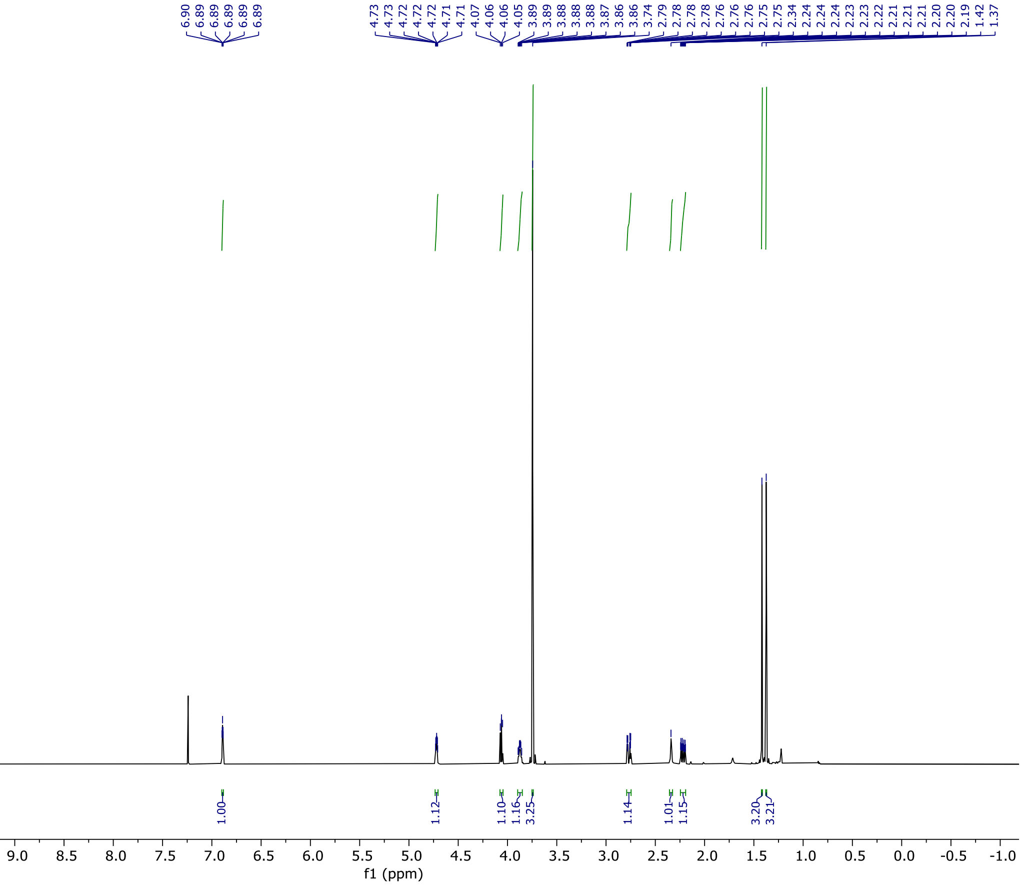

151 MHz, $CDCl_3$

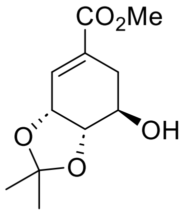

S4

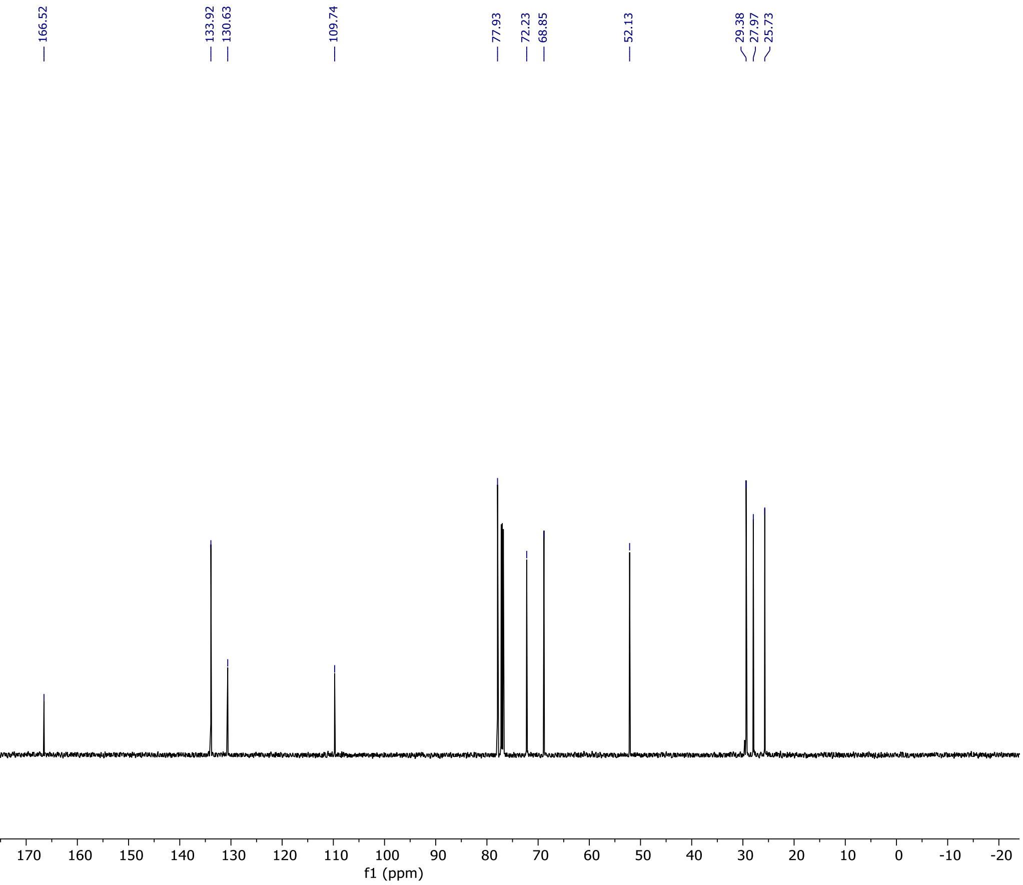

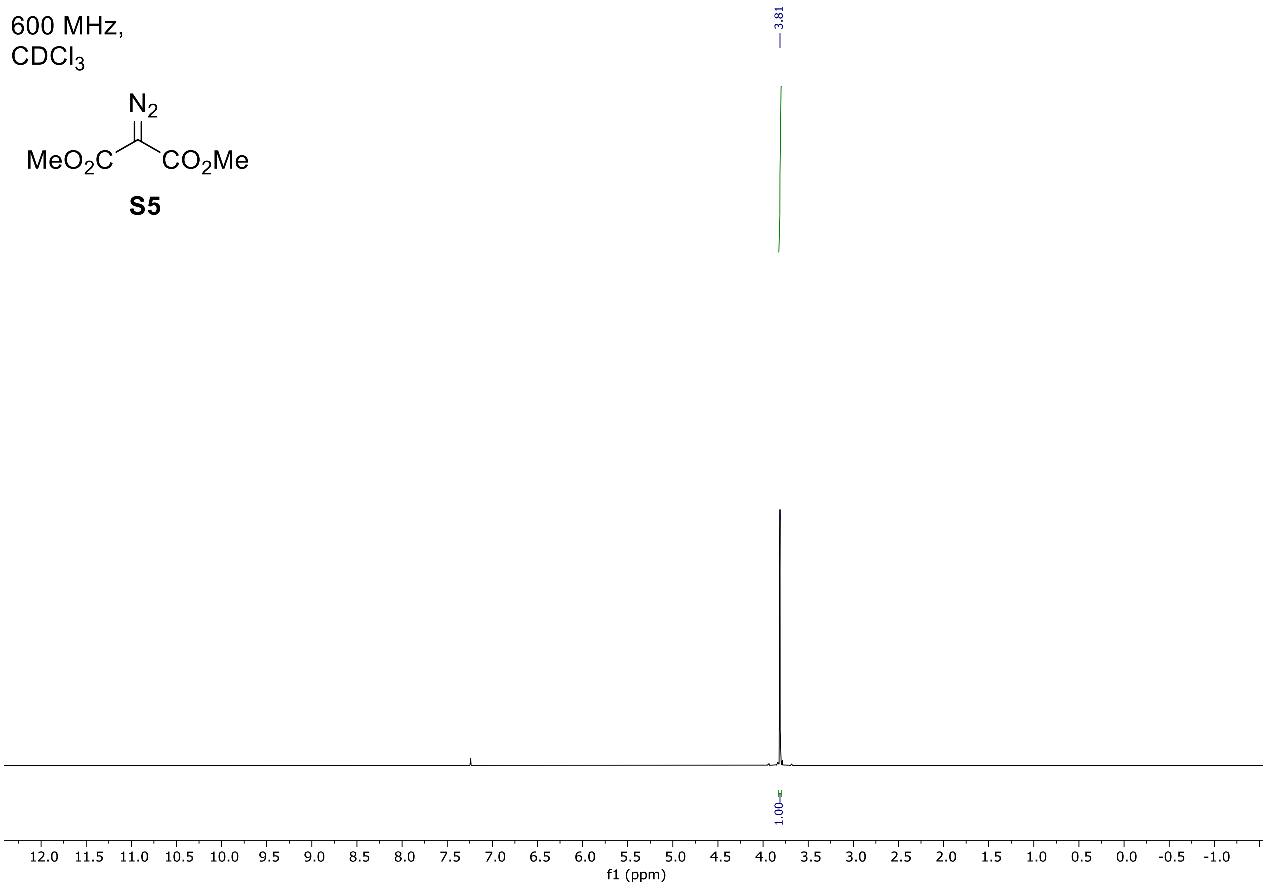
600 MHz,
$CDCl_3$
N_2
MeO_2C CO_2Me
S5
3.81
1.00
12.0 11.5 11.0 10.5 10.0 9.5 9.0 8.5 8.0 7.5 7.0 6.5 6.0 5.5 5.0 4.5 4.0 3.5 3.0 2.5 2.0 1.5 1.0 0.5 0.0 -0.5 -1.0
f1 (ppm)

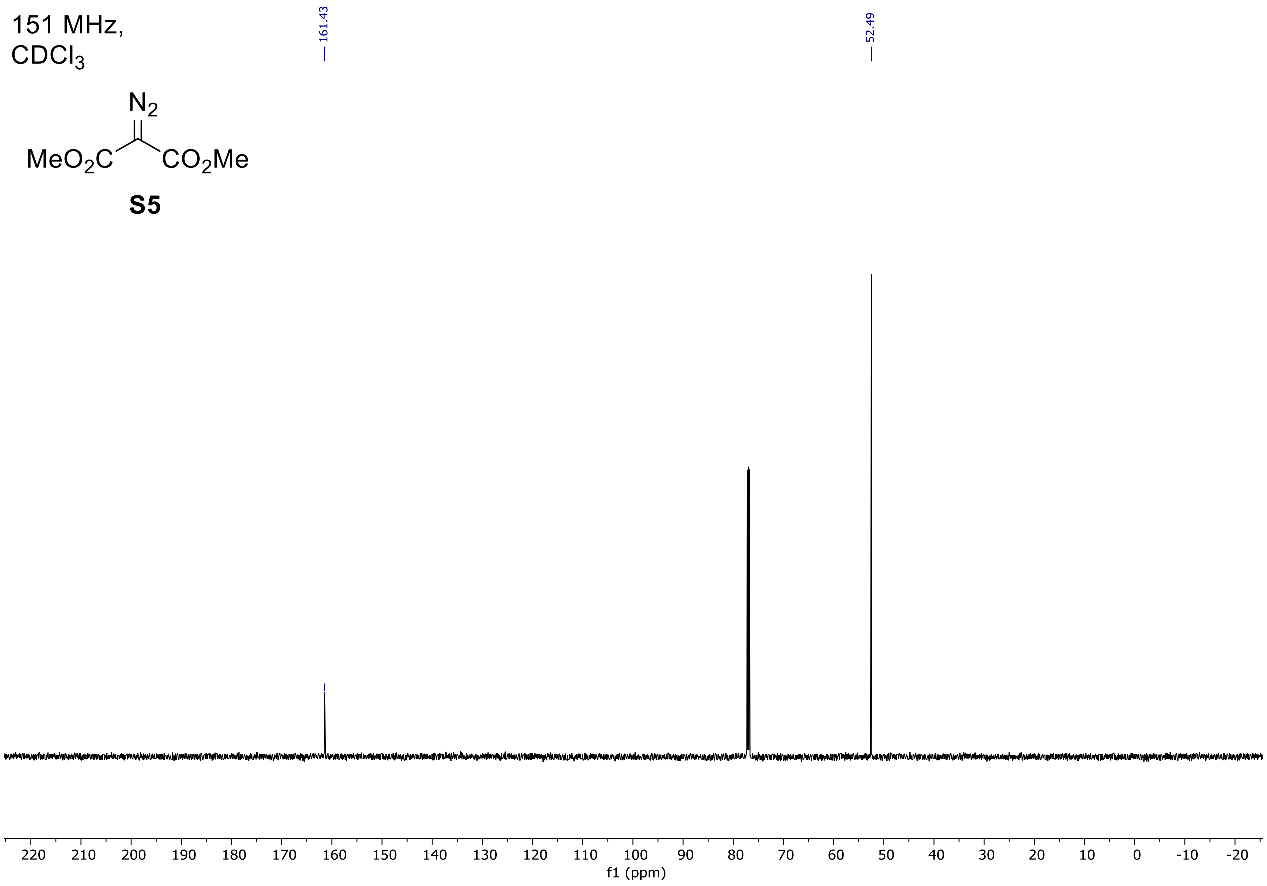
151 MHz,
$CDCl_3$
N_2
MeO_2C CO_2Me
S5
161.43
52.49
220 210 200 190 180 170 160 150 140 130 120 110 100 90 80 70 60 50 40 30 20 10 0 -10 -20
f1 (ppm)

500 MHz,
$CDCl_3$

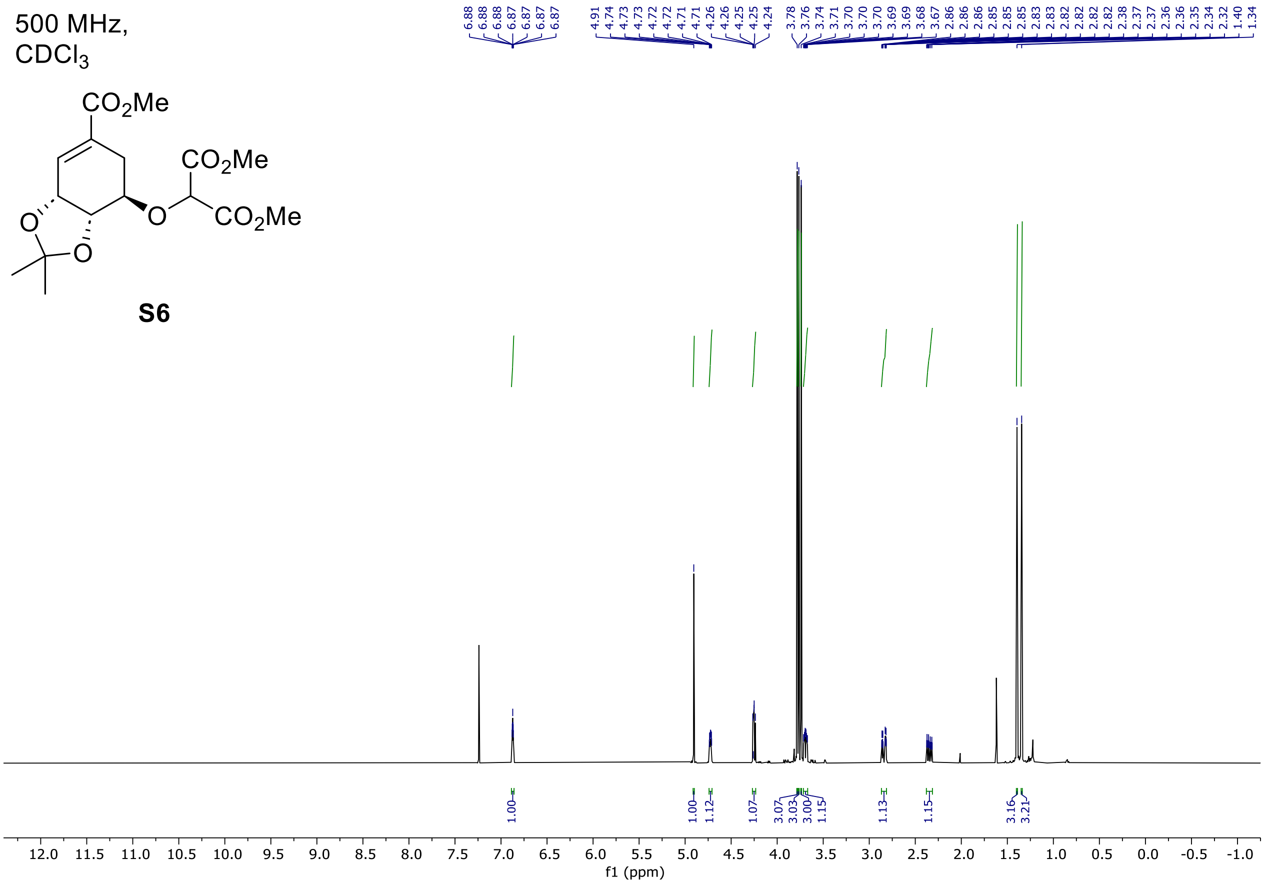

126 MHz,
$CDCl_3$

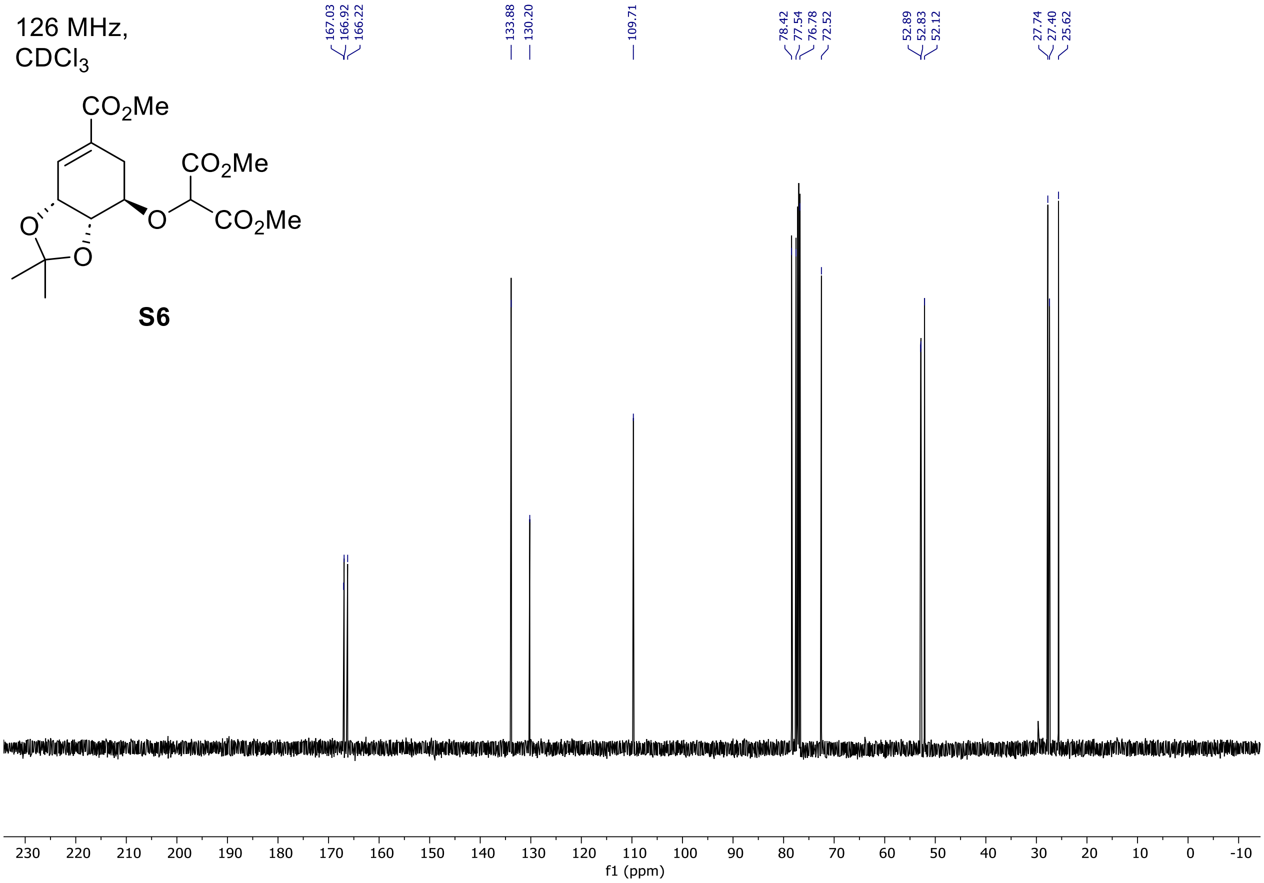

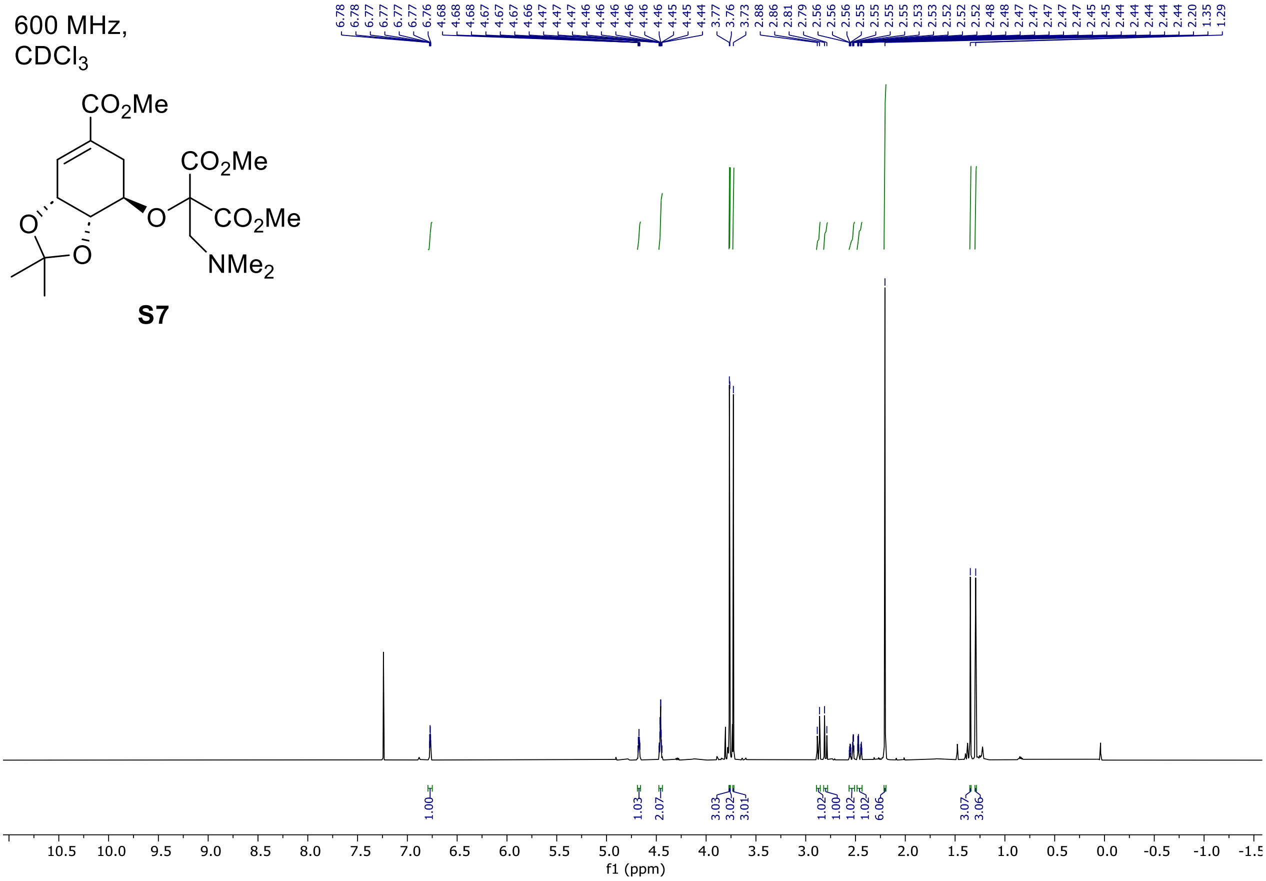
600 MHz,
$CDCl_3$
CO_2Me
CO_2Me
CO_2Me
NMe_2
S7
f1 (ppm)

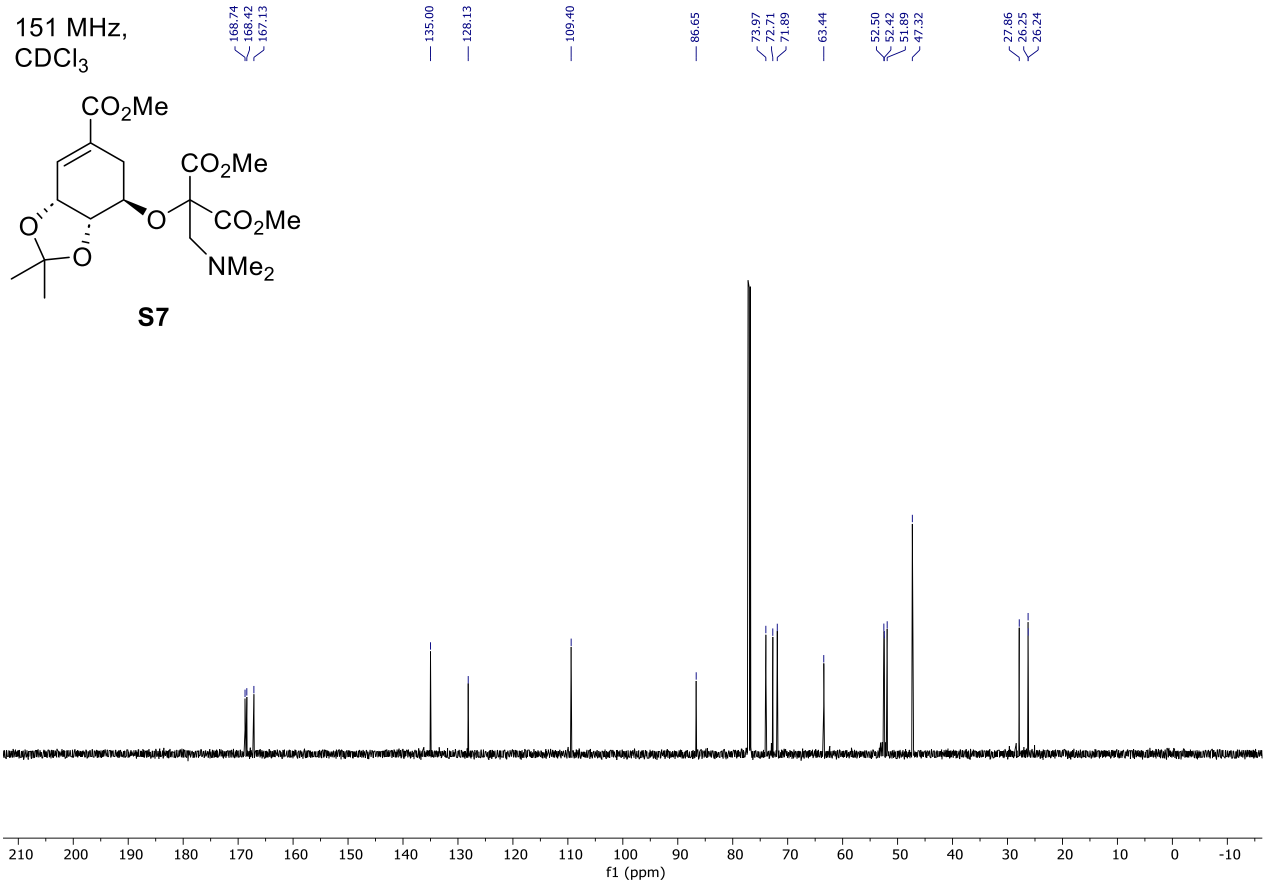
151 MHz,
$CDCl_3$
168.74
168.42
167.13
135.00
128.13
109.40
86.65
73.97
72.71
71.89
63.44
52.50
52.42
51.89
47.32
27.86
26.25
26.24
CO_2Me
CO_2Me
CO_2Me
NMe_2
S7
f1 (ppm)

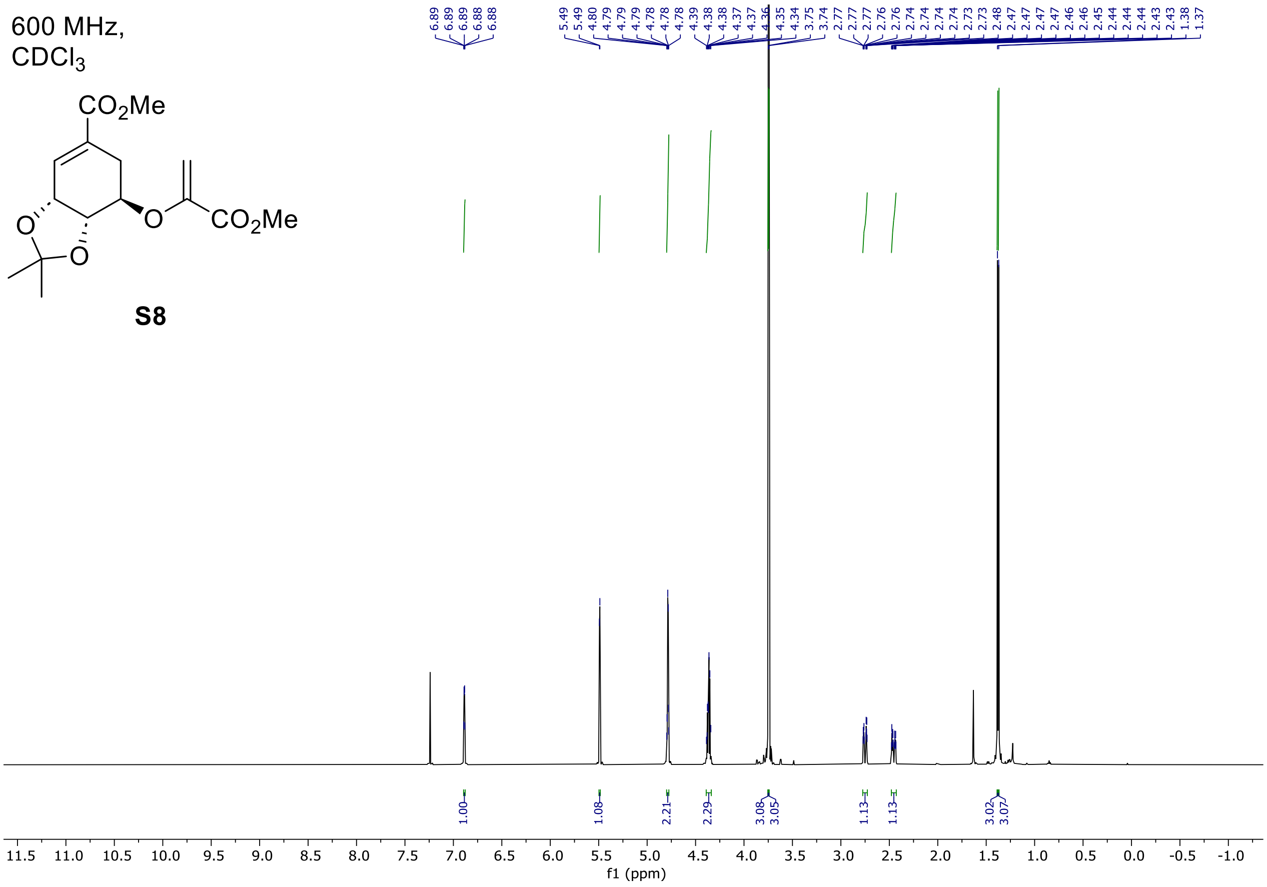
600 MHz,
CDCl3
CO2Me
O
O
O
CO2Me
S8
6.89 6.89 6.89 6.88 6.88
5.49 5.49 4.80 4.79 4.79 4.79 4.78 4.78 4.78 4.39 4.38 4.38 4.37 4.37 4.36 4.35 4.34 3.75 3.74 2.77 2.77 2.77 2.76 2.76 2.74 2.74 2.74 2.74 2.73 2.73 2.48 2.47 2.47 2.47 2.47 2.46 2.46 2.45 2.44 2.44 2.44 2.43 2.43 1.38 1.37
1.00 1.08 2.21 2.29 3.08 3.05 1.13 1.13 3.02 3.07
11.5 11.0 10.5 10.0 9.5 9.0 8.5 8.0 7.5 7.0 6.5 6.0 5.5 5.0 4.5 4.0 3.5 3.0 2.5 2.0 1.5 1.0 0.5 0.0 -0.5 -1.0
f1 (ppm)

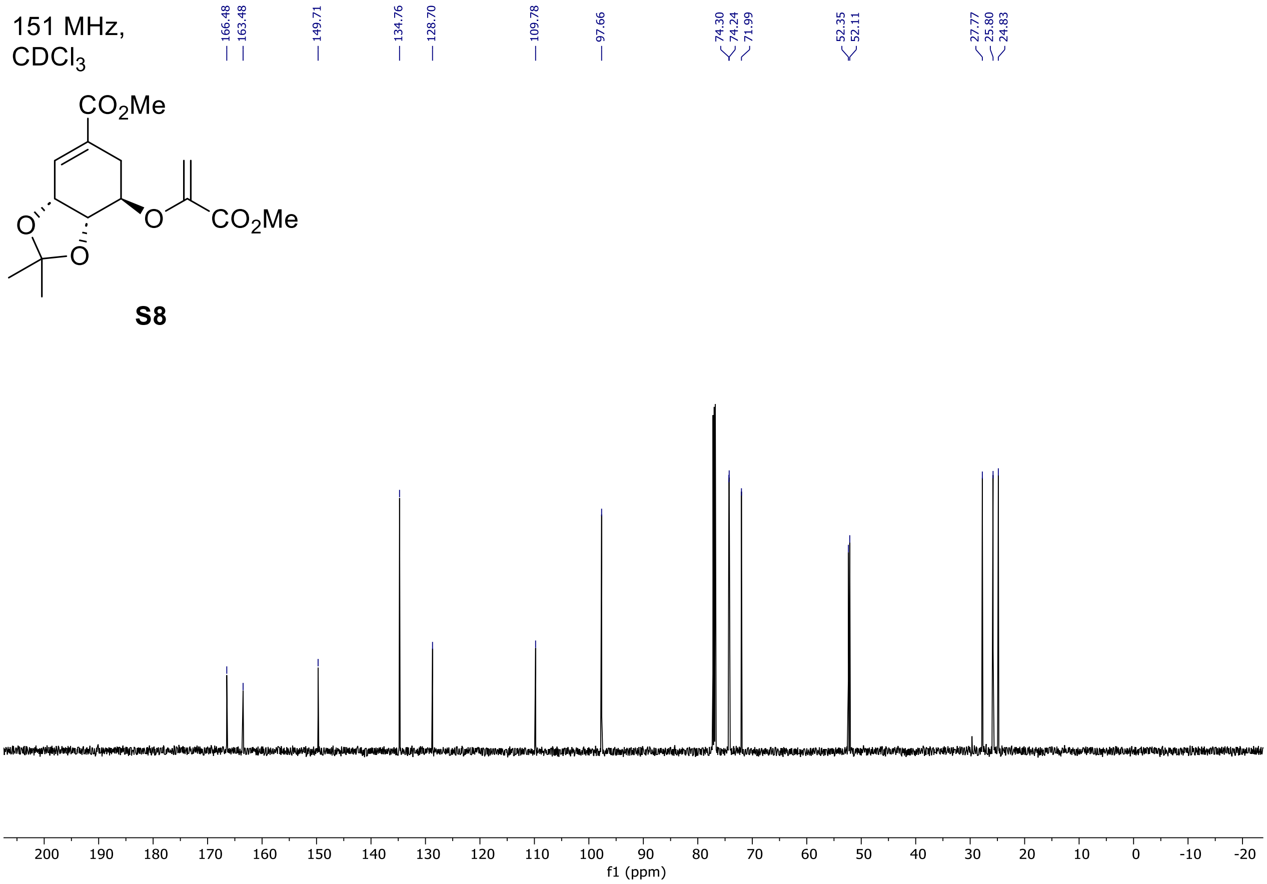
151 MHz,
CDCl3
CO2Me
O
O
O
CO2Me
S8
166.48 163.48 149.71 134.76 128.70 109.78 97.66 74.30 74.24 71.99 52.35 52.11 27.77 25.80 24.83
200 190 180 170 160 150 140 130 120 110 100 90 80 70 60 50 40 30 20 10 0 -10 -20
f1 (ppm)

600 MHz,
$CDCl_3$

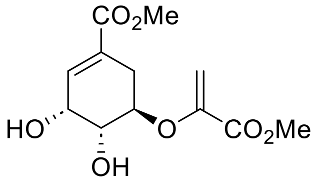

S9

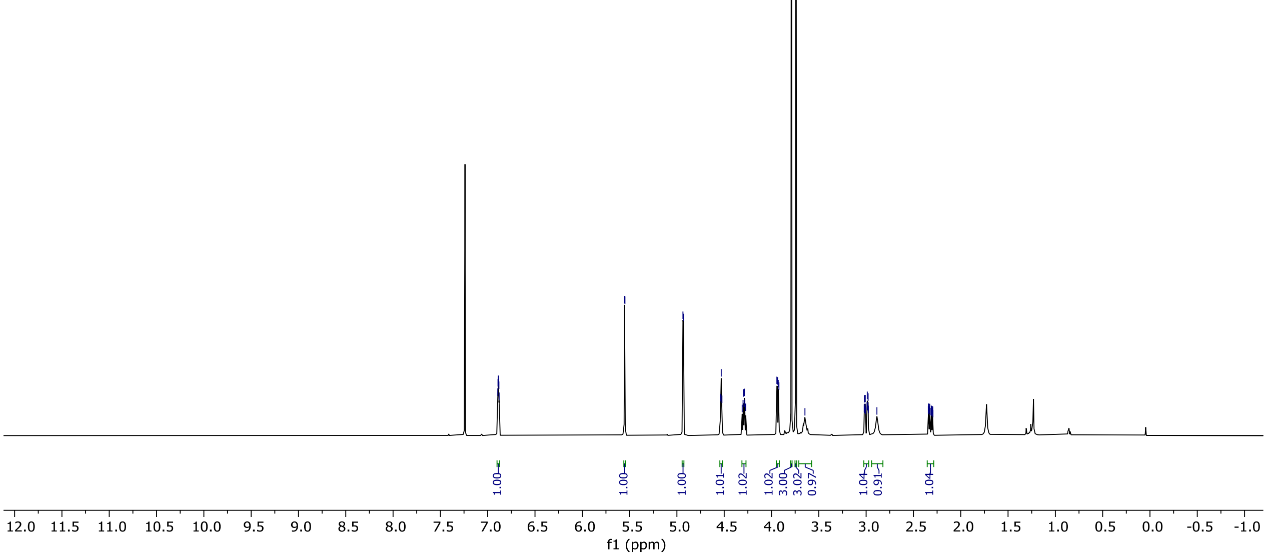

151 MHz,
$CDCl_3$

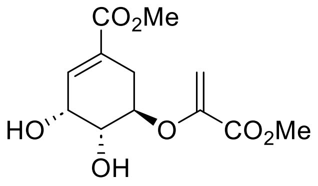

S9

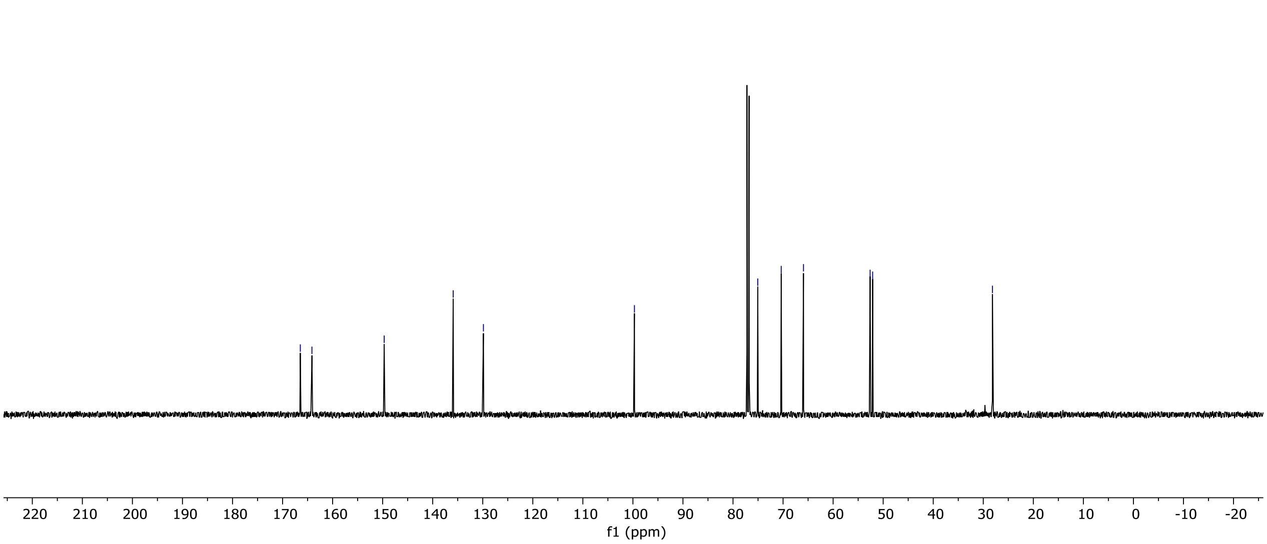

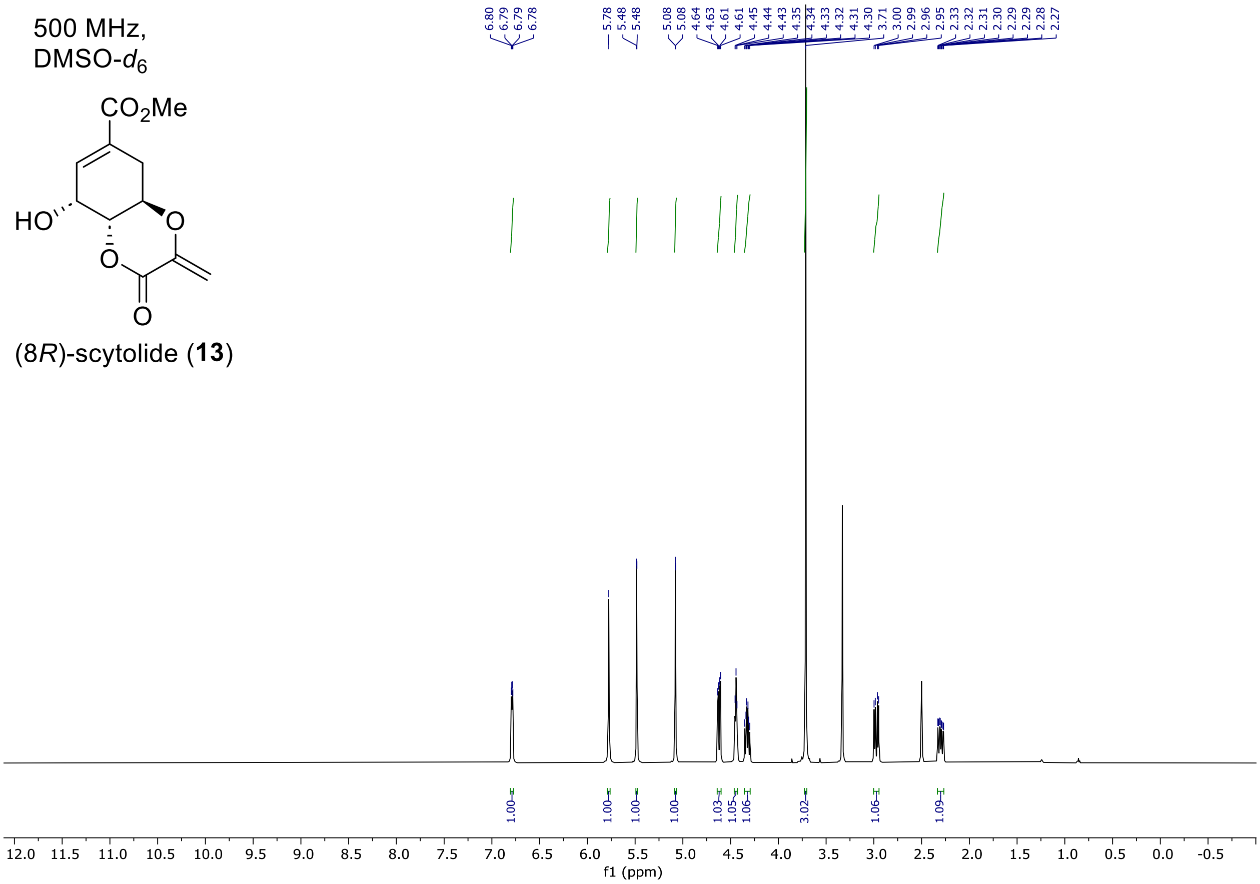
500 MHz,
DMSO-d6
CO2Me
HO
O
O
O
(8R)-scytolide (13)
6.80 6.79 6.79 6.78 5.78 5.48 5.48 5.08 5.08 4.64 4.63 4.61 4.61 4.45 4.44 4.43 4.35 4.34 4.33 4.32 4.31 4.30 3.71 3.00 2.99 2.96 2.95 2.33 2.32 2.31 2.30 2.29 2.29 2.28 2.27
1.00 1.00 1.00 1.00 1.03 1.05 1.06 3.02 1.06 1.09
f1 (ppm)

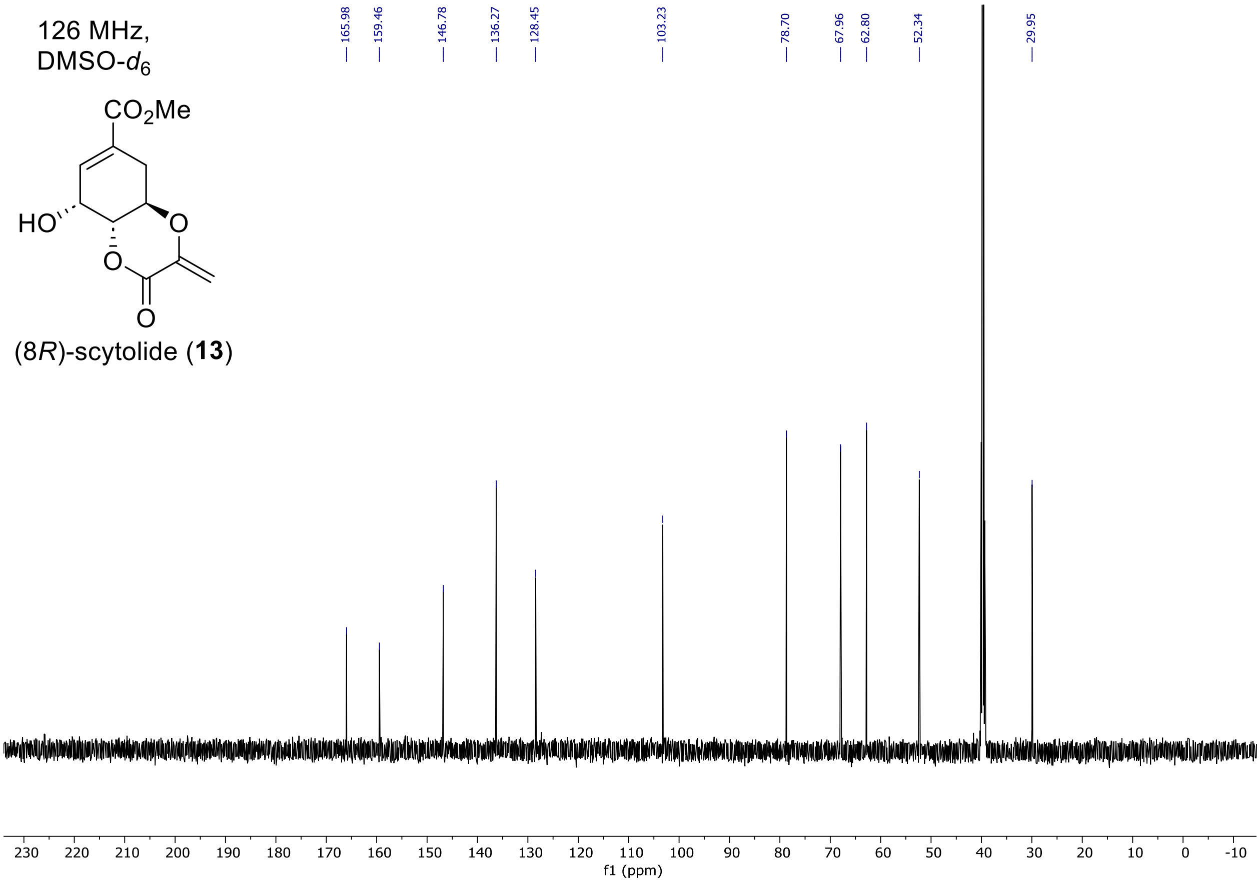
126 MHz,
DMSO-d6
CO2Me
HO
O
O
O
(8R)-scytolide (13)
165.98 159.46 146.78 136.27 128.45 103.23 78.70 67.96 62.80 52.34 29.95
f1 (ppm)

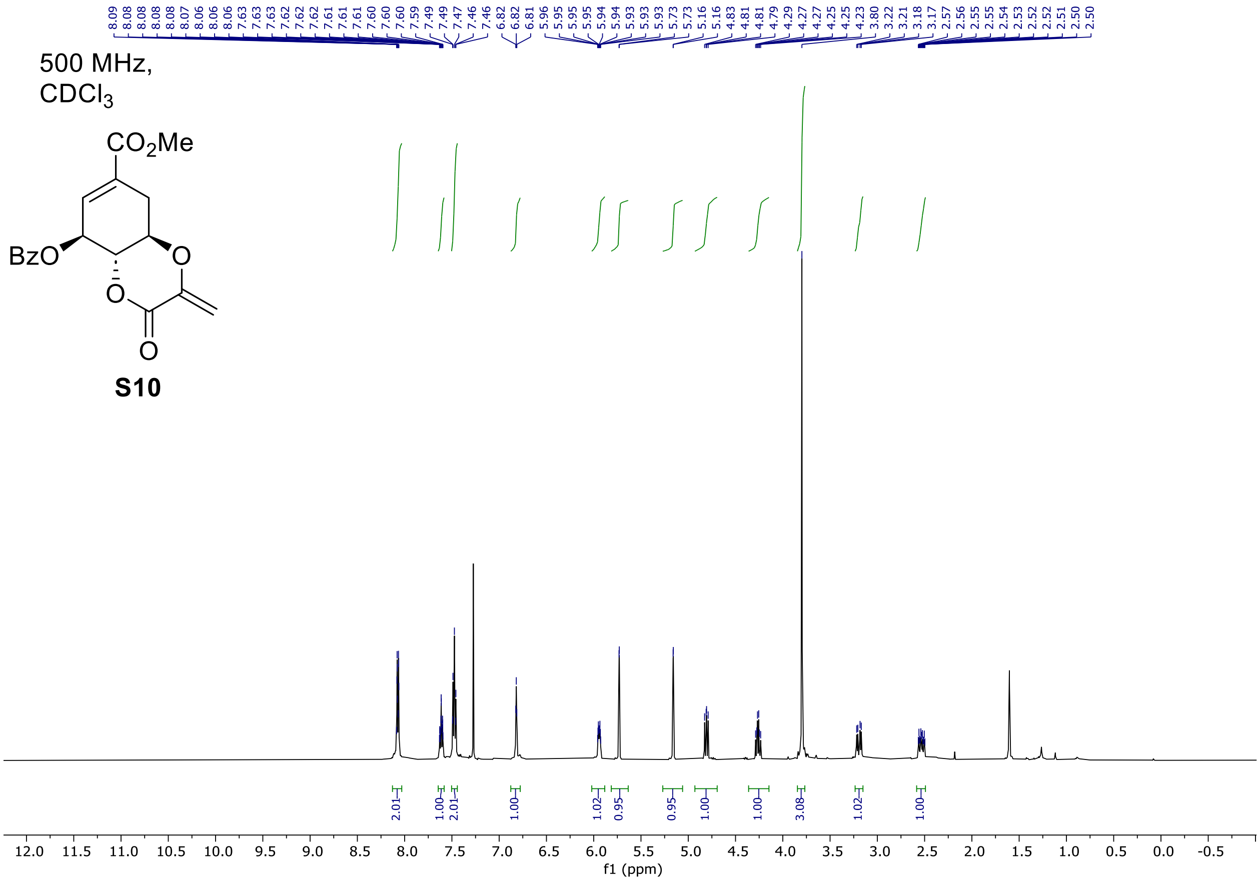
500 MHz,
$CDCl_3$
CO_2Me
BzO
O
O
O
S10
8.09 8.08 8.08 8.08 8.08 8.07 8.06 8.06 8.06 7.63 7.63 7.63 7.62 7.62 7.62 7.61 7.61 7.61 7.60 7.60 7.60 7.59 7.49 7.49 7.47 7.46 6.82 6.82 6.81 5.96 5.95 5.95 5.95 5.94 5.94 5.93 5.93 5.93 5.73 5.73 5.16 5.16 4.83 4.81 4.81 4.79 4.29 4.27 4.27 4.25 4.25 4.23 3.80 3.22 3.21 3.18 3.17 2.57 2.56 2.55 2.55 2.54 2.53 2.52 2.52 2.51 2.50 2.50
2.01 1.00 2.01 1.00 1.02 0.95 0.95 1.00 1.00 3.08 1.02 1.00
f1 (ppm)

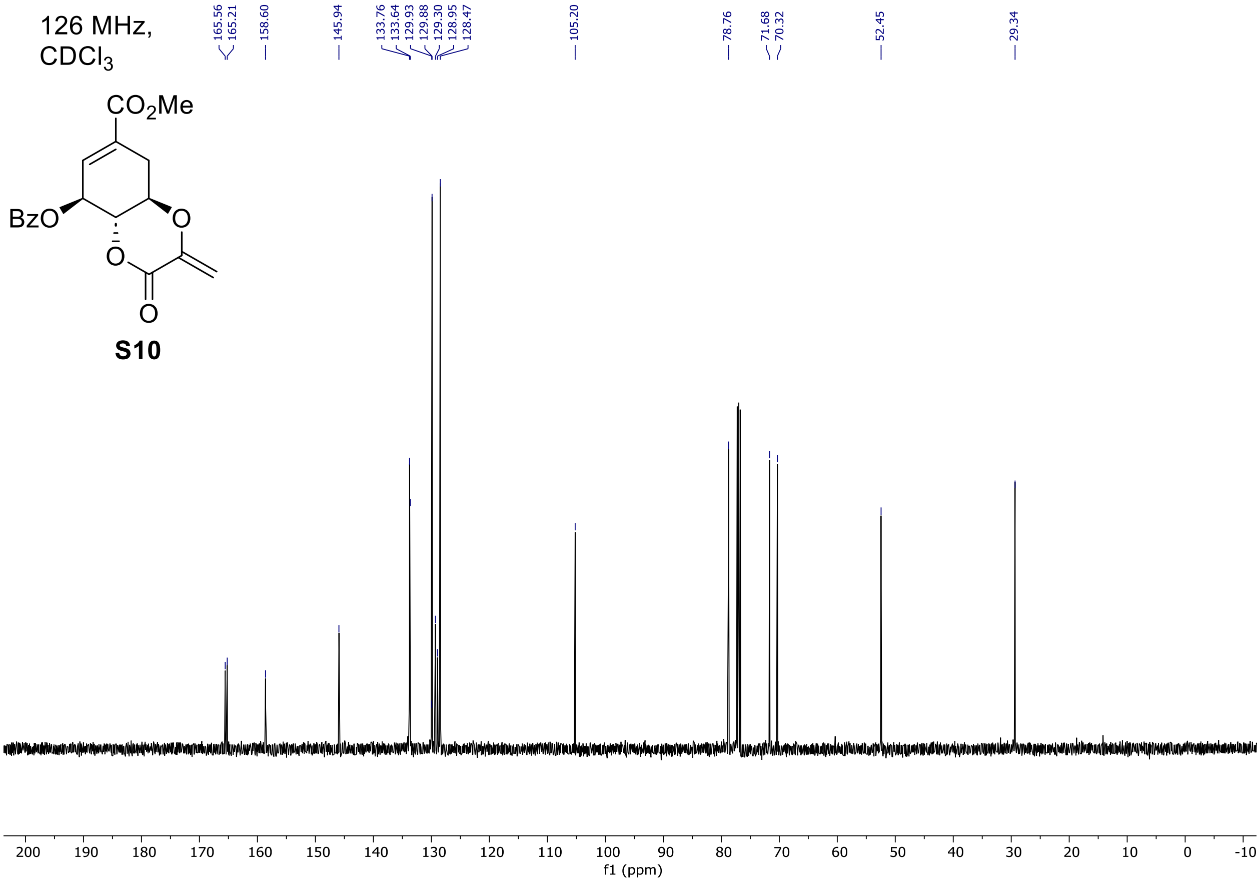
126 MHz,
$CDCl_3$
CO_2Me
BzO
O
O
O
S10
165.56 165.21 158.60 145.94 133.76 133.64 129.93 129.88 129.30 128.95 128.47 105.20 78.76 71.68 70.32 52.45 29.34
f1 (ppm)

500 MHz,
$CDCl_3$

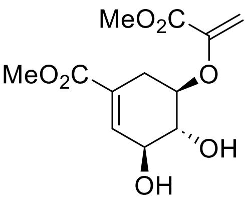

14

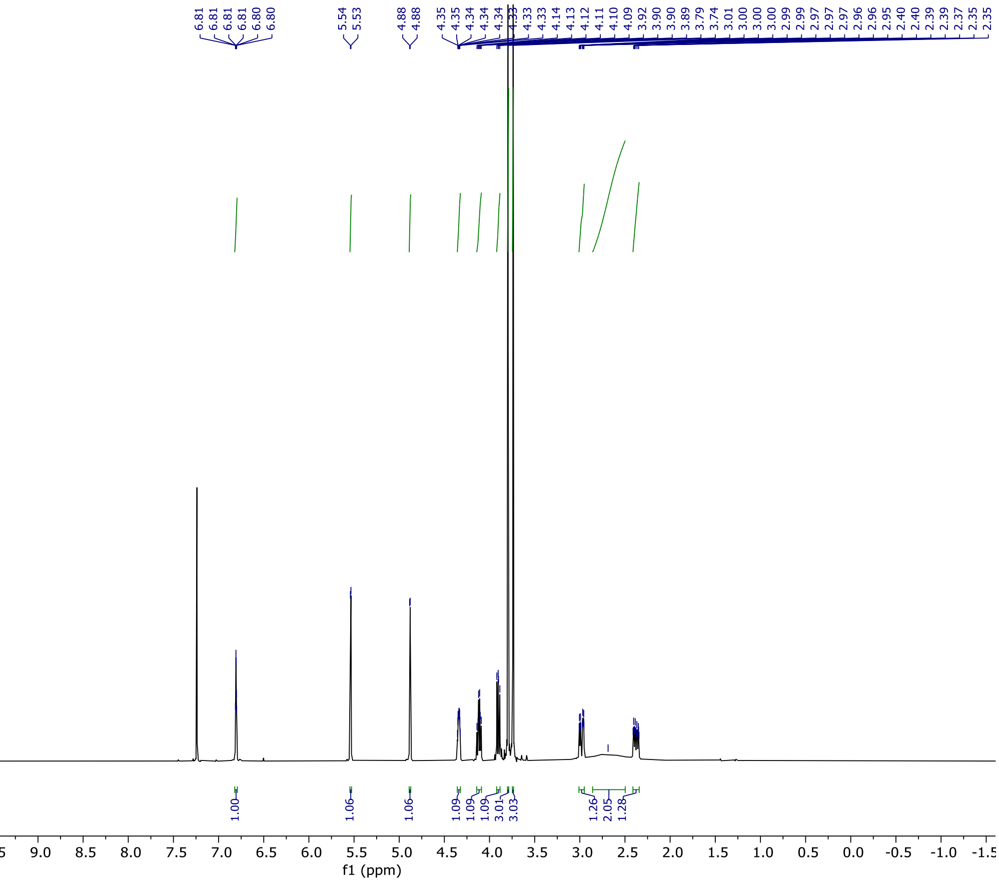

126 MHz,
$CDCl_3$

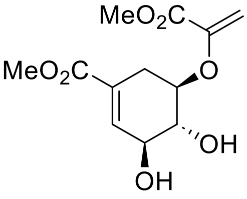

14

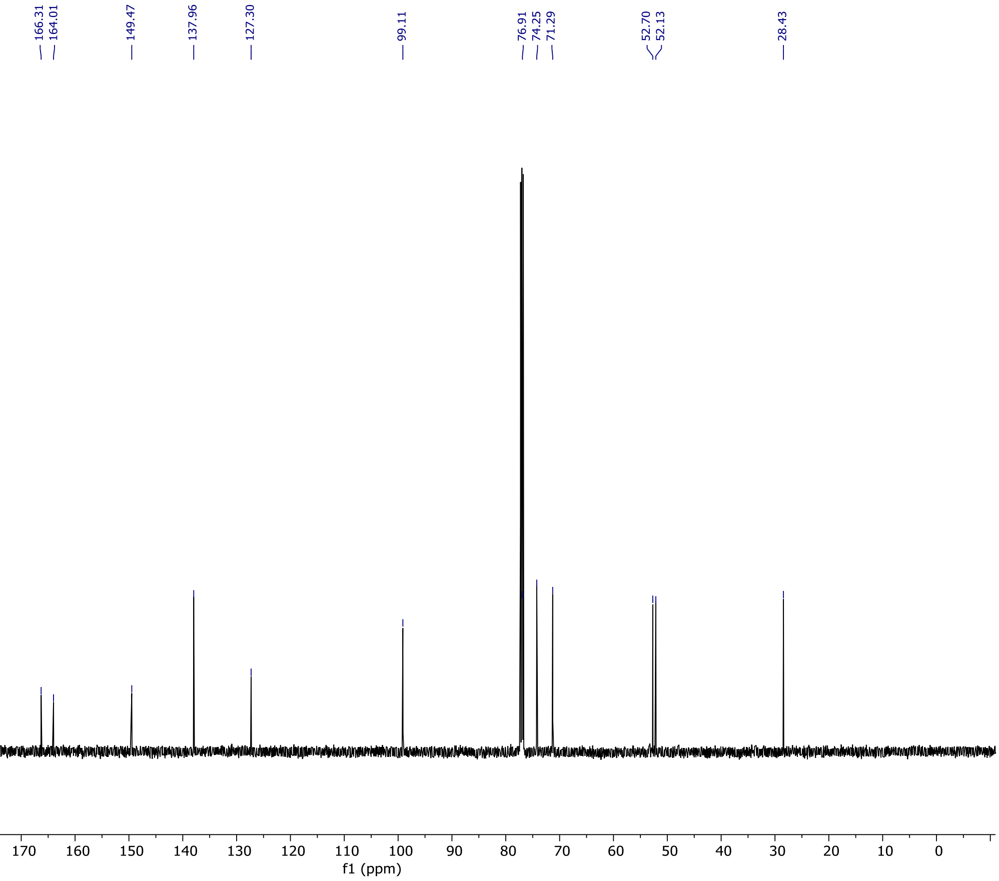

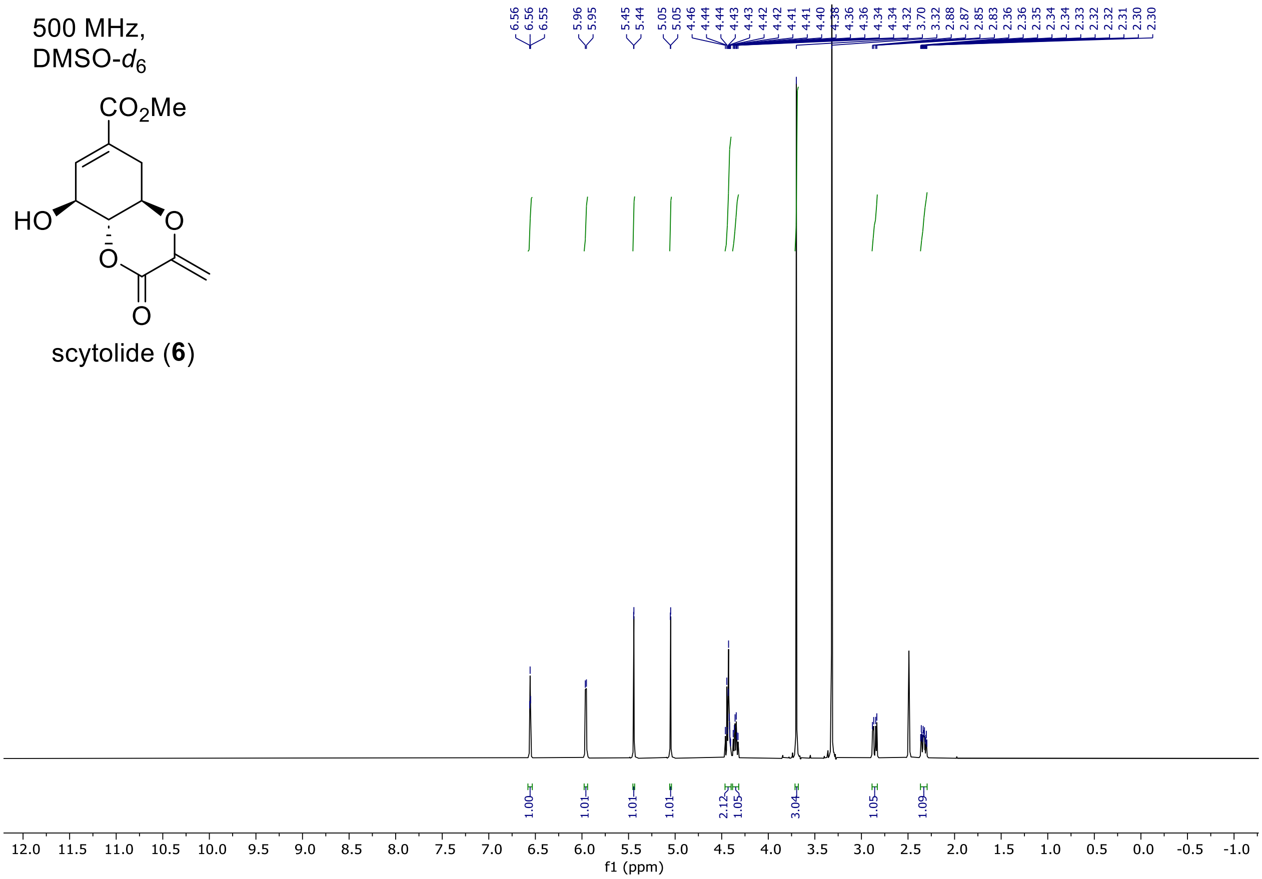
500 MHz,
DMSO-d_6
CO_2Me
HO
O
O
O
scytolide (**6**)
6.56 6.56 6.55 5.96 5.95 5.45 5.44 5.05 5.05 4.46 4.44 4.44 4.43 4.43 4.42 4.42 4.41 4.41 4.40 4.38 4.36 4.36 4.34 4.34 4.32 3.70 3.32 2.88 2.87 2.85 2.83 2.36 2.36 2.35 2.34 2.34 2.33 2.32 2.32 2.31 2.30 2.30
1.00 1.01 1.01 1.01 2.12 1.05 3.04 1.05 1.09
12.0 11.5 11.0 10.5 10.0 9.5 9.0 8.5 8.0 7.5 7.0 6.5 6.0 5.5 5.0 4.5 4.0 3.5 3.0 2.5 2.0 1.5 1.0 0.5 0.0 -0.5 -1.0
f1 (ppm)

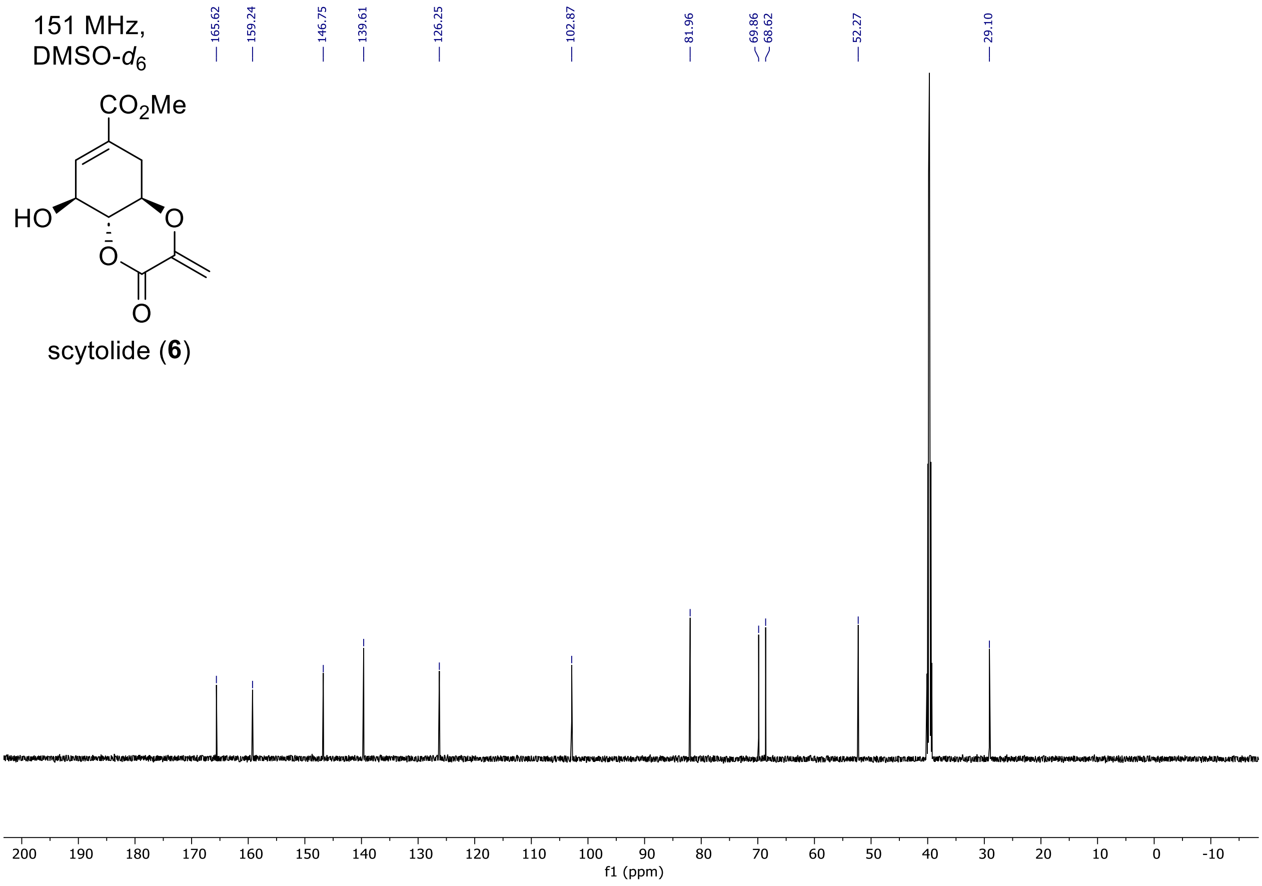
151 MHz,
DMSO-d_6
CO_2Me
HO
O
O
O
scytolide (**6**)
165.62 159.24 146.75 139.61 126.25 102.87 81.96 69.86 68.62 52.27 29.10
200 190 180 170 160 150 140 130 120 110 100 90 80 70 60 50 40 30 20 10 0 -10
f1 (ppm)

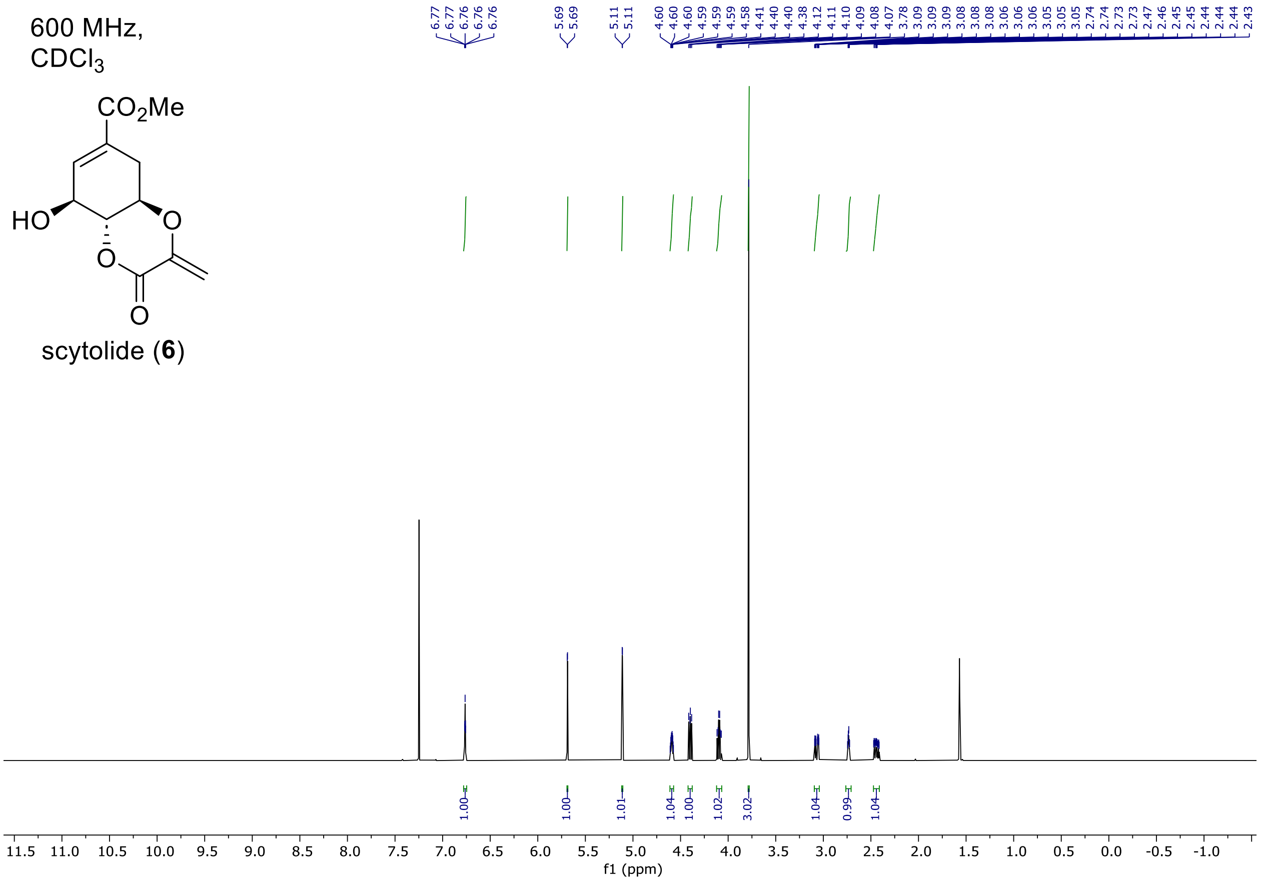
600 MHz,
CDCl3
CO2Me
HO
O
O
O
scytolide (6)
f1 (ppm)

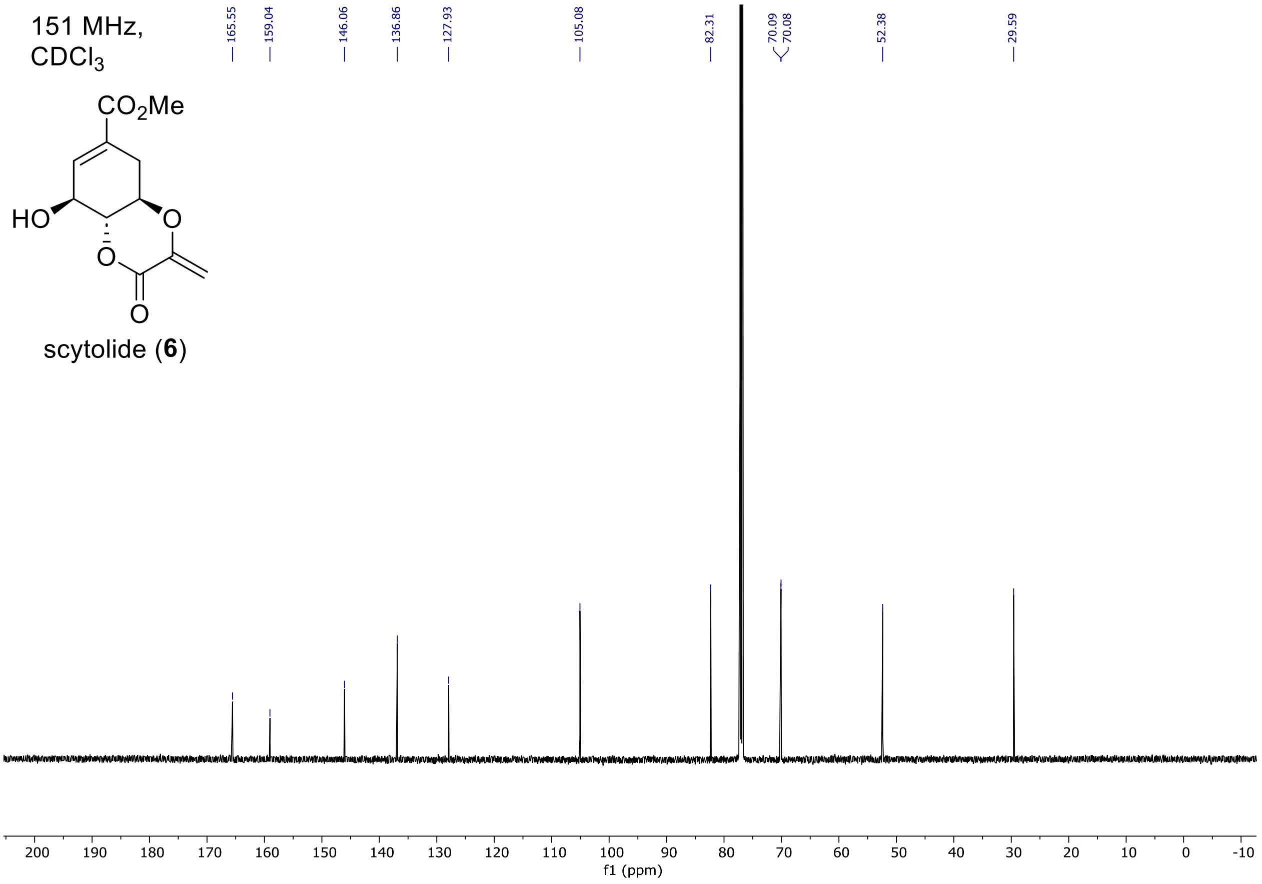
151 MHz,
CDCl3
CO2Me
HO
O
O
O
scytolide (6)
165.55
159.04
146.06
136.86
127.93
105.08
82.31
70.09
70.08
52.38
29.59
f1 (ppm)

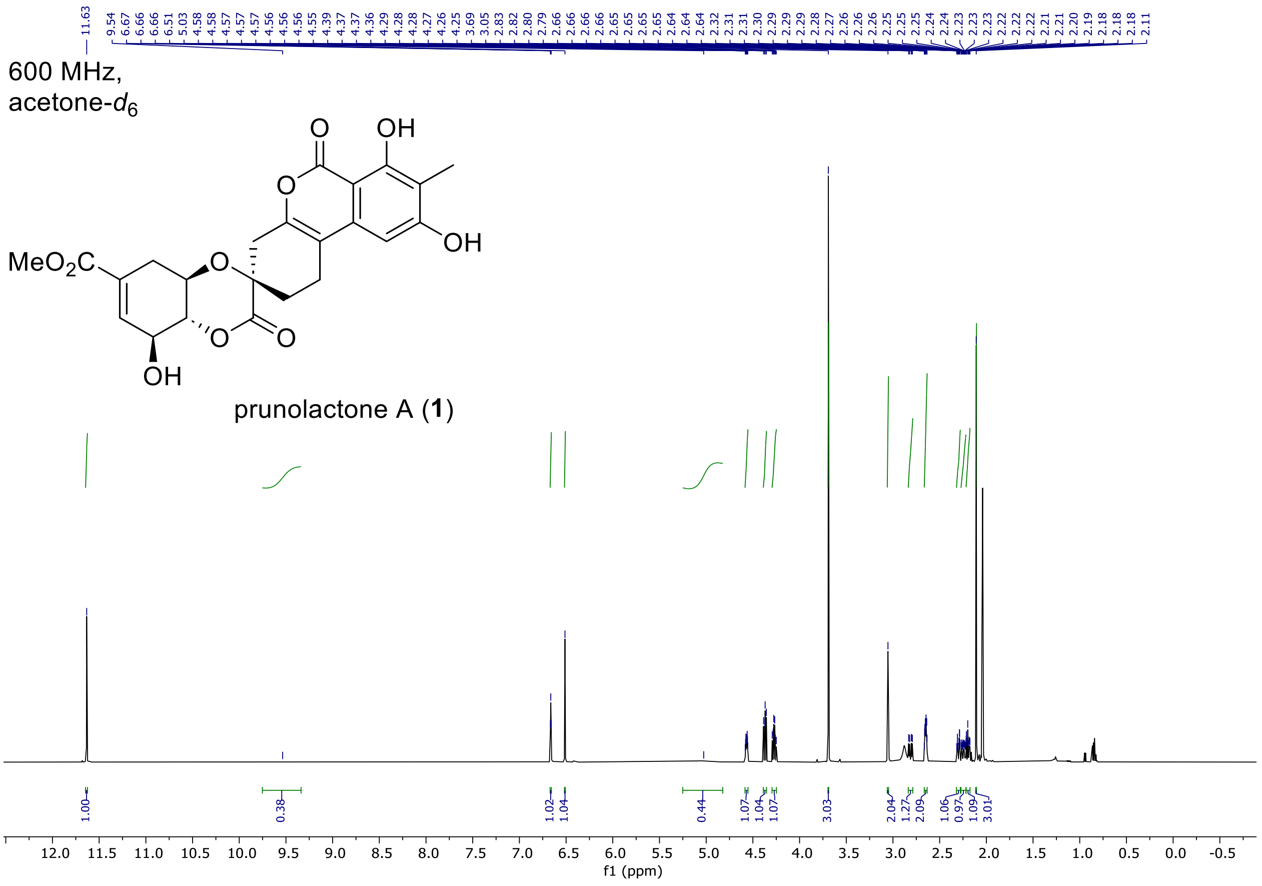
600 MHz,
acetone-d_6
prunolactone A (1)

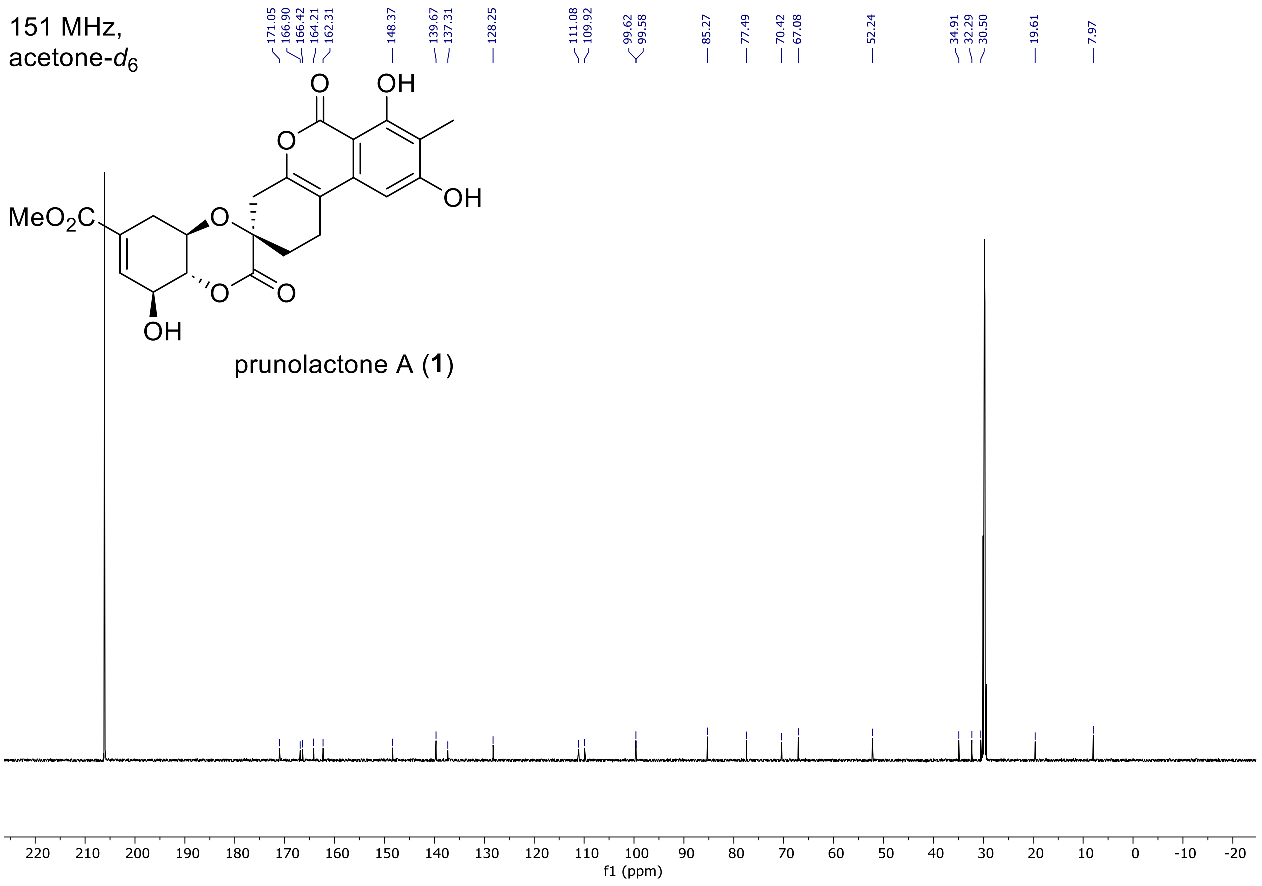
151 MHz,
acetone-d_6
171.05 166.90 166.42 164.21 162.31 148.37 139.67 137.31 128.25 111.08 109.92 99.62 99.58 85.27 77.49 70.42 67.08 52.24 34.91 32.29 30.50 19.61 7.97
prunolactone A (1)
f1 (ppm)

600 MHz, DMSO-d_6

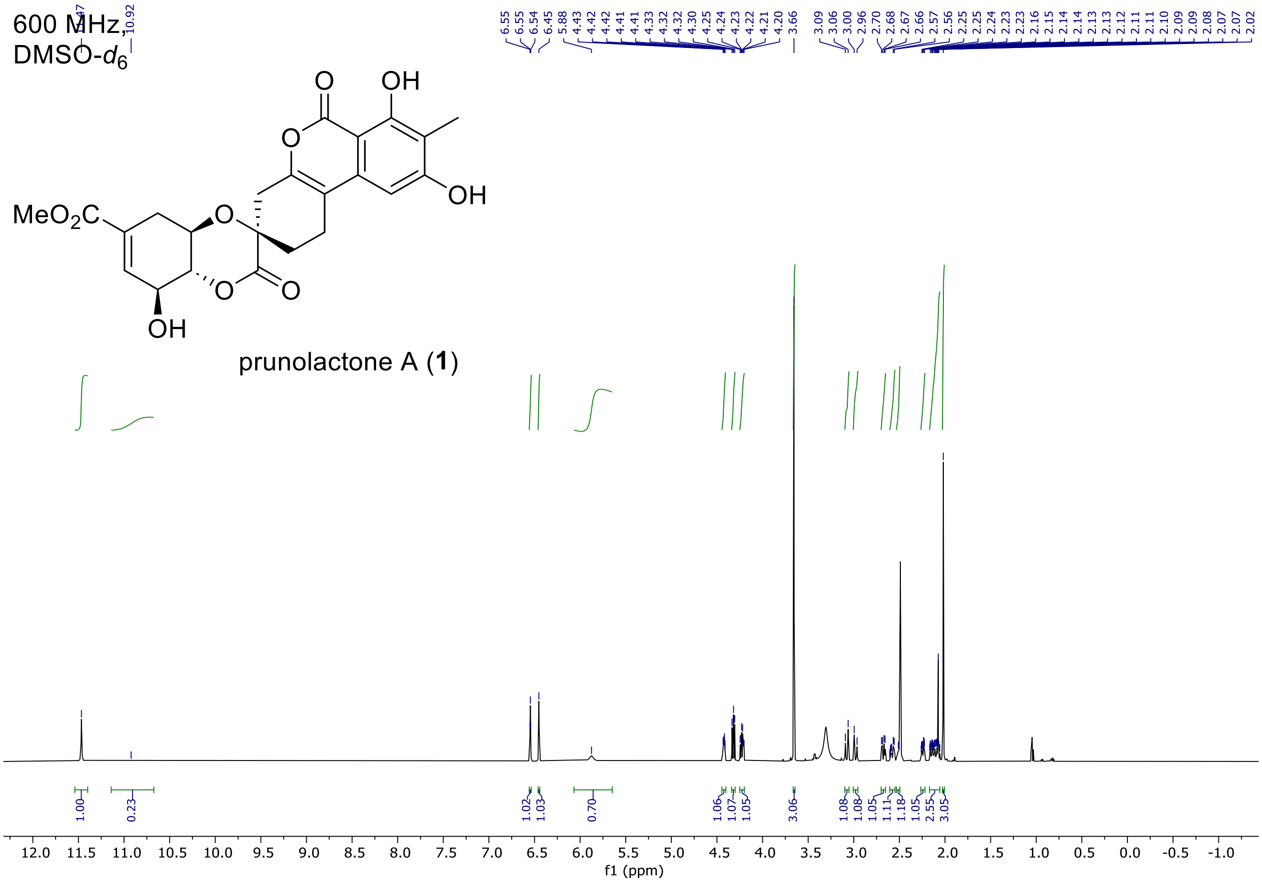

prunolactone A (**1**)

151 MHz, DMSO-d_6

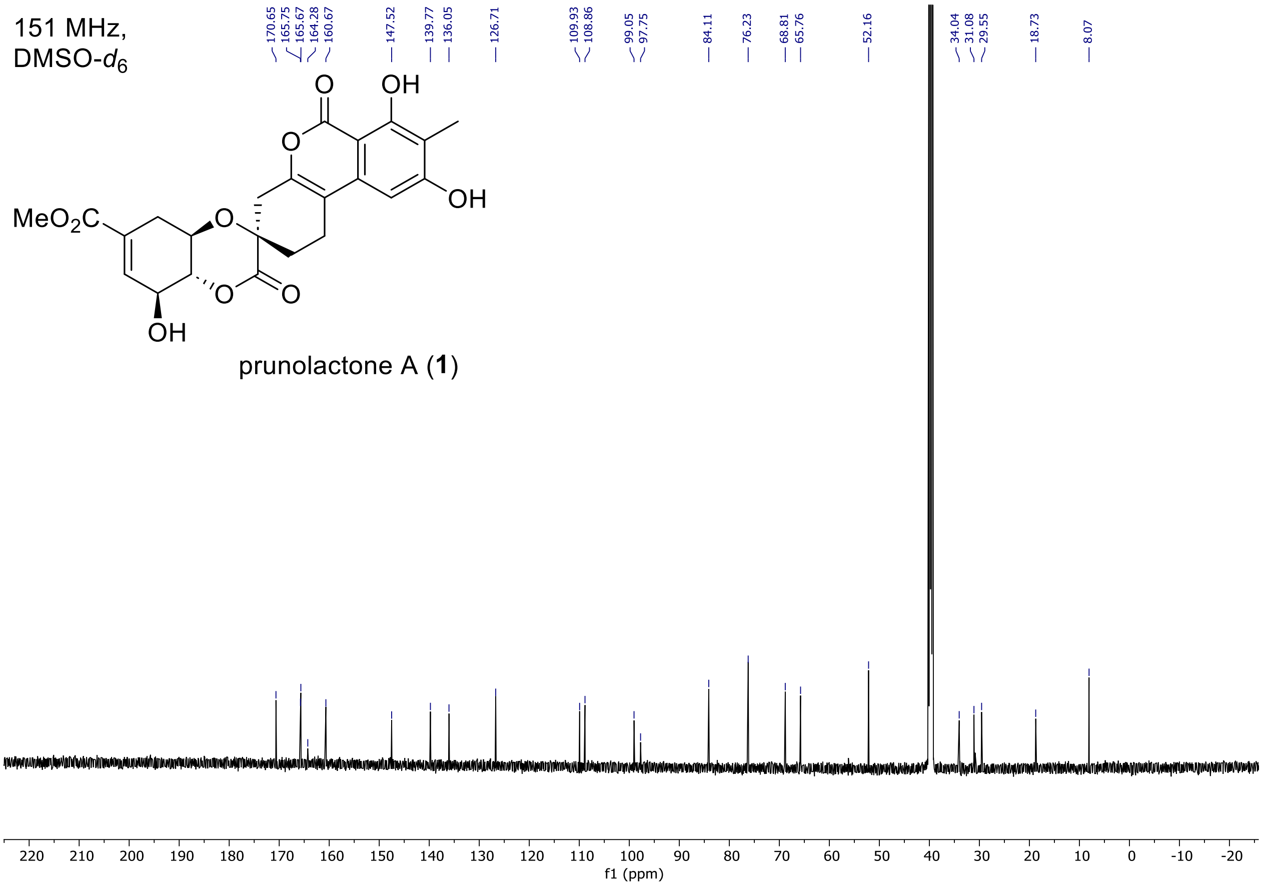

prunolactone A (**1**)

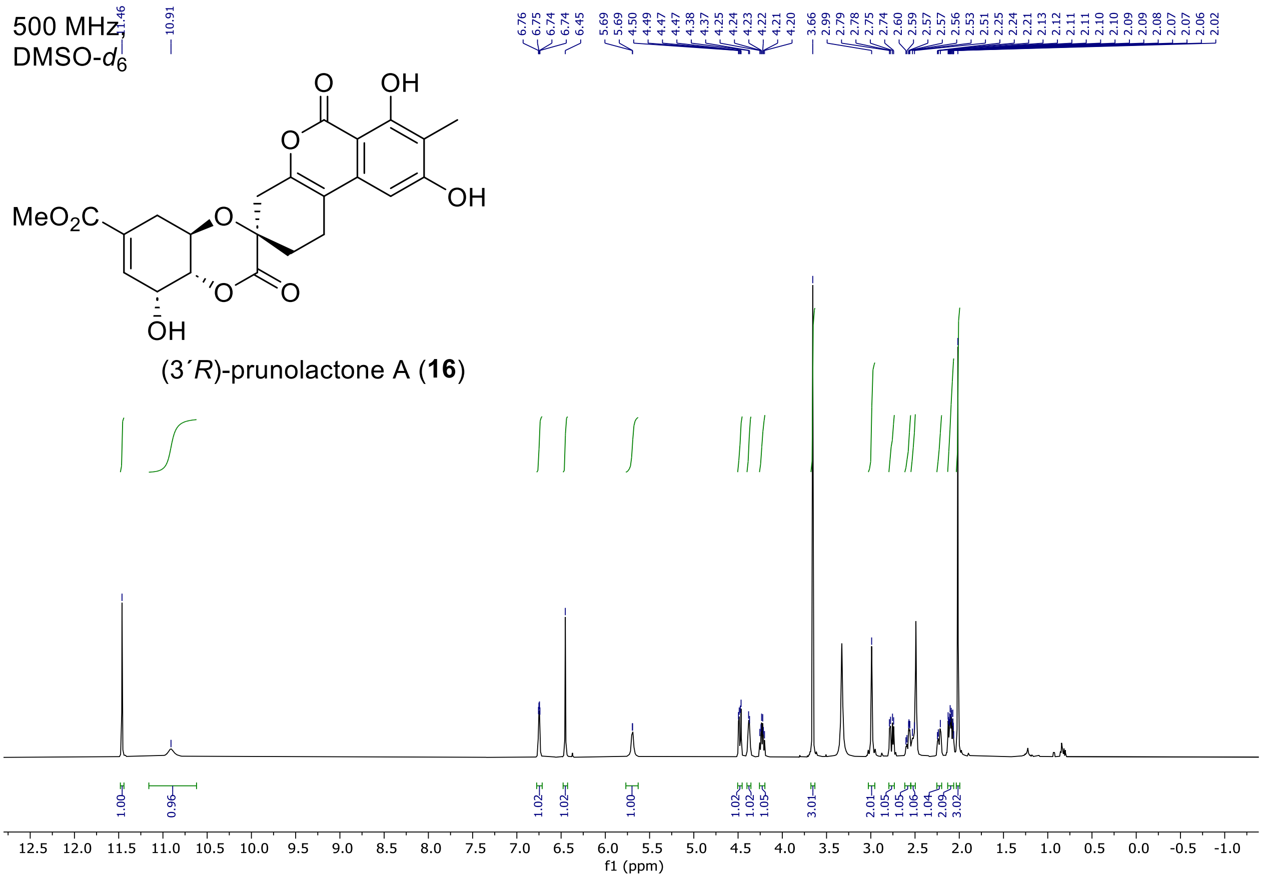
500 MHz, DMSO-d6
(3´R)-prunolactone A (16)
MeO2C
OH
O
11.46
10.91
6.76 6.75 6.74 6.74 6.45 5.69 5.69 4.50 4.49 4.47 4.47 4.38 4.37 4.25 4.24 4.23 4.22 4.21 4.20 3.66 2.99 2.79 2.78 2.75 2.74 2.60 2.59 2.57 2.57 2.56 2.53 2.51 2.25 2.24 2.21 2.13 2.12 2.11 2.10 2.10 2.09 2.09 2.08 2.07 2.07 2.06 2.02
1.00 0.96 1.02 1.02 1.00 1.02 1.02 1.05 3.01 2.01 1.05 1.05 1.06 1.04 2.09 3.02
f1 (ppm)

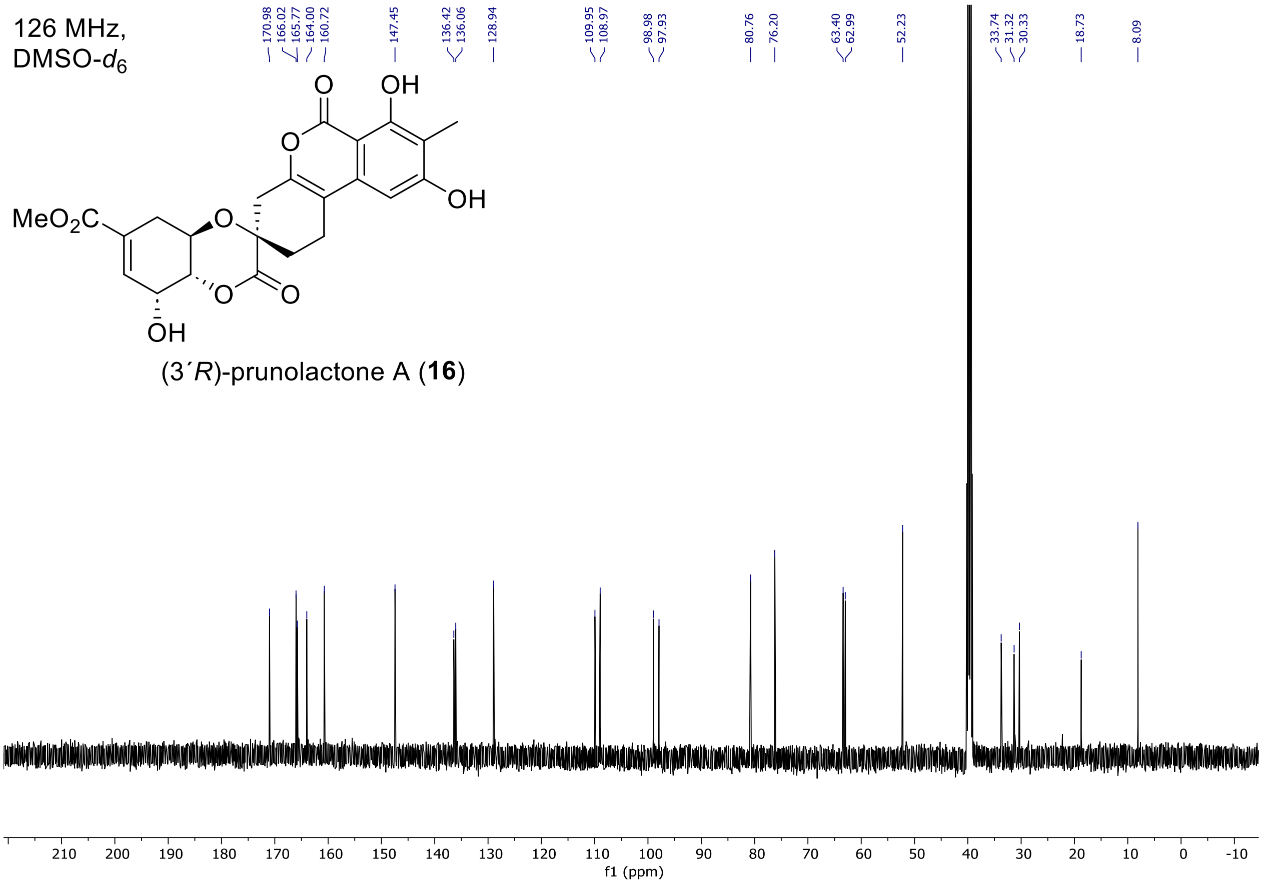
126 MHz, DMSO-d6
(3´R)-prunolactone A (16)
MeO2C
OH
O
170.98 166.02 165.77 164.00 160.72 147.45 136.42 136.06 128.94 109.95 108.97 98.98 97.93 80.76 76.20 63.40 62.99 52.23 33.74 31.32 30.33 18.73 8.09
f1 (ppm)

4 NMR Data of Prunolactone A (1)

4.1 Comparison of ^{1}H and ^{13}C NMR Spectra of Synthetic and Natural Prunolactone A (1)

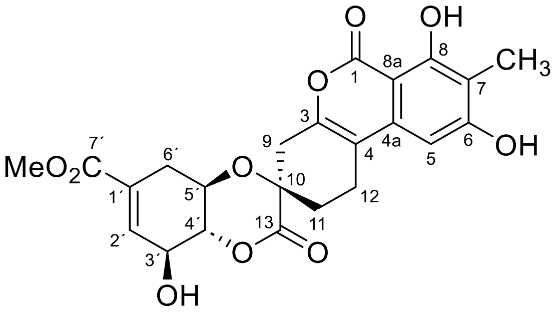

prunolactone A (**1**)

Position	^{1}H δ ppm (*J* Hz) synthetic[a)]	^{1}H δ ppm (*J* Hz) natural[b)]	^{13}C δ ppm synthetic[a)]	^{13}C δ ppm natural[b)]	$\Delta\,\delta_C$
1			166.9	167.0	0.1
3			148.4	148.4	0.0
4			109.9	110.0	0.1
4a			137.3	137.4	0.1
5	6.51, s	6.57, s	99.6	99.8	0.2
6			164.2	164.4	0.2
7			111.1	111.1	0.0
8			162.3	162.4	0.1
8a			99.6	99.6	0.0
7-CH_3	2.11, s	2.12, s	8.0	8.0	0.0
9	3.05, s	3.07, s	34.9	35.0	0.1
10			77.5	77.6	0.1
11	2.32–2.28, m<br>2.22–2.17, m	2.30, m<br>2.19, m	32.3	32.4	0.1
12	2.67–2.63, m	2.67, m	19.6	19.7	0.1
13			171.1	171.1	0.0
1′			128.3	128.3	0.0
2′	6.66, t (2.3)	6.68, t (2.3)	139.7	139.8	0.1
3′	4.59–4.54, m	4.58, brs	70.4	70.5	0.1
4′	4.37, dd (9.9, 8.0)	4.39, dd (10.0, 8.0)	85.3	85.3	0.0
5′	4.27, td (9.9, 6.2)	4.29, td (10.0, 6.2)	67.1	67.2	0.1
6′	2.81, dd (17.5, 6.2)<br>2.27–2.22, m	2.81, dd (17.5, 6.2)<br>2.25, m	30.5	30.6	0.1
7′			166.4	166.5	0.1
–OCH_3	3.69, s	3.70, s	52.2	52.3	0.1
–OH	11.63, s	11.66, s			

a) ^{1}H (600 MHz), ^{13}C (151 MHz); acetone-d_6. b) ^{1}H (400 MHz), ^{13}C (100 MHz); acetone-d_6.[6]

4.2 2D NMR Spectra of Prunolactone A (1)

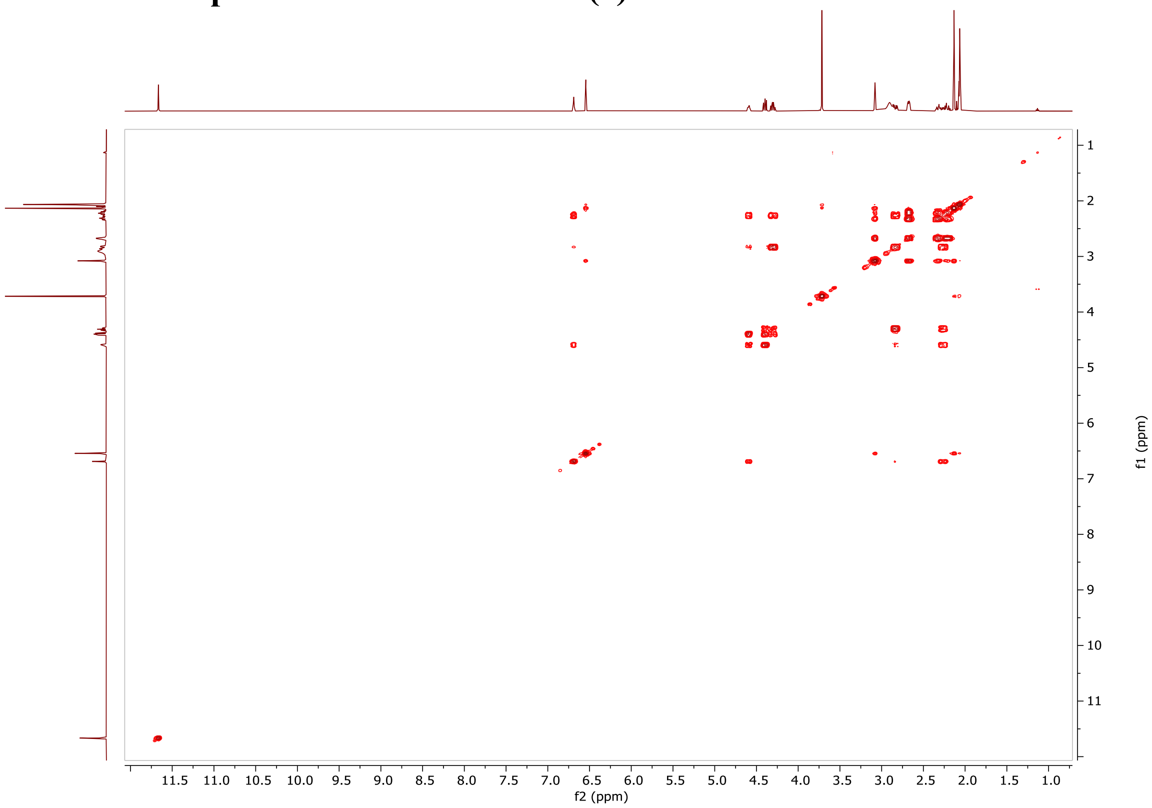

Figure S1. ^{1}H-^{1}H COSY (acetone-d_6) spectrum of prunolactone A (**1**).

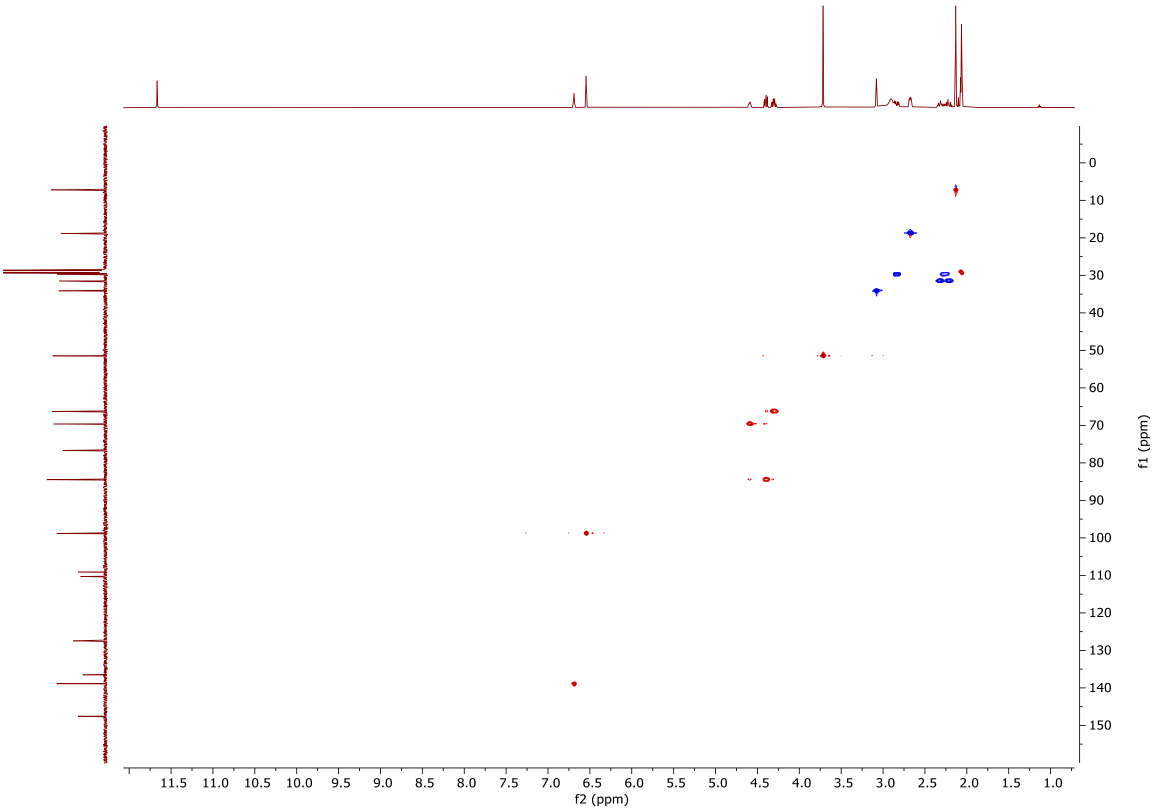

Figure S2. HSQC (acetone-d_6) spectrum of prunolactone A (**1**).

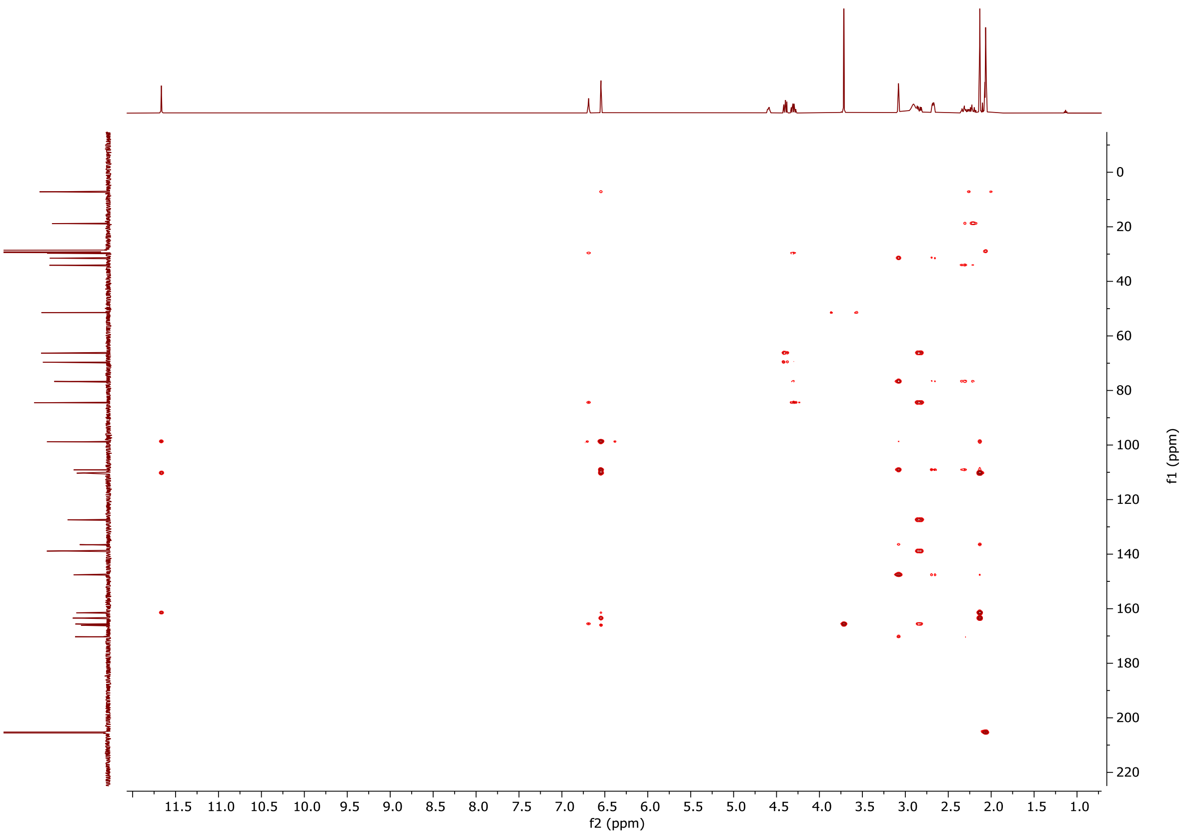

Figure S3. HMBC (acetone-d_6) spectrum of prunolactone A (**1**).

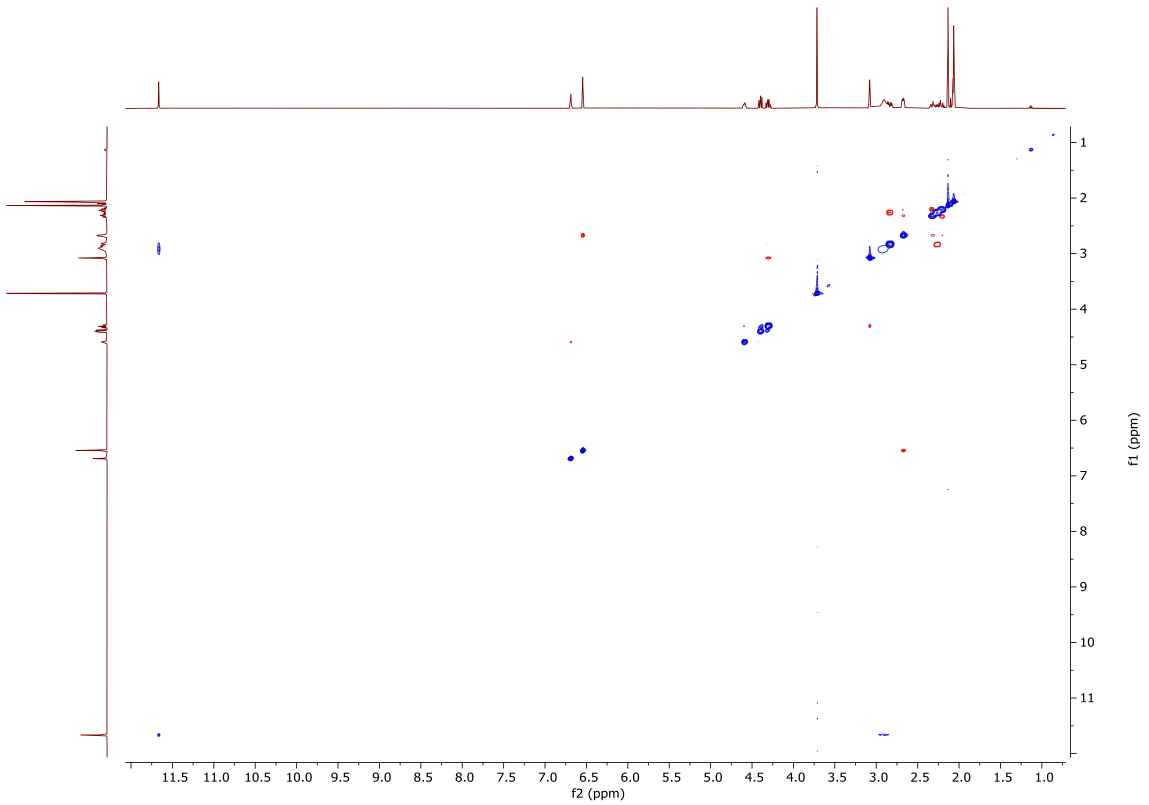

Figure S4. NOESY (acetone-d_6) spectrum of prunolactone A (**1**).

5 Additional Analysis of Scytolide (6)

5.1 X-Ray Analysis of Scytolide (6)

Table S2. Experimental details for crystallographic measurements of scytolide (**6**).

Crystal data	
Chemical formula	$C_{11}H_{12}O_6$
M_r	240.21
Crystal system, space group	Monoclinic, $P2_1$
Temperature (K)	150
a, *b*, *c* (Å)	7.3834(2), 11.2092(3), 12.8323(3)
β (°)	92.968(1)
V (Å^3)	1060.60(5)
Z	4
Radiation type	Mo*K*α
μ (mm^{-1})	0.12
Crystal size (mm)	0.56 × 0.23 × 0.18

Data collection	
Diffractometer	Bruker D8 - Venture
Absorption correction	Multi-scan<br>*SADABS2016*/2 - Bruker AXS area detector scaling and absorption correction
T_{min}, T_{max}	0.714, 0.746
No. of measured, independent and observed [$I > 2\sigma(I)$] reflections	31936, 5171, 5022
R_{int}	0.037
$(\sin \theta/\lambda)_{max}$ (Å^{-1})	0.667

Refinement	
$R[F^2 > 2\sigma(F^2)]$, $wR(F^2)$, S	0.031, 0.083, 1.07
No. of reflections	5171
No. of parameters	315
No. of restraints	266
H-atom treatment	H atoms treated by a mixture of independent and constrained refinement
$\Delta\rho_{max}$, $\Delta\rho_{min}$ (e Å^{-3})	0.32, −0.16
Absolute structure	Flack x determined using 2305 quotients [(I+)-(I-)]/[(I+)+(I-)] (Parsons, Flack and Wagner, Acta Cryst. B69 (2013) 249-259).
Absolute structure parameter	−0.16(17)

Computer programs: *SHELXL2019*/1 (Sheldrick, 2019).

Table S3. Hydrogen-bond geometry (Å, º) for scytolide (**6**).

D—H···*A*	*D*—H	H···*A*	*D*···*A*	*D*—H···*A*
O4—H4*O*···O9	0.92 (3)	1.89 (3)	2.772 (2)	159 (3)
O10—H10*O*···O3	0.80 (1)	1.94 (1)	2.7357 (19)	171 (3)

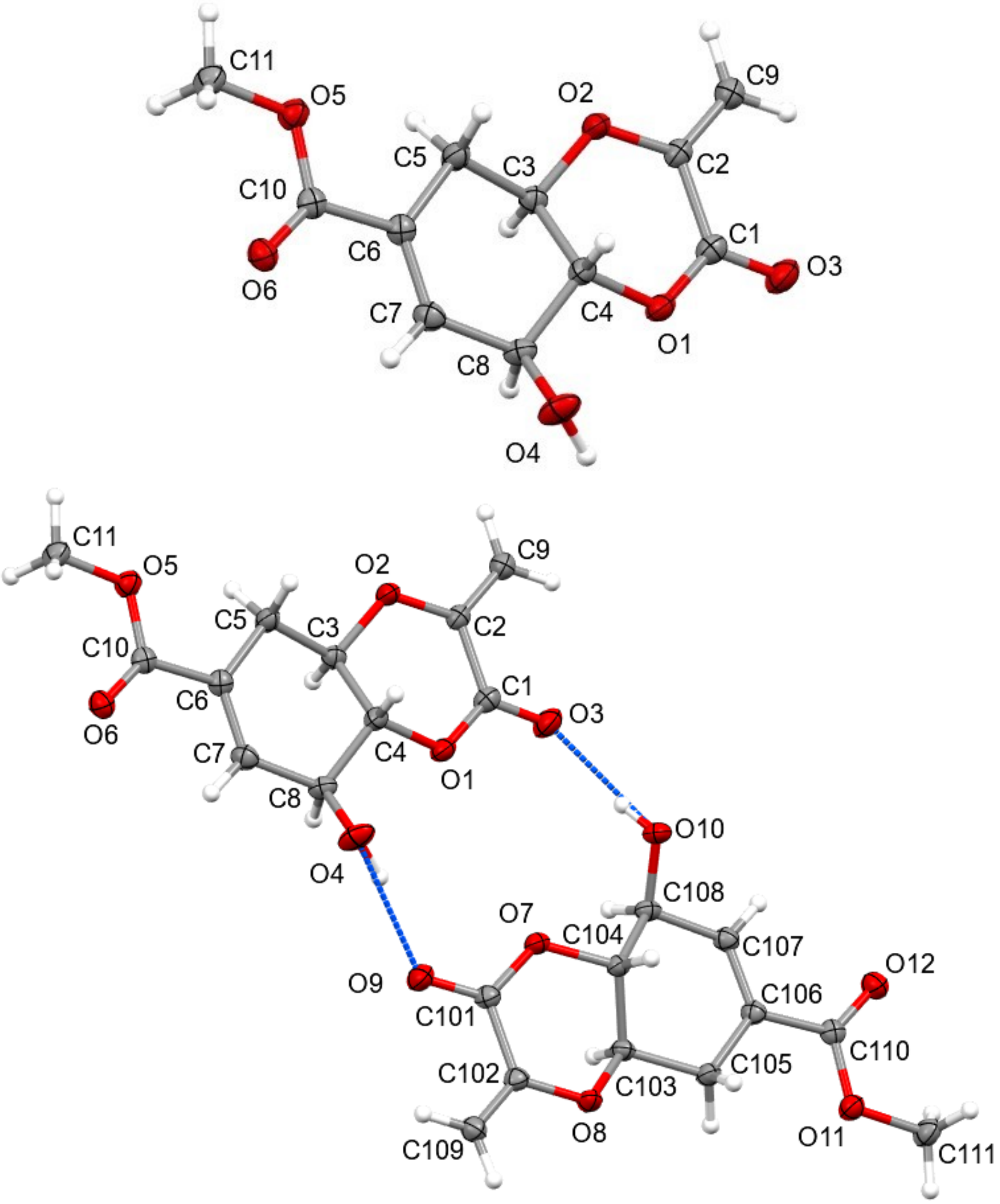

Figure S5. ORTEP diagrams of scytolide (**6**); Ellipsoid probability level 50%.

Note: The sample of scytolide (**6**) for X-ray analysis was crystallized from ethyl acetate/hexanes at 25 °C for a period of 4 days.

5.2 ECD Analysis of Scytolide (6)

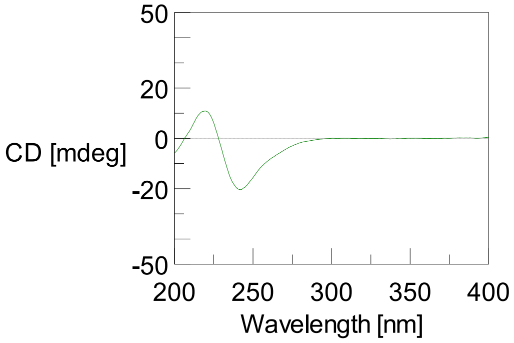

Figure S6. ECD spectrum of scytolide (**6**).
ECD (c 0.017, MeOH) λ_{max} ($\Delta\varepsilon$) 220 (+4.63), 242 (−8.74).

Note: ECD Spectrum of scytolide (**6**) was in agreement with the previously published experimental and calculated spectra[7].

6 DFT Calculations

6.1 Computational Details

The density functional theory (DFT) calculations were performed with ωB97X-D[8] functional and 6-311+g(2d,p) basis set with CPCM solvation model (THF), while optimizations were carried out with 6-31g(d,p) basis set. This protocol was previously validated for similar DA reaction.[9] We performed conformational search of all reaction components by generating 100 random conformers using RDKit[10] python package. Each conformer was optimized with AIMNet2 neural network (NN) potential (*aimnet2_wb97m-d3_0.jpt* parameter set, available from https://github.com/isayevlab/aimnet2),[11] with PCM[12] energy correction based on the AIMNet2 charges ($\varepsilon = \infty$; 110-point Lebedev grid; atomic radii(Å):[13] H=1.3, C=2.00, O=1.72), and Gaussian 16 geometry optimizer[14]. For locating the transition states, optimization was at first constrained ($r_{\text{formed bonds}}$=2.0–2.2 Å) and then optimized freely via eigenvalue following algorithm. We selected the lowest conformer for each compound/TS. The guesses that did not lead to correct transition states were discarded. Finally, the energies were recalculated at the target level the Gaussian 16 program.

6.2 Descriptions of File Formats

Structure of the individual entry in the XYZ file follows this template:

number_of_atoms
name charge spin_multiplicity key1=value1 key2=value2 ....
atom1, x1, y1, z1
atom2, x2, y2, z2
..., ..., ..., ...

Description of selected keys:
route: route section of the Gaussian program (includes the used method and basis set). Replace "\u0020" by a single space character.
HF: final SCF energy
ZeroPoint: Zero-point energy correction [Hartree]
CorrGibbs: Gibbs free energy correction (298 K, 1 atm) [Hartree]
NImag: Number of imaginary vibrational frequencies

The attached gzipped log file (conformers_prunolactone_A_TS.log.gz; calculated with AIMNet2+PCM method) mimics the Gaussian program output for vibrational frequency analysis.

7 References

(1) Germain, A. R.; Bruggemeyer, D. M.; Zhu, J.; Genet, C.; O'Brien, P.; Porco, J. A. Synthesis of the Azaphilones (+)-Sclerotiorin and (+)-8-*O*-Methylsclerotiorinamine Utilizing (+)-Sparteine Surrogates in Copper-Mediated Oxidative Dearomatization. *J. Org. Chem.* **2011**, *76*, 2577–2584.

(2) Kratochvíl, J.; Novák, Z.; Ghavre, M.; Nováková, L.; Růžička, A.; Kuneš, J.; Pour, M. Fully Substituted Pyranones via Quasi-Heterogeneous Genuinely Ligand-Free Migita-Stille Coupling of Iodoacrylates. *Org. Lett.* **2015**, *17*, 520–523.

(3) Milzarek, T. M.; Gulder, T. A. M. Chemo-Enzymatic Total Synthesis of the Spirosorbicillinols. *Commun. Chem.* **2023**, *6* (187).

(4) Wørmer, G. J.; Hansen, B. K.; Palmfeldt, J.; Poulsen, T. B. A Cyclopropene Electrophile that Targets Glutathione S-Transferase Omega-1 in Cells. *Angew. Chem. Int. Edit.* **2019**, *58* (34), 11918–11922.

(5) Chouinard, P. M.; Bartlett, P. A. Conversion of Shikimic Acid to 5-Enolpyruvylshikimate 3-Phosphate. *J. Org. Chem.* **1986**, *51* (1), 75–78.

(6) Zhang, X.-Q.; Lu, Z.-H.; Tang, G.-M.; Duan, L.-P.; Wang, Z.-H.; Guo, Z.-Y.; Proksch, P. Prunolactones A-G, Proangiogenic Isocoumarin Derivatives with an Unusual 6/6/6/6/6 Spiropentacyclic Skeleton from the Endophytic Fungus *Phomopsis prunorum*. *Bioorg. Chem.* **2023**, *141*.

(7) Mazzeo, G.; Santoro, E.; Andolfi, A.; Cimmino, A.; Troselj, P.; Petrovic, A. G.; Superchi, S.; Evidente, A.; Berova, N. Absolute Configurations of Fungal and Plant Metabolites by Chiroptical Methods. ORD, ECD, and VCD Studies on Phyllostin, Scytolide, and Oxysporone. *J. Nat. Prod.* **2013**, *76*, 588–599.

(8) Chai, J.-D.; Head-Gordon, M. Long-Range Corrected Hybrid Density Functionals with Damped Atom-Atom Dispersion Corrections. *Phys. Chem. Chem. Phys.* **2008**, *10* (44), 6615–6620.

(9) Antal, R.; Staś, M.; Perdomo, S. M.; Štemberová, M.; Brůža, Z.; Matouš, P.; Kratochvíl, J.; Růžička, A.; Rulíšek, L.; Kuneš, J.; Kočovský, P.; Andris, E.; Pour, M. Synthesis of Highly Polarized [3]Dendralenes and their Diels-Alder Reactions. *Org. Chem. Front.* **2023**, *10,* 5568–5578.

(10) RDKit: Open-source cheminformatics, version 2024.03.5. https://www.rdkit.org

(11) Anstine, D.; Zubatyuk, R.; Isayev O. AIMNet2: A Neutral Network Potential to Meet your Neutral, Charged, Organic, and Elemental-Organic Needs. *ChemRxiv* **2024**.

(12) Garcia-Ratés, M.; Neese, F. Effect of the Solute Cavity on the Solvation Energy and its Derivatives within the Framework of the Gaussian Charge Scheme. *J. Comput. Chem.* **2020**, *41,* 922–939.

(13) Klamt, A.; Jonas, V.; Bürger, T.; Lohrenz, J.C. Refinement and Parametrization of COSMO-RS. *J. Phys. Chem. A* **1998,** *102,* 5074–5085.

(14) Gaussian 16, Revision C.01, Frisch, M. J.; Trucks, G. W.; Schlegel, H. B.; Scuseria, G. E.; Robb, M. A.; Cheeseman, J. R.; et al. Gaussian, Inc., Wallingford CT, **2016**.
